# Challenges at the *APOE* locus: a robust quality control approach for accurate *APOE* genotyping

**DOI:** 10.1186/s13195-022-00962-4

**Published:** 2022-02-04

**Authors:** Michael E. Belloy, Sarah J. Eger, Yann Le Guen, Vincent Damotte, Shahzad Ahmad, M. Arfan Ikram, Alfredo Ramirez, Anthoula C. Tsolaki, Giacomina Rossi, Iris E. Jansen, Itziar de Rojas, Kayenat Parveen, Kristel Sleegers, Martin Ingelsson, Mikko Hiltunen, Najaf Amin, Ole Andreassen, Pascual Sánchez-Juan, Patrick Kehoe, Philippe Amouyel, Rebecca Sims, Ruth Frikke-Schmidt, Wiesje M. van der Flier, Jean-Charles Lambert, for the European Alzheimer & Dementia BioBank (EADB), Zihuai He, Summer S. Han, Valerio Napolioni, Michael D. Greicius

**Affiliations:** 1 Department of Neurology and Neurological Sciences – Greicius lab, Stanford University, 290 Jane Stanford Way, Stanford, CA 94304 USA; 2 Univ. Lille, Inserm, CHU Lille, Institut Pasteur de Lille, U1167-RID-AGE Facteurs de risque et déterminants moléculaires des maladies liées au vieillissement, Lille, France; 3 Department of Epidemiology, ErasmusMC, Rotterdam, The Netherlands; 4 Division of Systems Biomedicine and Pharmacology, Leiden Academic Centre for Drug Research, Leiden University, Leiden, The Netherlands; 5 Division of Neurogenetics and Molecular Psychiatry, Department of Psychiatry and Psychotherapy, Faculty of Medicine and University Hospital Cologne, University of Cologne, Cologne, Germany; 6 Department of Neurodegenerative diseases and Geriatric Psychiatry, Medical Faculty, University Hospital Bonn, Bonn, Germany; 7 Department of Psychiatry & Glenn Biggs Institute for Alzheimer’s and Neurodegenerative Diseases, San Antonio, TX USA; 8 German Center for Neurodegenerative Diseases (DZNE), Bonn, Germany; 9 Cluster of Excellence Cellular Stress Responses in Aging-associated Diseases (CECAD), University of Cologne, Cologne, Germany; 10 1st Department of Neurology, AHEPA Hospital, Aristotle University of Thessaloniki, Athens, Greece; 11 Unit of Neurology V and Neuropathology, Fondazione IRCCS Istituto Neurologico Carlo Besta, Milan, Italy; 12 Alzheimer Center Amsterdam, Department of Neurology, Amsterdam Neuroscience, Vrije Universiteit Amsterdam, Amsterdam UMC, Amsterdam, The Netherlands; 13 Department of Complex Trait Genetics, Center for Neurogenomics and Cognitive Research, Amsterdam Neuroscience, Vrije University, Amsterdam, The Netherlands; 14 Research Center and Memory Clinic, ACE Alzheimer Center Barcelona, Universitat Internacional de Catalunya, Barcelona, Spain; 15 Networking Research Center on Neurodegenerative Diseases (CIBERNED), Instituto de Salud Carlos III, Madrid, Spain; 16 Complex Genetics of Alzheimer’s Disease Group, Center for Molecular Neurology, VIB, Antwerp, Belgium; 17 Department of Biomedical Sciences, University of Antwerp, Antwerp, Belgium; 18 Department of Public Health and Carins Sciences/Geriatrics, Uppsala University, Uppsala, Sweden; 19 Institute of Biomedicine, University of Eastern Finland, Yliopistonranta 1E, 70211 Kuopio, Finland; 20 Nuffield Department of Population Health Oxford University, Oxford, UK; 21 NORMENT Centre, Division of Mental Health and Addiction, Oslo University Hospital, Oslo, Norway; 22 Institute of Clinical Medicine, University of Oslo, Oslo, Norway; 23 CIBERNED, Network Center for Biomedical Research in Neurodegenerative Diseases, National Institute of Health Carlos III, Madrid, Spain; 24 Neurology Service, Marqués de Valdecilla University Hospital (University of Cantabria and IDIVAL), Santander, Spain; 25 Translational Health Sciences, Bristol Medical School, University of Bristol, Bristol, UK; 26 Division of Psychological Medicine and Clinical Neuroscience, School of Medicine, Cardiff University, Wales, UK; 27 Department of Clinical Biochemistry, Copenhagen University Hospital – Rigshospitalet, Copenhagen, Denmark; 28 Department of Clinical Medicine, University of Copenhagen, Copenhagen, Denmark; 29 Quantitative Sciences Unit, Department of Medicine, Stanford University, Stanford, CA 94304 USA; 30 Department of Neurosurgery, Stanford University, Stanford, CA 94304 USA; 31 School of Biosciences and Veterinary Medicine, University of Camerino, 62032 Camerino, Italy

**Keywords:** Alzheimer’s disease (AD), Genetics, Haplotypes, *Apolipoprotein E* (*APOE*), rs439401, Novel approaches

## Abstract

**Background:**

Genetic variants within the *APOE* locus may modulate Alzheimer’s disease (AD) risk independently or in conjunction with APOE*2/3/4 genotypes. Identifying such variants and mechanisms would importantly advance our understanding of *APOE* pathophysiology and provide critical guidance for AD therapies aimed at *APOE*. The *APOE* locus however remains relatively poorly understood in AD, owing to multiple challenges that include its complex linkage structure and uncertainty in APOE*2/3/4 genotype quality. Here, we present a novel APOE*2/3/4 filtering approach and showcase its relevance on AD risk association analyses for the rs439401 variant, which is located 1801 base pairs downstream of *APOE* and has been associated with a potential regulatory effect on *APOE*.

**Methods:**

We used thirty-two AD-related cohorts, with genetic data from various high-density single-nucleotide polymorphism microarrays, whole-genome sequencing, and whole-exome sequencing. Study participants were filtered to be ages 60 and older, non-Hispanic, of European ancestry, and diagnosed as cognitively normal or AD (*n* = 65,701). Primary analyses investigated AD risk in APOE*4/4 carriers. Additional supporting analyses were performed in APOE*3/4 and 3/3 strata. Outcomes were compared under two different APOE*2/3/4 filtering approaches.

**Results:**

Using more conventional APOE*2/3/4 filtering criteria (approach 1), we showed that, when in-phase with APOE*4, rs439401 was variably associated with protective effects on AD case-control status. However, when applying a novel filter that increases the certainty of the APOE*2/3/4 genotypes by applying more stringent criteria for concordance between the provided *APOE* genotype and imputed *APOE* genotype (approach 2), we observed that all significant effects were lost.

**Conclusions:**

We showed that careful consideration of *APOE* genotype and appropriate sample filtering were crucial to robustly interrogate the role of the *APOE* locus on AD risk. Our study presents a novel *APOE* filtering approach and provides important guidelines for research into the *APOE* locus, as well as for elucidating genetic interaction effects with APOE*2/3/4.

**Supplementary Information:**

The online version contains supplementary material available at 10.1186/s13195-022-00962-4.

## Introduction

APOLIPOPROTEIN E*4 (APOE*4) is the strongest genetic risk factor for late-onset Alzheimer’s disease (AD) [1]. In subjects of European ancestry, one copy of APOE*4 increases the risk of a clinical diagnosis of AD by about 3-fold and two copies increase the risk by about 12-fold [2, 3]. APOE*2 on the other hand decreases the risk of AD by about half [3], while APOE*3 is the reference allele. Beyond the two common missense variants that compose APOE*2/3/4 (rs429358 and rs7412), there may be other coding variants on *APOE* or non-coding regulatory variants in the *APOE* locus that further impact AD risk, either independently or in conjunction with APOE*2/3/4 [4–15]. This pertains, by example, to a crucial question in the field: why do some APOE*4 carriers remain asymptomatic even into advanced old age? One possibility is that there may be genetic variants in the *APOE* locus that affect APOE*4 availability and in turn mitigate APOE*4-related risk for AD. Identifying such variants would importantly advance our understanding of APOE*4 pathophysiology and provide critical guidance for AD therapies aimed at APOE*4 [16, 17].

Despite its therapeutic promise and three active decades of research, the *APOE* locus remains relatively poorly understood in AD. While there are multiple reasons contributing to this, one prominent one is that the *APOE* locus harbors multiple nearby genes and shows a complex linkage disequilibrium (LD) structure with APOE*2/3/4, making it difficult to identify causal variants and interaction effects [18, 19]. Other important reasons are that relevant risk variants may be rare, thus requiring large sample sizes, and that the quality of the APOE*2/3/4 genotype can bear heavily on correctly identifying interaction effects and causal haplotypes. The latter may be of particular relevance given the plethora of available protein-based (e.g., two-dimensional gel electrophoresis and MALDI-TOF mass spectrometry) and DNA-based methods (e.g., TaqMan assays, high-resolution melting analysis, PCR sequencing, etc.) for APOE*2/3/4 genotyping [20–24]. Importantly, these methods have variable quality and limitations related to the haplotypic nature of APOE*2/3/4. For instance, protein-based assays may suffer from biases in detecting different *APOE* isoforms, while DNA-based assays can be affected by rare variants in the genomic region near APOE*2/3/4 (cf. Huang et al. for a detailed review) [25]. In turn, cohorts that are commonly included in genetic association studies of AD have used variable *APOE* genotyping methods [26–30], which has thus led to variable APOE*2/3/4 genotype quality across cohorts used in meta-analyses. The approach used to quality control the APOE*2/3/4 genotype is therefore critical to ensure robust association analyses. While the need for stringent *APOE* quality control is not necessarily novel, to our knowledge, there is currently no specific study that clearly addresses this issue, nor are there are any consensus guidelines.

In this study, we present analysis approaches and related findings to guide future research in the *APOE* locus. Specifically, we show findings for a large-scale analysis of rs439401 and its association with AD risk. This variant, located 1801 base pairs downstream of *APOE*, was recently identified as a brain *APOE* splice quantitative trait locus (sQTL) in GTEx [31, 32], spurring our interest to investigate it. We hypothesized it may affect APOE*4-related risk for AD and observed that it is most often seen on the same chromosome copy as APOE*3 (i.e., is in-phase with APOE*3), but in rare instances was seen together with APOE*4. We thus stratified analyses according to APOE*3 and APOE*4 genotypes to evaluate whether effects depended on the variant being in-phase with APOE*4. We use analyses on this variant to illustrate how critical it is to have accurate APOE*2/3/4 genotype data. Based on initial analyses using a conventional *APOE* filtering approach and a subsequent robustness assessment, we designed and present a novel *APOE* filtering approach that we believe will be highly relevant to help guide further reproducible research in this area.

## Methods

### Ascertainment of genotype and phenotype data

Genotype data for subjects with AD-related clinical outcome measures were available from thirty-two cohorts, incorporating three sequencing projects [33–56]. Across cohorts, genotyping was performed using various high-density single-nucleotide polymorphism (SNP) microarrays, whole-exome sequencing (WES), and whole-genome sequencing (WGS) (Table S1). The discovery samples comprised publicly available case-control (majority), family-based, population-based, and longitudinal cohorts. Independent replication samples, genotyped on SNP microarrays, were available from three large cohorts: the Rotterdam study, a population-based prospective study, the European Alzheimer Disease Initiative (EADI), roughly two-thirds of which is from a prospective population-based study and one third from case-control samples, and the European Alzheimer & Dementia BioBank (EADB), which collated AD case-control samples from 15 European countries. Ascertainment of genotype/phenotype data for each cohort/project are described in detail elsewhere [33, 40–44, 46, 47, 54]. Cross-sample genotype/phenotype harmonization for the discovery samples is summarized in Supplementary Methods. Phenotypes from respective cohorts were updated as of March 2021. Data were analyzed between December 2019 and June 2021.

### Genetic data quality control and processing

Genetic data in the discovery samples underwent standard quality control (QC; Plink v1.9) and ancestry determination (SNPweights v2.1; Fig. S1) [57]. Only non-Hispanic subjects of European ancestry (representing the vast majority of samples) were selected for processing. Data were restricted to those providing coverage of the rs439401 variant. Principal component analysis of genotyped SNPs provided principal components (PCs) capturing population substructure (PC-AiR, Fig. S2) [58]. Identity-by-descent (IBD) analyses reliably identified kinship down to 3rd degree relatedness (PC-Relate, Fig. S3) [58]. Sparse genetic relationship matrices (GRM) were constructed to enable analyses including related individuals [59]. SNP array data were used to perform genotype imputation with regard to the TOPMed imputation reference panel [60, 61]. Genetic processing of Rotterdam, EADI, and EADB replication samples is described elsewhere [33, 54]. Detailed descriptions of all processing steps are in Supplementary Methods and Table S2.

### Ascertainment of rs439401

The rs439401 variant was originally included in our analyses as it had a cross-cohort genotyping rate >80% in the discovery samples. Genotypes were considered from either the direct call on the SNP array data (i.e., called from probe intensity data) or the call from WGS data. We specifically relied solely on directly genotyped data rather than using imputed data in order to obtain unbiased results. This choice was additionally motivated reasoning that putative rare haplotypes may not be accurately imputed, particularly when using the commonly younger (non-AD) individuals in imputation reference panels [60, 62, 63]. Genotype reliability for the variant was verified by cross-correspondence across 3804 duplicate samples in the discovery and by assessing genotype intensity data on the SNP microarray in EADB.

### Ascertainment of *APOE* genotypes

Throughout, we will refer to APOE*2/3/4 genotypes as *APOE* genotypes. *APOE* genotypes were available from (1) cohort demographics (i.e., “provided” *APOE*), which generally had *APOE* genotype status determined through various direct genotyping methods (detailed elsewhere [33, 54]), (2) directly from WES/WGS calls, or (3) through imputation of rs429358 (which captures the APOE*4 allele) and rs7412 (which captures the APOE*2 allele). It is relevant to note that rs429358 was never directly available on the SNP microarrays. It is further relevant to note that for the current WES data from ADSP, rs7412 was not available, with only rs429358 being reliably called in most subjects. The WES data could thus be used only to verify subjects with a provided APOE*3/3, 3/4, or 4/4 status (cf. Supplementary Methods).

### *APOE* genotype filtering criteria

To our understanding, common criteria across prior studies regarding *APOE* genotypes can be summarized as giving priority to provided *APOE* genotypes when available (as direct genotyping methods are generally considered the gold standard), followed by using *APOE* genotypes derived from rs429358 and rs7412 when directly called on a SNP microarray, followed by inference of *APOE* genotypes through (high quality) imputation of rs429358 and rs7412. There is no clear consensus on whether or how any discrepancies across available *APOE* genotypes for a given subject should be adjudicated. Furthermore, with the recent increasing availability of WGS/WES data in the AD field [42, 46, 51], these data can now also be used to verify *APOE* genotypes. When high-quality WGS/WES calls are available for rs429358 and rs7412 (i.e., good read depth/quality with a clear reference/alternate allele distribution) [64], the derived *APOE* genotype may be considered the ground truth. Recent work indeed suggests that a higher *APOE* genotype accuracy can be achieved using next-generation sequencing compared to conventional gold standard methods [65].

#### *APOE* filtering approach 1

Based on the above considerations, we designed criteria to use *APOE* genotypes according to the highest available quality. Specifically, when multiple *APOE* genotypes were available for a given subject, the *APOE* genotype we selected followed the priority of WGS/WES over provided/demographic sources (for details regarding “provided/demographic” *APOE* sources, please cf. above in the section “Ascertainment of *APOE* genotypes”). If *APOE* genotype was only available from provided/demographic sources and was discordant across duplicate samples, then those samples were flagged for exclusion (*N* = 73 out of 1501 (4.86%) unique subjects). Similarly, the correspondence between *APOE* genotypes derived from WES and WGS across duplicate samples was checked and only showed discordance in five subjects differing for APOE*2/3 and APOE*3/3 genotypes across the WES and WGS data (these subjects were excluded). The final set of samples used for association analyses thus did not display any mismatches in prioritized *APOE* genotypes across duplicates, but in some instances, the *APOE* genotype from provided/demographic versus WES/WGS sources differed. *APOE* status as inferred from imputation was entirely ignored, reasoning this was less reliable and that rare haplotypes of potential interest in the *APOE* locus may lead to false imputation of APOE*2/3/4 genotype.

#### *APOE* filtering approach 2

After further assessment of the initial results, we had concerns about the reliability of *APOE* genotype status in some APOE*4 subjects carrying rs439401 (cf. Results). We therefore expanded the first approach to exclude any subjects who had their prioritized *APOE* genotype determined from provided/demographic *APOE* but were still discordant with their imputed *APOE* genotype (*N* = 632 out of 12,753 (4.96%) in the discovery sample after passing all other filtering steps). Note that imputation scores (*R*^2^) for rs429358 and rs7412 were never lower than 0.8. Information regarding *APOE* imputation, as well as several correspondence checks across different sources of *APOE* genotypes, are provided in the supplementary and referenced in the “Results” section. An additional check for *APOE* genotype consistency was also performed using newly released sequencing data from the ADSP (NG00067.v5) [66], processed in May 2021 (cf. Supplementary Methods and the “Results” section).

**Fig. 1:**
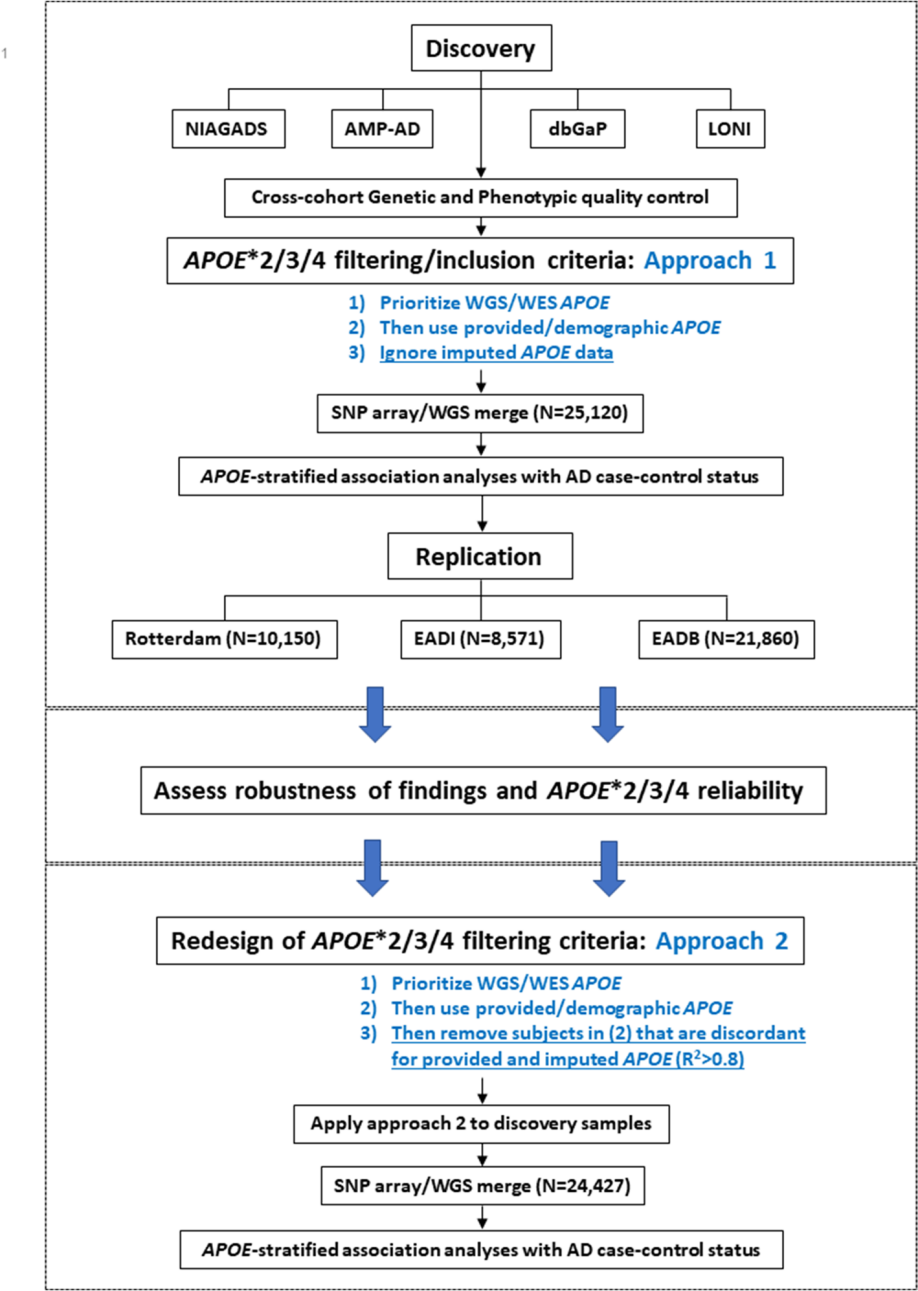
Schematic overview of the study design and two APOE*2/3/4 filtering approaches

An overview of the study design and *APOE* filtering approaches is presented in Fig. 1.

### Simulations of concordance rates between observed and true APOE*4/4 genotypes

In order to understand potential uncertainty in APOE*4/4 genotypes, we simulated different type I and II error rates for APOE*4/4 status. Type I error rate was defined as the probability, p1, to mis-classify non-APOE*4/4 carriers as APOE*4/4. Type II error rate was defined as the probability, p2, to mis-classify APOE*4/4 carriers as non-APOE*4/4. We considered a range of true frequencies, f_true_, for APOE*4/4 cases and controls respectively with regard to all cases and controls (that is, all *APOE* strata). This range for f_true_ was centered on observations in the current discovery samples, which should represent a reasonable approximation of expected frequencies in case-control samples. The observed frequency, f_obs_, was then defined as f_true_*(1-p2)+(1-f_true_)*p1. The concordance rate between observed and true APOE*4/4 was finally defined as f_true_*(1-p2)/f_obs_.

### Statistical analyses

Primary analyses evaluated associations of rs439401 with relative risk for AD in APOE*4/4 carriers using additive genetic models. In additional supporting analyses, associations were evaluated in APOE*3/4 carriers, comparing wild-type (WT) to homozygote (HOM) genotypes, ensuring rs439401 was in-phase with APOE*4. The expectation here was to observe similar but attenuated effects compared to associations in APOE*4/4 carriers. Additional associations were evaluated in APOE*3/4 and 3/3 carriers using additive genetic models, with the expectation of observing little or no effect if associations were conditional on being in-phase with APOE*4. APOE*2/4 carriers were not considered given sample paucity. Analyses were restricted to subjects aged 60 and above, consistent with age cutoffs in prior genetic studies of AD [54]. Replication analyses focused only on evaluating variants in-phase with APOE*4. Lastly, to provide additional insight into the putative role of rs439401 in AD, we evaluated the association of rs439401 with relative risk for AD in the full discovery sample, while adjusting for APOE*2 and APOE*4 dosage.

Cohorts in the discovery were combined into a single mega-analysis, included related subjects, and outcome measures were adjusted for age, sex, the first five genetic PCs, and the GRM. In full sample analyses, models further included APOE*2 and APOE*4 dosage as covariates. In replications, models included only unrelated subjects and were not adjusted for the GRM. EADI and Rotterdam further adjusted for the first three genetic PCs, while EADB adjusted for the first 20 genetic PCs and genotyping center. Notably, models in the discovery mega-analyses did not adjust for cohort, reasoning that this may inadvertently diminish power given variable cohort sizes and carrier distributions. This is especially relevant in case of lower frequency variants in the APOE*4/4 stratum, where cohort bins and the number of allele observations become very small. Still, to address potential concerns regarding cohort biases, in sensitivity analyses, the effect of cohort adjustment in the discovery was evaluated.

Associations with AD risk were evaluated under a case-control design using linear mixed-model regression in analyses of related subjects and logistic regression in analyses of unrelated subjects. Additional details for model/inclusion criteria are in Supplementary Methods. Association analyses were considered significant below a threshold *P*-value of 0.05. All analyses were performed in R v3.6.0.

## Results

### Participant demographics and rs439401 linkage structure

Across all 142,075 genotyped samples considered in this study (Table S1), 65,701 unique participants passed filtering and inclusion criteria. Participant demographics for APOE*4/4 and 3/4 carriers are in Table 1, while detailed full sample demographics are in Table S3-4. In the discovery, rs439401 displayed high LD (D’>0.9) with APOE*3, but in rarer instances was observed in-phase with APOE*4, thereby deviating from the expected LD structure (Table S5).

**Table 1:** Sample demographics for association analyses with Alzheimer’s disease case-control status

Cohort				APOE*3/4 carriers			APOE*4/4 carriers		
Name	Participants after QC (N)	Diagnosis (N)		(N (%))	Female (N (%))	Age (Mean (SD))	(N (%))	Female (N (%))	Age (Mean (SD))
Discovery	25120	CN	12340	2707 (21.9 %)	1615 (59.7 %)	76.2 (8.4)	238 (1.9 %)	145 (60.9 %)	73.7 (7.4)
		AD	12780	5740 (44.9 %)	3411 (59.4 %)	73.8 (6.9)	1652 (12.9 %)	895 (54.2 %)	70.1 (6.2)
Replication - Rotterdam	10150	CN	8824	1868 (21.2 %)	1034 (55.4 %)	76.4 (9.3)	150 (1.7 %)	74 (49.3 %)	73.8 (8.0)
		AD	1326	411 (31.0 %)	282 (68.6 %)	82.2 (6.4)	86 (6.5 %)	50 (58.1 %)	78.2 (6.9)
Replication - EADI	8571	CN	6502	1141 (17.5 %)	672 (58.9 %)	79.6 (6.4)	62 (1.0 %)	45 (72.6 %)	77.8 (6.4)
		AD	2069	801 (38.7 %)	529 (66.0 %)	73.9 (7.4)	205 (9.9 %)	134 (65.4 %)	69.4 (5.9)
Replication - EADB	21860	CN	12295	2498 (20.3 %)	1431 (57.3 %)	72.4 (7.9)	195 (1.6 %)	107 (54.9 %)	70.3 (7.5)
		AD	9565	3912 (40.9 %)	2522 (64.5 %)	74.0 (7.2)	959 (10.0 %)	551 (57.5 %)	70.4 (6.8)

Cohort data were available through the National Institute on Aging and Genetics of Alzheimer’s Disease Data Storage Site (NIAGADS), the National Alzheimer’s Coordinating Center (NACC), Accelerating Medicines Partnership – Alzheimer’s Disease (AMP-AD) Knowledge Portal, the Database of Genotypes and Phenotypes (dbGaP), Rush Alzheimer’s Disease Center at Rush University, the Image & Data Archive powered by Laboratory of Neuro Imaging (IDA-LONI), the Rotterdam study, the European Alzheimer’s Disease Initiative (EADI), and the European Alzheimer & Dementia BioBank (EADB). Cohorts included Adult Changes in Thought (ACT), Alzheimer’s Disease Center Datasets (ADC1-7) for which phenotype data is managed by NACC, European collaboration for the discovery of novel biomarkers for Alzheimer’s disease (ADDNEURO), the Alzheimer’s Disease Neuroimaging Initiative (ADNI), ADNI Department of Defense (ADOD), Alzheimer’s Disease Sequencing Project (ADSP) Discovery and Extension Phase, National Institute on Aging Genetics Initiative for Late-Onset Alzheimer’s Disease (NIA-LOAD), Oregon Health and Science University study (OHSU), Mayo Clinic Alzheimer’s Disease Genetics studies (MAYO), MAYO RNAseq study (MAYO2), Multi Institutional Research on Alzheimer Genetics Epidemiology (MIRAGE), Mount Sinai Brain Bank (MSBB), University of Miami/Texas Alzheimer’s Research Care Consortium Wave 2/Case Western Reserve University (MTC), Rush University Religious Orders Study/Memory and Aging Project (ROSMAP), Translational Genomics Research Institute series 2 (TGEN2), University of Miami/Vanderbilt University/Mt. Sinai School of Medicine studies (UM/VU/MSSM), University of Pittsburgh study (UPITT), the Washington University study (WASHU), Washington Heights-Inwood Community Aging Project (WHICAP), the Rotterdam study, the European Alzheimer’s Disease Initiative (EADI), and the European Alzheimer & Dementia BioBank (EADB)

*Abbreviations:* *CN* cognitively normal, *AD* Alzheimer’s disease, *QC* quality control, *SD* standard deviation, *SNP* single nucleotide polymorphism

### *APOE* filtering approach 1: Rs439401 shows variable association with Alzheimer’s disease risk

Primary case-control findings in APOE*4/4 carriers in the discovery showed that rs439401 displayed a strong, protective, and significant effect on case-control status (Table 2). It displayed similar protective effect sizes in EADI and Rotterdam replication samples, but was risk increasing in EADB, and did not reach significance in any replication sample. When in-phase with APOE*4 in APOE*3/4 (WT-HOM) stratified analyses, rs439401 showed a protective significant effect in the discovery, but variable non-significant results in the replication samples (Table 2). In contrast, in the discovery, rs439401 did not associate with AD risk in APOE*3/4 (additive model) or 3/3 stratified analyses (Table S6), nor in the full sample analysis (odds ratio = 0.99; 95% confidence interval = [0.95, 1.03], *P*-value = 0.61).

**Table 2:** Results from *APOE* filtering approach 1: association findings for rs439401, when in-phase with APOE*4, with Alzheimer’s disease case-control status

	Genotype distributions			AD Case-Control regression	
Group/model	CN, carrier No. / Total No. (%)	AD, carrier No. / Total No. (%)	CN - AD, MAF (%)	OR (95% CI)	*P*-value
rs439401 - T allele tested					
APOE*4/4 - additive model					
Discovery	14 / 237 (5.91 %)	19 / 1652 (1.15 %)	3.59 % - 0.64 %	0.10 (0.04, 0.24)	1.64E-07
Rotterdam	5 / 150 (3.33 %)	2 / 86 (2.33 %)	2.33 % - 1.16 %	0.43 (0.10, 1.80)	0.25
EADI	1 / 62 (1.61 %)	2 / 205 (0.98 %)	0.80 % - 0.49 %	0.37 (0.01, 12.7)	0.58
EADB	2 / 195 (1.03 %)	21 / 956 (2.20 %)	0.52 % - 1.20 %	1.61 (0.39, 6.77)	0.51
APOE*3/4 - WT vs HOM					
Discovery	19 / 1401 (1.36 %)	14 / 2974 (0.47 %)	-	0.55 (0.38, 0.80)	1.58E-03
Rotterdam	8 / 993 (0.81 %)	3 / 220 (1.36 %)	-	1.21 (0.31, 4.72)	0.78
EADI	4 / 593 (0.67 %)	5 / 420 (1.19 %)	-	1.22 (0.60, 2.49)	0.58
EADB	12 / 1343 (0.89 %)	21 / 2070 (1.01 %)	-	0.80 (0.55, 1.17)	0.25

*Abbreviations:* *CN* cognitively normal, *AD* Alzheimer’s disease, *OR* odds ratio, *CI* confidence interval

Because of the use of a mega-analysis design that does not adjust for cohort, there may still be concern for potential cohort biases. Therefore, as a sensitivity analysis, we re-evaluated the case-control discovery findings, now adjusting for cohort or cohort/array/center (Fig. S5). These analyses indicated diminished significances, but effect sizes remained comparable and rs439401 remained strongly significant in APOE*4/4 carriers.

### Robustness assessment: limitations to *APOE* filtering approach 1

After the initial analyses, we assessed the robustness of the primary discovery findings. This appeared particularly relevant considering the very low frequency of rs439401 carriers in APOE*4/4 controls in EADB versus other cohorts, suggesting potential biases in the controls across the cohorts.

The concordance rate of rs439401 from duplicate samples across microarrays and WGS (99.97%) supported genotype reliability (Table S7). Similarly, the variant appeared confidently called from the EADB microarray intensity data (Fig. S4). Overall, we concluded there were no specific genotyping issues for rs439401.

**Fig. 2:**
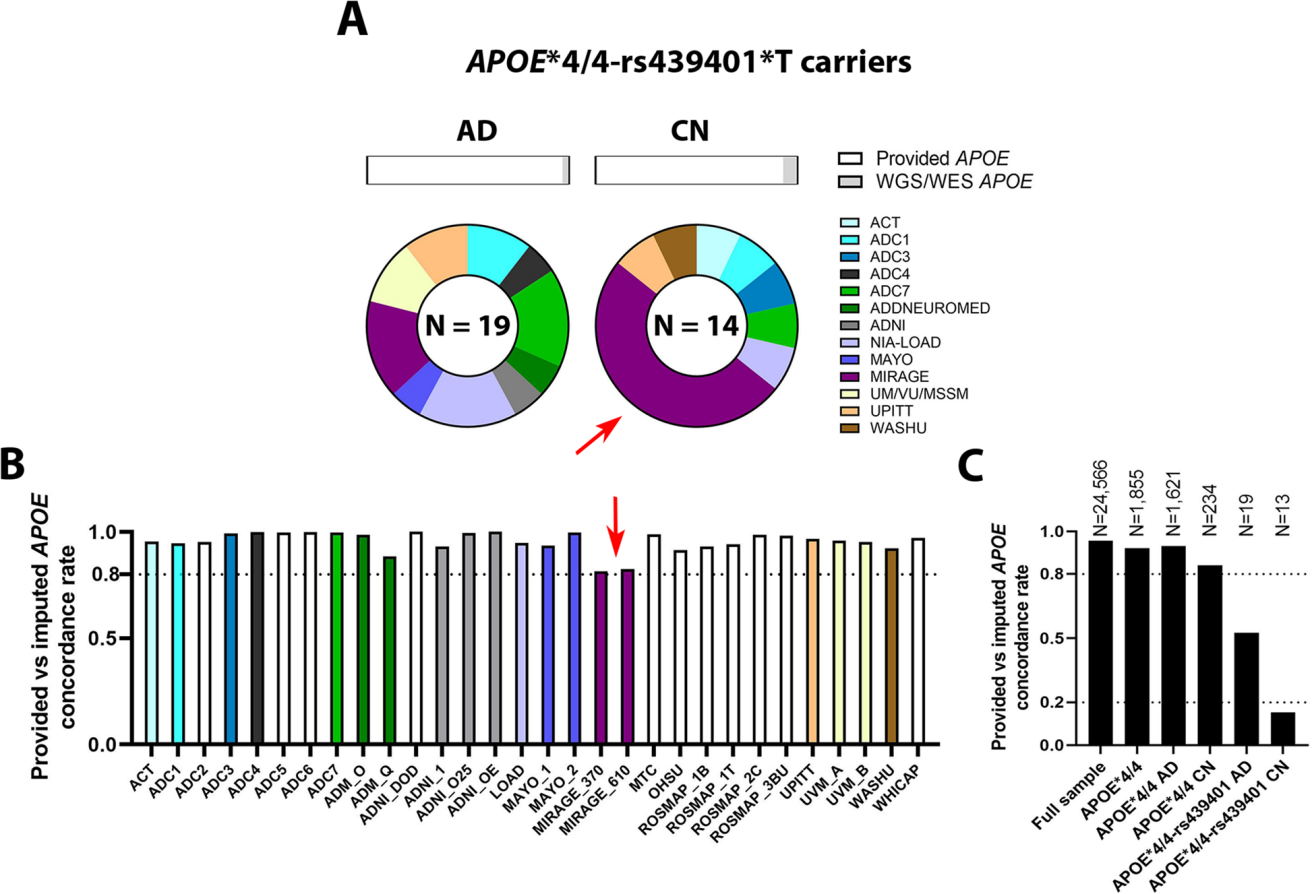
Limitations in *APOE* filtering approach 1 are reflected in discordance between imputed and provided *APOE* genotypes, particularly in APOE*4/4 carriers. **A** APOE*4/4-rs439401 carrier cohort distributions. The top section shows the distribution of prioritized *APOE* genotype source in approach 1, indicating that APOE*4/4 carriers of rs439401 had very few WGS/WES-verified APOE*4/4 data. The bottom section shows pie charts for carrier distributions across cohorts (Additional data in Table S8). The red arrow indicates that a large fraction of control rs439401 carriers was contributed by MIRAGE. **B** Concordance rates between provided and imputed *APOE* per cohort (additional data in Table S9). The red arrow indicates that MIRAGE had the lowest concordance rate, suggesting potential limitations with its provided *APOE* data that could explain observations in **A**. **C** Concordance rates between provided and imputed *APOE* for the discovery sample, considering multiple strata (additional data in Table S10). APOE*4/4 strata considered provided APOE*4/4 genotypes after applying *APOE* filtering approach 1. Note decreased concordance in APOE*4/4 controls compared to cases. Note strongly decreased concordance for rs439401 carriers, specifically controls. Simulations confirmed that APOE*4/4 controls are more likely than cases to not actually be APOE*4/4 carriers (cf. Fig. S6-7). Abbreviations: *CN*, cognitively normal; *AD*, Alzheimer’s disease; *OR*

Another important consideration is that some error rate is expected for the different direct *APOE* genotyping methods used across cohorts. Overall, the reliability of the APOE*4 genotype may thus be of concern especially when considering the rare APOE*4-rs439401 haplotype. After assessing all APOE*4/4-rs439401 carriers, it was apparent that one cohort, MIRAGE, contributed a large amount of APOE*4/4-rs439401 controls for which *APOE* status was available only from provided/demographic sources (Fig. 2A, Table S8). We then assessed the concordance rate between provided and imputed *APOE* genotypes across all respective cohorts and observed that MIRAGE displayed the lowest concordance rate of all cohorts included in the discovery analyses (Fig. 2B), despite comparably high imputation scores for rs429358 and rs7412 to other cohorts (Table S9). Overall, this supported concern for the APOE*4/4-rs439401 controls from MIRAGE.

Extending on the above considerations, we assessed discordance rates between imputed and provided *APOE* for different strata (Fig. 2C, Table S10). Importantly, while the discordance rate was only 4.3% in the full sample, it increased to 7.2% in APOE*4/4 cases, further increased to 16.1% in APOE*4/4 controls, and then drastically increased to 47.4% in APOE*4/4-rs439401 carrier cases and 85.7% in APOE*4/4-rs439401 carrier controls. While our a priori assumption for approach 1 reasoned that imputed *APOE* may be discordant with provided *APOE* in case of subjects with rare haplotypes (e.g., APOE*4/4-rs439401 carriers), the observation that this discordance was 2-fold higher in controls compared to cases would not be expected. Rather, it more likely indicates that a miscall of the *APOE* genotype was true in at least some of these individuals. To better understand these observations, we performed simulation studies using different type I and type II error rates (0–5%) for APOE*4/4 genotyping and observed that APOE*4/4 controls were more likely than APOE*4/4 cases to not actually be APOE*4/4 carriers (Fig. S6-7). This was the result of the low frequency of APOE*4/4 controls and the strong case-control imbalance in APOE*4/4 carriers. Overall, this supported concern for the validity of approach 1.

We then used the recently released new ADSP WGS and WES data, which now cover additional subjects that are duplicated on SNP array samples included in our discovery analyses (*N* = 3644 as determined by identity-by-descent). We assessed the *APOE* genotype calls from the novel WES/WGS data and observed that three APOE*4/4-rs439401 control subjects (not from the MIRAGE cohort) in the prior discovery samples were in fact APOE*3/3 or APOE*3/4 carriers, which was also the imputed *APOE* genotype (Table S8). Overall, this again raised concern about the validity of approach 1.

In sum, these additional checks for robustness of the findings suggested problems with *APOE* genotype reliability in subjects with APOE*4-rs439401 haplotypes and APOE*4/4 carriers overall, indicating a limitation to the first (conventional) *APOE* filtering approach. In a final check, we observed that despite good concordance between provided and WGS *APOE* (99.1%), imputed and WGS *APOE* was more concordant (97.2%) than imputed and provided *APOE* (95.7%), indicating that at least in some subjects imputed *APOE* was likely more correct than provided *APOE* (Table S10).

### *APOE* filtering approach 2: Rs439401 shows no association with Alzheimer’s disease risk

**Fig. 3:**
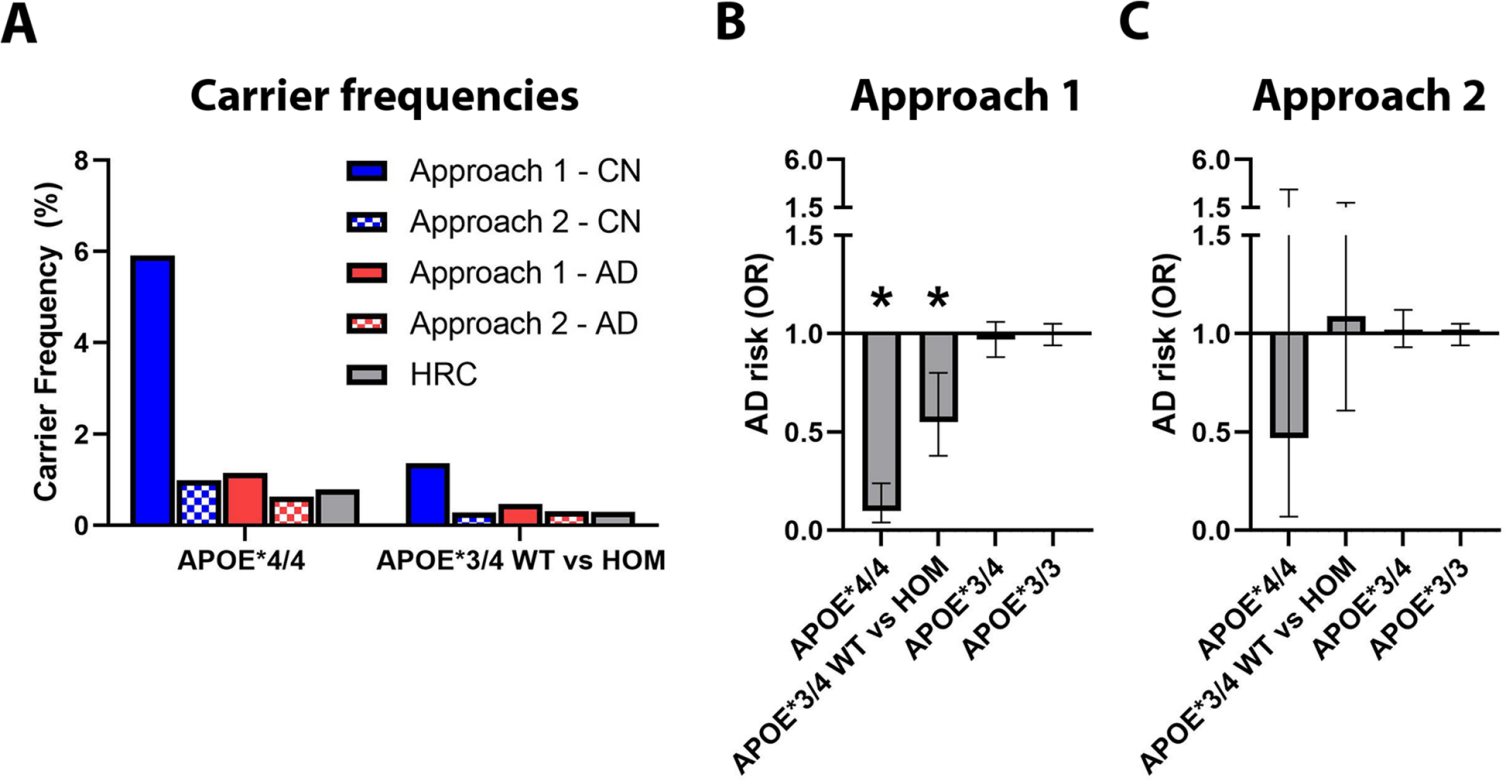
Overview of rs439401 frequencies and case-control association findings, comparing *APOE* filtering approach 1 to approach 2. **A** Carrier frequencies across both approaches for APOE*4/4 and APOE*3/4 WT vs HOM groups, as well as in the Haplotype reference consortium v1.1 (HRC). Note decreased frequencies for rs439401 in approach 2 that appear concordant with the HRC. **B**, **C** Overview of association findings for all evaluated strata, comparing **B** approach 1 to **C** approach 2. Significant effects are denoted by an asterisk (*). Error bars show 95% confidence intervals. Note loss of significant effects in approach 2

In light of the identified *APOE* reliability limitations, we extended approach 1 to filter out any subjects that did not have WGS/WES *APOE* and at the same time were discordant for provided and (high-quality) imputed *APOE.* We also filtered out any discordant *APOE* calls with regard to the new ADSP WES/WGS data since this information was available (in case of APOE*4-rs439401 carriers, this overlapped with samples where provided and imputed *APOE* were discordant). We then applied this to the discovery samples and reran analyses. Exclusion of subjects with discordant *APOE* status with the newly released ASDP WES/WGS data removed 61 (out of 12,367) subjects from the SNP-array samples. Further applying the new *APOE* filter excluded 632 (out of 12,753 considered) subjects from the discovery SNP-array samples. APOE*4-rs439401 carrier frequencies dropped substantially, particularly in controls, and became more consistent with those observed in the haplotype reference consortium (Fig. 3A). Case-control association analyses now indicated no effects for APOE*4-rs439401 carriers (Table 3 and Fig. 3B, C) and still no effect in full sample analyses (odds ratio = 1.00; 95% confidence interval = [0.96, 1.05], *P*-value = 0.93). In sum, approach 2 produced results that were more realistic in terms of expected linkage structure and more consistent with the lack of significant replication findings.

**Table 3:** Results from *APOE* filtering approach 1 versus 2 in the discovery: association findings for rs439401, when in-phase with APOE*4, with Alzheimer’s disease case-control status

	Genotype distributions			AD Case-Control regression	
Group/model	CN, carrier No. / Total No. (%)	AD, carrier No. / Total No. (%)	CN - AD, MAF (%)	OR (95% CI)	*P*-value
rs439401 - T allele tested					
APOE*4/4 - additive model					
Discovery – approach 1	14 / 237 (5.91 %)	19 / 1652 (1.15 %)	3.59 % - 0.64 %	0.10 (0.04, 0.24)	1.64E-07
Discovery – approach 2	2 / 203 (0.99 %)	10 / 1577 (0.63 %)	0.49 % - 0.32 %	0.47 (0.07, 3.21)	0.44
APOE*3/4 - WT vs HOM					
Discovery – approach 1	19 / 1401 (1.36 %)	14 / 2974 (0.47 %)	-	0.55 (0.38, 0.80)	1.58E-03
Discovery – approach 2	4 / 1339 (0.29 %)	9 / 2914 (0.31 %)	-	1.09 (0.61, 1.94)	0.78

*Abbreviations: CN* cognitively normal, *AD* Alzheimer’s disease, *OR* odds ratio, *CI* confidence interval

## Discussion

Our results demonstrate that the filtering criteria for APOE*2/3/4 genotypes can heavily impact association finding for variants that exert their effect in conjunction with APOE*2/3/4. Specifically, we used the *APOE* sQTL variant rs439401 to illustrate this point. Using more conventional filtering criteria regarding *APOE* genotypes (approach 1), we showed that, when in-phase with APOE*4, rs439401 was variably associated with protective effects on AD case-control status. However, when assessing the reliability of APOE*2/3/4 genotypes with more scrutiny and applying a novel filter to increase certainty of the *APOE* genotypes (approach 2), we observed that all significant effects were lost. The findings and methodology presented here are thus of high relevance to guide future research into the *APOE* locus. Specifically, we propose that our approach 2 can serve as a consensus *APOE* genotyping approach for future studies, namely, to prioritize first WGS/WES APOE*2/3/4 genotypes if available (and if only either rs429358 or rs7412 is available from WGS/WES, to use those genotype data to verify the provided/demographic APOE*2/3/4 genotypes); second to use provided/demographic APOE*2/3/4 genotypes; and third, in subjects without WGS/WES information, to exclude those for whom the provided/demographic and imputed (R2>0.8) APOE*2/3/4 genotypes are discordant. Another important step to ensure the highest quality of APOE*2/3/4 genotypes is to verify and harmonize this information across available duplicate samples.

The rs439401 variant considered in the current study has previously been investigated with regard to AD risk in different contexts and using variable strategies and study designs [8–11, 13]. Our analyses however considered a substantially larger sample size, essentially incorporating most European ancestry AD cohorts included in prior studies, specifically focused on evaluating effects stratified to respective *APOE* genotypes, and tested only directly genotyped variants. Further, up-to-date genotype and phenotype data for a large set of AD cohorts was jointly harmonized to compose a parsimonious discovery sample. Non-European ancestries were not investigated here owing to the paucity of publicly available data. When compared to similar prior studies [6, 13–15], our discovery group was larger and we incorporated three large replication cohorts. Furthermore, through the implementation of linear mixed modeling and cross-sample harmonization, we were able to increase the power and specificity for variant discovery, while additionally verifying genotype reliability across nearly 4000 duplicate samples. In sum, our analyses should provide a robust assessment of the presented *APOE* filtering approaches and rs439401’s association with AD risk.

A recent study, using samples largely overlapping with the current discovery (but smaller in size) and an *APOE* filtering approach similar to our approach 1, evaluated the association of variants on the larger *APOE* locus with AD risk in APOE*4/4 carriers and did not identify the strong association of rs439401 that we observed in approach 1 [13]. Beyond differences in sample size and harmonization, the latter study adjusted models by study/cohort and made use of imputed genotypes. We specifically decided in primary analyses not to adjust for cohort, as we reasoned that this may inadvertently diminish power given variable cohort sizes and carrier distributions, especially in APOE*4/4 carriers. We further reasoned that through our extensive phenotype/genotype harmonization and the use of a mixed model mega-analysis design, which may capture some latent cohort effects, there was less concern for potential cohort bias. Additionally, given the complex LD structure of the *APOE* locus, we were concerned about the reliability of imputation and focused only on directly called genotypes. A similar limitation regarding imputation was recognized by the authors of the prior study [13]. These differences likely explain why rs439401 was not observed in their study. Regardless of our considerations and of cohort adjustment, we determined that the *APOE* filtering criteria were the most relevant factor for variable rs439401 association findings.

One important insight from our study was that subjects, particularly controls, with a provided APOE*4/4 genotype had a higher probability of discordance between their imputed and provided genotype than did subjects in the full sample. Such biases are, however, not limited to APOE*4/4 carriers. The six *APOE* genotypes (*2/2, 2/3, 3/3, 2/4, 3/4, 4/4) show large differences in numbers of carriers and case-control ratios, owing to the allele frequencies of rs429358/rs7412 and their effect on AD risk. As a result, the different *APOE* genotypes will be expected to have different concordance rates between true and observed *APOE* genotypes. We observed varying concordance between imputed and provided *APOE* across the six *APOE* genotypes, with particularly lower concordance rates in APOE*2 carriers (Fig. S8). Just as the APOE*4/4 provided genotype was most likely to be incorrect here in controls (a phenotype for which APOE*4/4 is a particularly rare genotype), the APOE*2/2 genotype is more likely to be incorrect in cases (a phenotype for which APOE*2/2 is a particularly rare genotype). The proposed *APOE* genotype filter will therefore also be specifically relevant for studies focusing on APOE*2.

Our study highlights several important considerations for further work on the *APOE* locus. Most notably, we illustrate how *APOE* genotype filtering criteria can strongly impact association findings for variants in the *APOE* locus, especially when studying haplotypes or interaction effects with APOE*2/3/4. The same will hold true when considering non-local variants in, for instance, a genome-wide association study of AD in APOE*4/4 subjects, or when aiming to disentangle genetic interaction effects with APOE*2/3/4. Based on our observations, we suggest that future studies consider implementing the methodology that we proposed in approach 2 and subject their assessment of *APOE* genotypes to extensive scrutiny. The limitations observed for APOE*2/3/4 genotype reliability also emphasize that next-generation sequencing data will be crucial to interrogate the *APOE* locus with higher confidence and to ensure that putative rare haplotypes are not missed because of the need for sample filtering in SNP array data. Lastly, in order to have higher confidence in local haplotypes, long read sequencing approaches will additionally be crucial to help disentangle the local haplotype structure on *APOE* with regard to AD.

### Limitations

One limitation of our proposed approach is that it relies on the availability of high-quality imputed genotypes for rs429358 and rs7412, as well as careful phenotype/genotype harmonization across multiple data sources, which may not always be feasible for different research groups. Nonetheless, our findings show that efforts to increase APOE*2/3/4 genotype reliability should be pursued and that collaborative large-scale AD harmonization initiatives should consider this as an important focus. Furthermore, our approach may be considered to be highly conservative when excluding subjects for which the imputed and provided APOE*2/3/4 genotypes are discordant, since some of the imputed APOE*2/3/4 genotypes may in fact be the correct ones. Future studies may thus also consider retaining those subjects, using their imputed APOE*2/3/4 genotypes. Lastly, we propose to prioritize WES/WGS APOE*2/3/4 genotypes given the high quality and reliability of these sequencing technologies. However, as detailed in the supplement, careful consideration of genotyping quality and depth, integrated with provided APOE*2/3/4 genotype information, were crucial to maximize APOE*2/3/4 genotype reliability. It will therefore be critical that such information is made readily available and evaluated in future studies.

## Conclusion

We showed that careful consideration of *APOE* genotype and appropriate sample filtering was crucial to robustly interrogate the role of the *APOE* locus on AD risk. Our study presents a novel *APOE* filtering approach and provides important guidelines for research in this area, as well as for elucidating genetic interaction effects with APOE*2/3/4.

## Supplementary Information

**Additional file 1.** Supplementary material.

## Data Availability

All data used in the discovery analyses are available upon application to: - dbGaP (https://www.ncbi.nlm.nih.gov/gap/) - NIAGADS (https://www.niagads.org/) - LONI (https://ida.loni.usc.edu/) - Synapse (https://www.synapse.org/) - Rush (https://www.radc.rush.edu/) - NACC (https://naccdata.org/) The specific data repository and identifier for each cohort are indicated in Table S1 of the supplement. Because of ethical and legal restrictions, the Rotterdam, EADI, and EADB cohort data are available only upon request. Interested authors may respectively contact Dr. Ikram, at m.a.ikram@erasmusmc.nl, and Dr. Lambert, at jean-charles.lambert@pasteur-lille.fr.
