# Supplementary material for: Challenges at the APOE locus: a robust quality control approach for accurate APOE genotyping
Source: Alzheimers Res Ther. 2022 Feb 4;14:22. doi: 10.1186/s13195-022-00962-4 (PMC8815198; doi:10.1186/s13195-022-00962-4)
Supplement: Supplementary file 1 — Additional file 1. Supplementary material. [file 13195_2022_962_MOESM1_ESM.pdf]

Challenges at the *APOE* locus: A robust quality control approach for accurate *APOE* genotyping

Belloy et al.

Supplementary Material

Table of Contents

Consortia Author Lists 2
Supplemental Methods 13
Phenotype Ascertainment 13
Genetic Data Quality Control and Processing 17
Statistical Analyses – Additional Model Criteria 20
Supplemental Figures 21
Figure S1 21
Figure S2 22
Figure S3 25
Figure S4 26
Figure S5 27
Figure S6 28
Figure S7 29
Figure S8 30
Supplemental Tables 31
Table S1 31
Table S2 33
Table S3 34
Table S4 36
Table S5 38
Table S6 39
Table S7 40
Table S8 41
Table S9 42
Table S10 43
References 44

Consortia Author Lists

EADB

Céline Bellenguez[1], Fahri Küçükali[2,3,4], Iris Jansen[5,6], Victor Andrade[7,8], Sonia Moreno-Grau[9,10], Najaf Amin[11,12], Benjamin Grenier-Boley[1], Rafael Campos-Martin[7], Peter A. Holmans[13], Anne Boland[14], Luca Kleineidam[7,8,15], Vincent Damotte[1], Sven J. van der Lee[5,16], Teemu Kuulasmaa[17], Itziar de Rojas[9,10], Amber Yaqub[11], Ivana Prokic[11], Marcos R, Costa[1,18], Julien Chapuis[1], Shahzad Ahmad[11,19], Vilmantas Giedraitis[20], Dag Aarsland[21,22], Pablo Garcia-Gonzalez[9,10], Carla Abdelnour[9,10], Emilio Alarcón-Martín[9,23], Daniel Alcolea[10,24], Montserrat Alegret[9,10], Ignacio Alvarez[25,26], Victoria Álvarez[27,28], Nicola J. Armstrong[29], Tsolaki Anthoula[30,31], Ildebrando Appollonio[32,33], Marina Arcaro[34], Silvana Archetti[35], Alfonso Arias Pastor[36,37], Beatrice Arosio[38,39], Lavinia Athanasiu[40], Henri Bailly[41], Nerisa Banaj[42], Miquel Baquero[43], Ana Belén Pastor[44], Luisa Benussi[45], Claudine Berr[46], Céline Besse[14], Valentina Bessi[47,48], Giuliano Binetti[45,49], Alessandra Bizarro[50], Rafael Blesa[10,24], Mercè Boada[9,10], Barbara Borroni[51], Silvia Boschi[52], Paola Bossù[53], Geir Bråthen[54,55], Catherine Bresner[13], Henry Brodaty[29,56], Keeley J. Brookes[57], Luis Ignacio Brusco[58,59,60], Dolores Buiza-Rueda[10,61], Katharina Bûrger[62,63], Vanessa Burholt[64,65], Miguel Calero[10,44,66], Geneviève Chene[67,68], Ángel Carracedo[69,70], Roberta Cecchetti[71], Laura Cervera-Carles[10,24], Camille Charbonnier[72], Caterina Chillotti[73], Simona Ciccone[39], Jurgen A.H.R. Claassen[74], Jordi Clarimon[10,24], Christopher Clark[75], Elisa Conti[32], Anaïs Corma-Gómez[76], Emanuele Costantini[77], Carlo Custodero[78], Delphine Daian[14], Maria Carolina Dalmasso[7], Antonio Daniele[77], Efthimios Dardiotis[79], Jean-François Dartigues[80], Peter Paul de Deyn[81], Stéphanie Debette[80,82], Jürgen Deckert[83], Teodoro del Ser[44], Nicola Denning[84], Martin Dichgans[62,63,85], Janine Diehl-Schmid[86], Mónica Diez-Fairen[25,26], Paolo Dionigi Rossi[39], Srdjan Djurovic[40], Emmanuelle Duron[41], Emrah Düzel[87,88], Carole Dufouil[67,68], Valentina Escott-Price[13,84], Ana Espinosa[9,10], Michael Ewers[62,63], Marta Fernández-Fuertes[76], Catarina B Ferreira[89], Evelyn Ferri[39], Bertrand Fin[14], Peter Fischer[90], Tormod Fladby[91], Klaus Fließbach[8,15], Juan Fortea[10,24], Silvia Fostinelli[45], Nick C. Fox[92], Emlio Franco-Macías[93], María J. Bullido[10,94,95], Ana Frank-García[10,94,96], Lutz Froelich[97], Daniela Galimberti[34,88], Jose Maria García-Alberca[10,98], Pablo García-González[9], Sebastian Garcia-Madrona[99], Guillermo Garcia-Ribas[99], Roberta Ghidoni[45], Ina Giegling[100], Giaccone Giorgio[85], Oliver Goldhardt[86], Antonio González-Pérez[101], Caroline Graff[102,118], Giulia Grande[103], Emma Green[104], Timo Grimmer[86], Edna Grünblatt[105,106,107], Tamar Guetta-Baranes[108], Annakaisa Haapasalo[109], Georgios Hadjigeorgiou[110], Harald Hampel[111,112], Olivier Hanon[41], John Hardy[113], Annette M. Hartmann[100], Lucrezia Hausner[97], Janet Harwood[13], Stefanie Heilmann-Heimbach[114], Seppo Helisalmi[115,116], Michael T. Heneka[8,16], Isabel Hernández[9,10], Martin J. Herrmann[83], Per Hoffmann[114], Clive Holmes[117], Henne Holstege[5,16], Raquel Huerto Vilas[36,37], Marc Hulsman[5,16], Charlotte Johansson[102,118], Lena Kilander[20], Anne Kinhult Ståhlbom[102,118], Miia Kivipelto[119,120,121,122], Anne Koivisto[115], Johannes Kornhuber[123], Mary H. Kosmidis[124], Carmen Lage[10,125], Erika J Laukka[103,126], Alessandra Lauria[50], Jenni Lehtisalo[115,127], Ondrej Lerch[128,129], Alberto Lleó[10,24], Adolfo Lopez de Munain[10,130], Malin Löwemark[20], Lauren Luckcuck[13], Juan Macías[76], Catherine A. MacLeod[131], Wolfgang Maier[8,15], Francesca Mangialasche[119], Spallazzi Marco[132], Marta Marquié[9,10], Rachel Marshall[13], Angel Martín Montes[10,94,96], Carmen Martínez Rodríguez[28], Carlo Masullo[133], Simon Mead[134], Patrizia Mecocci[71], Miguel Medina[10,44], Alun Meggy[84], Shima Mehrabian[135], Silvia Mendoza[98], Manuel Menéndez-González[28], Pablo Mir[10,61], Susanne Moebus[136], Merel Mol[137], Laura Molina-Porcel[138,139], Laura Montrreal[9], Laura Morelli[140], Fermin Moreno[10,130], Kevin Morgan[141], Markus M Möthen[114], Carolina Muchnik[58], Benedetta Nacmias[47,142], Tiia Ngandu[127], Gael Nicolas[72], Børge G. Nordestgaard[143,144], Robert Olaso[14], Adelina Orellana[9,10], Michela Orsini[77], Gemma Ortega[9,10], Alessandro Padovani[51], Caffarra Paolo[145], Goran Papenberg[103], Lucilla Parnetti[87], Pau Pastor[25,26], Alba Pérez-Cordón[9], Jordi Pérez-Tur[10,146,147], Pierre Pericard[148], Oliver Peters[149,150], Yolande A.L. Pijnenburg[5], Juan A Pineda[76], Gerard Piñol-Ripoll[36,37], Claudia Pisanu[151], Thomas Polak[83], Julius Popp[152,153,154], Danielle Posthuma[6], Josef Priller[150,155], Raquel Puerta[9],

Olivier Quenez[72], Inés Quintela[69], Jesper Qvist Thomassen[156], Alberto Rábano[10,44], Innocenzo Rainero[52], Inez Ramakers[157], Luis M Real[76,158], Marcel J.T. Reinders[159], Steffi Riedel-Heller[160], Peter Riederer[161], Natalia Roberto[9], Eloy Rodriguez-Rodriguez[10,125], Arvid Rongve[162,163], Irene Rosas Allende[27,28], Maitée Rosende-Roca[9,10], Jose Luis Royo[164], Elisa Rubino[165], Dan Rujescu[100], María Eugenia Sáez[101], Paraskevi Sakka[166], Ingvild Saltvedt[55,167], Ángela Sanabria[9,10], María Bernal Sánchez-Arjona[93], Florentino Sanchez-Garcia[168], Pascual Sánchez Juan[10,125], Raquel Sánchez-Valle[169], Sigrid B Sando[54,55], Michela Scamosci[71], Nikolaos Scarmeas[170,171], Elio Scarpini[34,88], Philip Scheltens[5], Norbert Scherbaum[172], Martin Scherer[173], Matthias Schmid[16,174], Anja Schneider[8,16], Jonathan M. Schott[92], Geir Selbæk[91,175], Davide Seripa[176], Alexey A Shadrin[40], Olivia Skrobot[119], Hilkka Soininen[115], Vincenzo Solfrizzi[78], Alina Solomon[115], Sandro Sorbi[47,142], Oscar Sotolongo-Grau[9], Gianfranco Spalletta[42], Annika Spottke[16], Alessio Squassina[177], Eystein Stordal[178], Juan Pablo Tartan[9], Lluís Tárraga[9,10], Niccolo Tesí[5,16], Anbupalam Thalamuthu[29], Tegos Thomas[30,31], Latchezar Traykov[135], Lucio Tremolizzo[32,33], Anne Tybjærg-Hansen[144,156], Andre Uitterlinden[179], Abbe Ullgren[102], Ingun Ulstein[175], Sergi Valero[9,10], Aad van der Lugt[180], Jasper Van Dongen[2,3,4], Jeroen van Rooij[137], John van Swieten[137], Rik Vandenberghe[181,182], Frans Verhey[157], Jean-Sébastien Vidal[41], Jonathan Vogelgsang[183,184], Martin Vyhnalek[128,129], Michael Wagner[8,16], David Wallon[185], Leonie Weinhold[174], Jens Wiltfang[183,186,187], Gill Windle[131], Bob Woods[131], Mary Yannakoulia[188], Miren Zulaica[10,189], Jan Laczo[128,129], Vaclav Matoska[190], Maria Serpente[88], Francesca Assogna[42], Fabrizio Piras[42], Federica Piras[42], Valentina Ciullo[42], Jacob Shofany[42], Carlo Ferrarese[32,33], Simona Andreoni[32], Gessica Sala[32], Chiara Paola Zoia[32], Maria Del Zompo[177], Alberto Benussi[51], Patrizia Bastiani[191], Mari Takalo*[192], Teemu Natunen*[192], Tiina Laatikainen[120,127], Jaakko Tuomilehto[120,127], Riitta Antikainen[193,194], Timo Strandberg[193,195], Jaana Lindström[127], Markku Peltonen[127], Richard Abraham[196], Ammar Al-Chalabi[197], Nicholas J. Bass[198], Carol Brayne[199], Kristelle S. Brown[200], John Collinge[201], David Craig[202], Pangiotis Deloukas[203], Nick Fox[204], Amy Gerrish[204], Michael Gill[205], Rhian Gwilliam[203], John Hardy[206], Denise Harold[207], Paul Hollingworth[196], Jarret A, Johnston[208], Lesley Jones[196], Brian Lawlor[205], Gill Livingston[198], Simon Lovestone[209], Michelle Lupton[210,211], Aoibhinn Lynch[205], David Mann[212], Bernadette McGuinness[208], Andrew McQuillin[198], Michael C. O'Donovan[196], Michael J. Owen[196], Peter Passmore[208], John F, Powell[210,211], Petra Proitsi[210,211], Martin Rossor[204], Christopher E. Shaw[197], A. David Smith[213], Hugh Gurling[214], Stephen Todd[215], Catherine Mummery[216], Nathalie Ryan[216], Giordano Lacidogna[77], Ad Adarmes-Gómez[10,61], Ana Mauleón[9], Ana Pancho[9], Anna Gailhajenet[9], Asunción Lafuente[9], D Macias-García[10,61], Elvira Martín[9], Esther Pelejà[9], F Carrillo[10,61], Isabel Sastre Merlín[10,95], L Garrote-Espina[10,61], Liliana Vargas[9], M Carrion-Claro[10,61], M Marín[93], Ma Labrador[10,61], Mar Buendia[9], María Dolores Alonso[217], Marina Guitart[9], Mariona Moreno[9], Marta Ibarria[9], Mt Periñán[10,61], Nuria Aguilera[9], P Gómez-Garre[10,61], Pilar Cañabate[9], R Escuela[10,61], R Pineda-Sánchez[10,61], R Vigo-Ortega[10,61], S Jesús[10,61], Silvia Preckler[9], Silvia Rodrigo-Herrero[93], Susana Diego[9], Alessandro Vacca[52], Fausto Roveta[52], Nicola Salvadori[87], Elena Chipi[87], Henning Boecker[15,218], Christoph Laske[219,220], Robert Perneczky[65,221], Costas Anastasiou[188], Daniel Janowitz[62], Rainer Malik[62], Anna Anastasiou[30], Kayenat Parveen[7], Carmen Lage[222], Sara López-García[222], Anna Antonell[169], Kalina Yonkova Mihova[223], Diyana Belezhanska[135], Heike Weber[224], Silvia Kochen[225], Patricia Solis[225], Nancy Medel[225], Julieta Lisso[225], Zulma Sevillano[225], Daniel G Politis[225,226], Valeria Cores[225,226], Carolina Cuesta[225,226], Cecilia Ortiz[227], Juan Ignacio Bacha[227], Mario Rios[228], Aldo Saenz[228], Mariana Sanchez Abalos[229], Eduardo Kohler[230], Dana Lis Palacio[231], Ignacio Etchepareborda[231], Matias Kohler[231], Gisela Novack[232], Federico Ariel Prestia[232], Pablo Galeano[232], Eduardo M. Castaño[232], Sandra Germani[233], Carlos Reyes Toso[233], Matias Rojo[233], Carlos Ingino[233], Carlos Mangone[233], Sebastiaan Engelborghs[234,235,236,237], Tagliavini Fabrizio[238], Sune Fallgaard Nielsen[239], Lucia Farotti[240], Chiara Fenoglio[241], Geert Jan Biessels[242], Seth Love[243], Patrick G. Kehoe[243], Florence Pasquier[244], Christine Van Broeckhoven[2,3,245], David C. Rubinsztein[246], Stefan Teipel[247], Nathalie Fievet[1], Vincent Deramecourt[244], Charlotte Forsell[102,118], Håkan Thonberg[102,118], Maria Bjerke[69], Ellen De Roeck[69], María Teresa Martínez-Larrad[2248], Natividad Olivar[233], Mohsen Ghanbari[11], Perminder Sachdev[29], Karen Mather[29], Frank Jessen[8,16], M. Arfan Ikram[11], Alexandre de Mendonça[89], Jakub Hort[128,129], Tsolaki Magda[30,31], Philippe Amouyel[1], Julie Williams[13], Ruth Frikke-Schmidt[144,156], Jordi Clarimon[10,24], Jean-

François Deleuze[14], Giacomina Rossi[85], Ole A. Andreassen[40], Martin Ingelsson[20], Mikko Hiltunen[17], Kristel Sleegers[2,3,4], Cornelia M. van Duijn[11,12], Rebecca Sims[13], Wiesje M. van der Flier[5], Agustín Ruiz[9,10], Alfredo Ramirez[7,8,16,249], Jean-Charles Lambert[1]

1. Univ. Lille, Inserm, CHU Lille, Institut Pasteur de Lille, U1167-RID-AGE facteurs de risque et déterminants moléculaires des maladies liés au vieillissement, Lille, France
2. Complex Genetics of Alzheimer's Disease Group, VIB Center for Molecular Neurology, VIB, Antwerp, Belgium
3. Laboratory of Neurogenetics, Institute Born - Bunge, Antwerp, Belgium
4. Department of Biomedical Sciences, University of Antwerp, Neurodegenerative Brain Diseases Group,
5. Alzheimer Center Amsterdam, Department of Neurology, Amsterdam Neuroscience, Vrije Universiteit Amsterdam, Amsterdam UMC, Amsterdam, The Netherlands
6. Department of Complex Trait Genetics, Center for Neurogenomics and Cognitive Research, Amsterdam, The Netherlands
7. Division of Neurogenetics and Molecular Psychiatry, Department of Psychiatry and Psychotherapy, University of Cologne, Medical Faculty, Cologne, Germany.
8. Department of Neurodegenerative Diseases and Geriatric Psychiatry, University Hospital Bonn, Bonn, Germany
9. Research Center and Memory clinic Fundació ACE, Institut Català de Neurociències Aplicades, Universitat Internacional de Catalunya, Barcelona, Spain
10. CIBERNED, Network Center for Biomedical Research in Neurodegenerative Diseases, National Institute of Health Carlos III, Madrid, Spain
11. Department of Epidemiology, ErasmusMC, Totterdam, The Netherlands
12. Nuffield Department of Population Health Oxford University, Oxford, UK
13. MRC Centre for Neuropsychiatric Genetics and Genomics, , Division of Psychological Medicine and Clinical
14. Université Paris-Saclay, CEA, Centre National de Recherche en Génomique Humaine, 91057, Evry, France
15. German Center for Neurodegenerative Diseases (DZNE Bonn), Bonn, Germany
16. Section Genomics of Neurdegenerative Diseases and Aging, Department of Human Genetics Amsterdam, The Netherlands
17. Institute of Biomedicine, University of Eastern Finland, Kuopio, Finland
18. Brain Institute, Federal University of Rio Grande do Norte, Av. Nascimento de Castro 2155 Natal, Brazil
19. LACDR, Leiden, The Netherlands
20. Dept.of Public Health and Carins Sciences / Geriatrics, Uppsala University, Sweden
21. Centre of Age-Related Medicine, Stavanger University Hospital, Norway
22. Institute of Psychiatry, Psychology & Neuroscience, PO 70, 16 De Crespigny Park, London, UK
23. Department of Surgery, Biochemistry and Molecular Biology, School of Medicine, University of Málaga, Málaga, Spain.
24. Department of Neurology, II B Sant Pau, Hospital de la Santa Creu i Sant Pau, Universitat Autònoma de Barcelona, Barcelona, Spain.
25. Fundació Docència i Recerca MútuaTerrassa and Movement Disorders Unit, Department of Neurology, University Hospital MútuaTerrassa, Terrassa 08221, Barcelona, Spain
26. Memory Disorders Unit, Department of Neurology, Hospital Universitari Mutua de Terrassa, Terrassa, Barcelona, Spain.
27. Laboratorio de Genética. Hospital Universitario Central de Asturias, Oviedo, Spain

28. Servicio de Neurología HOspital Universitario Central de Asturias- Oviedo and Instituto de Investigación Biosanitaria del Principado de Asturias, Oviedo, Spain
29. Centre for Healthy Brain Ageing, School of Psychiatry, Faculty of Medicine, University of New South Wales, Sydney, Australia
30. 1st Department of Neurology, Medical school, Aristotle University of Thessaloniki, Thessaloniki, Makedonia, Greece
31. Alzheimer Hellas, Thessaloniki, Makedonia, Greece
32. School of Medicine and Surgery, University of Milano-Bicocca, Italy
33. Neurology Unit, "San Gerardo" hospital, Monza, Italy
34. Fondazione IRCCS Ca' Granda, Ospedale Policlinico, Milan, Italy
35. Department of Laboratory Diagnostics, III Laboratory of Analysis, Brescia Hospital, Brescia, Italy
36. Unitat Trastorns Cognitius, Hospital Universitari Santa Maria de Lleida, Lleida, Spain
37. Institut de Recerca Biomedica de Lleida (IRBLLeida), Lleida, Spain
38. Department of Clinical Sciences and Community Health, University of Milan, Italy
39. Geriatic Unit, Fondazione Cà Granda, IRCCS Ospedale Maggiore Policlinico, Milan, Italy
40. NORMENT Centre, University of Oslo, Oslo, Norway
41. Université de Paris, EA 4468, APHP, Hôpital Broca, Paris, France
42. Laboratory of Neuropsychiatry, Department of Clinical and Behavioral Neurology, IRCCS Santa Lucia Foundation, Rome, Italy
43. Servei de Neurologia, Hospital Universitari i Politècnic La Fe, Valencia, Spain.
44. CIEN Foundation/Queen Sofia Foundation Alzheimer Center, Madrid, Spain
45. Molecular Markers Laboratory, IRCCS Istituto Centro San Giovanni di Dio Fatebenefratelli, Brescia, Italy
46. Univ. Montpellier, Inserm U1061, Neuropsychiatry: epidemiological and clinical research, PSNREC, Montpellier, France
47. Department of Neuroscience, Psychology, Drug Research and Child Health University of Florence, Florence Italy
48. Azienda Ospedaliero-Universitaria Careggi, Florence, Italy
49. MAC - Memory Clinic, IRCCS Istituto Centro San Giovanni di Dio Fatebenefratelli, Brescia
50. Geriatrics Unit Fondazione Policlinico A. Gemelli IRCCS, Rome , Italy
51. Centre for Neurodegenerative Disorders, Department of Clinical and Experimental Sciences, University of Brescia, Brescia, Italy
52. Department of Neuroscience “Rita Levi Montalcini”, University of Torino, Torino, Italy
53. Experimental Neuro-psychobiology Laboratory,Department of Clinical and Behavioral Neurology, IRCCS Santa Lucia Foundation, Rome, Italy
54. Department of Neurology and Clinical Neurophysiology, University Hospital of Trondheim, Trondheim, Norway
55. Department of Neuromedicine and Movement Science, Norwegian University of Science and Technology, Trondheim, Norway
56. Dementia Centre for Research Collaboration, School of Psychiatry, University of New South Wales, Sydney, Australia
57. Biosciences, School of Science and Technology, Nottingham Trent University, Nottingham UK
58. Centro de Neuropsiquiatría y Neurología de la Conducta (CENECON), Facultad de Medicina, Universidad de Buenos Aires (UBA), C.A.B.A, Buenos Aires, Argentina.
59. Departamento Ciencias Fisiológicas UAII, Facultad de Medicina, UBA, C.A.B.A, Buenos Aires, Argentina.
60. Hospital Interzonal General de Agudos Eva Perón, San Martín, Buenos Aires, Argentina.

61. Unidad de Trastornos del Movimiento, Servicio de Neurología y Neurofisiología. Instituto de Biomedicina de Sevilla (IBiS), Hospital Universitario Virgen del Rocío/CSIC/Universidad de Sevilla, Seville, Spain
62. Institute for Stroke and Dementia Research, Klinikum der Universität München, Ludwig-Maximilians-Universität LMU, Munich, Germany.
63. German Center for Neurodegenerative Diseases (DZNE, Munich), Munich, Germany.
64. Faculty of Medical & Health Sciences, University of Auckland, New Zealand
65. Wales Centre for Ageing & Dementia Research, Swansea University, Wales, New Zealand
66. UFIEC, Instituto de Salud Carlos III, , Madrid, Spain
67. Inserm, Bordeaux Population Health Research Center, UMR 1219, Univ. Bordeaux, ISPED, CIC 1401-EC, Univ Bordeaux,Bordeaux, France
68. CHU de Bordeaux, Pole santé publique, Bordeaux, France
69. Grupo de Medicina Xenómica, Centro Nacional de Genotipado (CEGEN-PRB3-ISCIII). Universidade de Santiago de Compostela, Santiago de Compostela, Spain.
70. Fundación Pública Galega de Medicina Xenómica- CIBERER-IDIS, University of Santiago de Compostela, Santiago de Compostela, Spain.
71. Institute of Gerontology and Geriatrics, Department of Medicine and Surgery, University of Perugia Perugia, Italy
72. Normandie Univ, UNIROUEN, Inserm U1245 and CHU Rouen, Department of Genetics and CNR-MAJ, Rouen, France
73. Unit of Clinical Pharmacology, University Hospital of Cagliari, Cagliari, Italy
74. Radboudumc Alzheimer Center, Department of Geriatrics, Radboud University Medical Center, Nijmegen, the Netherlands
75. Institute for Regenerative Medicine, University of Zürich, Schlieren, Switzerland
76. Unidad Clínica de Enfermedades Infecciosas y Microbiología. Hospital Universitario de Valme, Sevilla, Spain
77. Department of Neuroscience, Catholic University of Sacred Heart, Fondazione Policlinico Universitario A. Gemelli IRCCS, Rome, Italy
78. University of Bari, “A. Moro”, Bari, Italy
79. School of Medicine, University of Thessaly, Larissa, Greece
80. University Bordeaux, Inserm, Bordeaux Population Health Research Center, France
81. Department of Neurology, University Medical Center Groningen, the Netherlands
82. Department of Neurology, Bordeaux University Hospital, Bordeaux, France
83. Department of Psychiatry, Psychosomatics and Psychotherapy, Center of Mental Health, University Hospital, Wuerzburg
84. UKDRI@ Cardiff, School of Medicine, Cardiff University, Cardiff, UK
85. Munich Cluster for Systems Neurology (SyNergy), Munich, Germany.
86. Technical University of Munich, School of Medicine, Klinikum rechts der Isar, Department of Psychiatry and Psychotherapy, Munich, Germany
87. Institute of Cognitive Neurology and Dementia Research (IKND), Otto-Von-Guericke University, Magdeburg, Germany.
88. German Center for Neurodegenerative Diseases (DZNE), Magdeburg, Germany.
89. Faculty of Medicine, University of Lisbon, Portugal
90. Department of Psychiatry, Social Medicine Center East- Donauspital, Vienna, Austria
91. Institute of Clinical Medicine, University of Oslo, Oslo, Norway.
92. Dementia Research Centre, UCL Queen Square Institute of Neurology, London, United Kingdom
93. Unidad de Demencias, Servicio de Neurología y Neurofisiología. Instituto de Biomedicina de Sevilla (IBiS), Hospital Universitario Virgen del Rocío/CSIC/Universidad de Sevilla, Seville, Spain

94. Instituto de Investigacion Sanitaria ‘Hospital la Paz’ (IdIPaz), Madrid, Spain
95. Centro de Biología Molecular Severo Ochoa (UAM-CSIC), Madrid, Spain
96. Hospital Universitario la Paz, Madrid, Spain
97. Department of geriatric Psychiatry, Central Institute for Mental Health, Mannheim, University of Heidelberg, Germany
98. Alzheimer Research Center & Memory Clinic, Andalusian Institute for Neuroscience, Málaga, Spain.
99. Hospital Universitario Ramon y Cajal, IRYCIS, Madrid
100. Department of Psychiatry and Psychotherapy, Medical University of Vienna, Vienna, Austria
101. CAEBI, Centro Andaluz de Estudios Bioinformáticos, Sevilla, Spain.
102. Karolinska Institutet, Center for Alzheimer Research, Department NVS, Division of Neurogeriatrics, Stockholm, Sweden
103. Aging Research Center, Department of Neurobiology, Care Sciences and Society, Karolinska Institutet and Stockholm University, Stockholm, Sweden
104. Institute of Public Health, University of Cambridge, UK
105. Department of Child and Adolescent Psychiatry and Psychotherapy, University Hospital of Psychiatry Zurich, University of Zurich, Zurich, Switzerland
106. Neuroscience Center Zurich, University of Zurich and ETH Zurich, Switzerland
107. Zurich Center for Integrative Human Physiology, University of Zurich, Switzerland
108. Human Genetics, School of Life Sciences, Life Sciences Building, University Park, University of Nottingham, Nottingham, UK
109. A.I Virtanen Institute for Molecular Sciences, University of Eastern Finland, Kuopio, Finland
110. Department of Neurology, Medical School, University of Cyprus, Cyprus
111. Sorbonne University, GRC n° 21, Alzheimer Precision Medicine Initiative (APMI), AP-HP, Pitié-Salpêtrière Hospital, Boulevard de l'hôpital, Paris, France
112. Eisai Inc., Neurology Business Group, 100 Tice Blvd, Woodcliff Lake, NJ 07677, USA
113. Reta Lila Weston Research Laboratories, Department of Molecular Neuroscience, UCL Institute of Neurology, London, UK.
114. Institute of Human Genetics, University of Bonn, School of Medicine & University Hospital Bonn, Bonn, Germany
115. Insitute of Clinical Medicine - Neurology, University of Eastern, Kuopio, Finland
116. Institute of Clinical Medicine – Internal Medicine, University of Eastern Finland, Kuopio, Finland
117. Clinical and Experimental Science, Faculty of Medicine, University of Southampton, Southampton, UK.
118. Unit for Hereditary dementias, Karolinska University Hospital-Solna, Stockholm, Sweden
119. Division of Clinical Geriatrics, Center for Alzheimer Research, Care Sciences and Society (NVS), Karolinska Institutet, Stockholm, Sweden
120. Institute of Public Health and Clinical Nutrition, University of Eastern Finland, Kuopio, Finland
121. Neuroepidemiology and Ageing Research Unit, School of Public Health, Imperial College London, London, United Kingdom
122. Stockholms Sjukhem, Research & Development Unit, Stockholm, Sweden
123. Department of Psychiatry and Psychotherapy, Universitätsklinikum Erlangen, and Friedrich-Alexander Universität Erlangen-Nürnberg, Erlangen, Germany.
124. Laboratory of Cognitive Neuroscience, School of Psychology, Aristotle University of Thessaloniki, Thessaloniki, Greece
125. Neurology Service, Marqués de Valdecilla University Hospital (University of Cantabria and IDIVAL), Santander, Spain.
126. Stockholm Gerontology Research Center, Stockholm, Sweden

127. Public Health Promotion Unit, Finnish Institute for Health and Welfare, Helsinki, Finland
128. Memory Clinic, Department of Neurology, Charles University, 2nd Faculty of Medicine and Motol University Hospital, Czech Republic
129. International Clinical Research Center, St. Anne’s University Hospital Brno, Brno, Czech Republic
130. Department of Neurology. Hospital Universitario Donostia. OSAKIDETZA-Servicio Vasco de Salud, San Sebastian, Spain
131. School of Health Sciences, Bangor University, UK
132. Unit of Neurology, University of Parma and AOU, Parma, Italy
133. Institute of Neurology, Catholic University of the Sacred Heart , Rome, Itlay
134. MRC Prion Unit at UCL, UCL Institute of Prion Diseases, London, UK
135. Clinic of Neurology, UH "Alexandrovska", Medical University - Sofia, Sofia, Bulgaria
136. Institute for Urban Public Health, University Hospital of University Duisburg-Essen, Essen, Germany
137. Department of Neurology, ErasmusMC, Rotterdam, The Netherlands
138. Neurological Tissue Bank of the Biobanc-Hospital Clinic-IDIBAPS, Institut d'Investigacions Biomèdiques August Pi i Sunyer, Barcelona, Spain.
139. Alzheimer’s disease and other cognitive disorders Unit. Neurology Department, Hospital Clinic , Barcelona, Spain
140. Laboratory of Brain Aging and Neurodegeneration- FIL-CONICET, Buenos Aires, Argentina
141. Human Genetics, School of Life Sciences, University of Nottingham, UK
142. IRCCS Fondazione Don Carlo Gnocchi, Florence, Italy
143. Department of Clinical Biochemistry, Herlev and Gentofte Hospital, Herlev, Denmark
144. Department of Clinical Medicine, University of Copenhagen, Copenhagen, Denmark
145. DIMEC, University of Parma, Parma, Italy
146. Institut de Biomedicina de València-CSIC (valència, Spain) CIBERNED.
147. Unitat Mixta de de Neurología y Genética, Institut d'Investigació Sanitària La Fe (València, Spain)
148. Univ. Lille, CNRS, Inserm, CHU Lille, Institut Pasteur de Lille, US 41-UMS 2014-PLBS, bilille, Lille, France.
149. Institute of Psychiatry and Psychotherapy, Charité-Universitätsmedizin Berlin, Corporate Member of Freie Universität Berlin, Humboldt-Universität Zu Berlin, and Berlin Institute of Health, Berlin, Germany.
150. German Center for Neurodegenerative Diseases (DZNE), Berlin, Germany.
151. Department of Biomedical Sciences, University of Cagliari, Italy
152. CHUV, Old Age Psychiatry, Department of Psychiatry, Lausanne, Switzerland
153. Old Age Psychiatry, Department of Psychiatry, Lausanne University Hospital, Lausanne, Switzerland
154. Department of Geriatric Psychiatry, University Hospital of Psychiatry Zürich, Zürich, Switzerland
155. Department of Neuropsychiatry and Laboratory of Molecular Psychiatry, Charité, Charitéplatz 1, 10117 Berlin, Germany
156. Department of Clinical Biochemistry, Rigshospitalet, Copenhagen, Denmark
157. Maastricht University, Department of Psychiatry & Neuropsychologie, Alzheimer Center Limburg, Maastricht, the Netherlands
158. Depatamento de Especialidades Quirúrgicas, Bioquímica e Inmunología. Facultad de Medicina. Universidad de Málaga. Málaga, Spain
159. Delft Bioinformatics Lab, Delft University of Technology, Delft, The Netherlands
160. Institute of Social Medicine, Occupational Health and Public Health, University of Leipzig, 04103 Leipzig, Germany.

161. Center of Mental Health, Clinic and Policlinic of Psychiatry, Psychosomatics and Psychotherapy, University Hospital of Würzburg, Wuerzburg, Germany
162. Department of Research and Innovation, Helse Fonna, Haugesund Hospital, Haugesund, Norway.
163. The University of Bergen, Institute of Clinical Medicine (K1), Bergen Norway
164. Departamento de Especialidades Quirúrgicas, Bioquímicas e Inmunología, School of Medicine, University of Málaga, Málaga, Spain.
165. Department of Neuroscience and Mental Health, AOU Città della Salute e della Scienza di Torino, Torino, Italy
166. Athens Association of Alzheimer’s disease and Related Disorders, Athens, Greece
167. Department of Geriatrics, St. Olav’s Hospital, Trondheim University Hospital, Norway
168. Department of Immunology, Hospital Universitario Doctor Negrín, Las Palmas de Gran Canaria, Spain.
169. Neurology department-Hospital Clínic, IDIBAPS, Universitat de Barcelona, Barcelona, Spain.
170. Taub Institute for Research in Alzheimer’s Disease and the Aging Brain, The Gertrude H. Sergievsky Center, Depatment of Neurology, Columbia University, New York, NY
171. 1st Department of Neurology, Aiginition Hospital, National and Kapodistrian University of Athens, Medical School, Greece
172. LVR-Hospital Essen, Department of Psychiatry and Psychotherapy, Medical Faculty, University of Duisburg-Essen, Virchowstr. 174, 45147 Essen, Germany
173. Department of Primary Medical Care, University Medical Centre Hamburg-Eppendorf, 20246 Hamburg, Germany.
174. Institute of Medical Biometry, Informatics and Epidemiology, University Hospital of Bonn, Bonn, Germany.
175. Department of Geriatric Medicine, Oslo University Hospital, Oslo, Norway
176. Laboratory for Advanced Hematological Diagnostics, Department of Hematology and Stem Cell Transplant, Lecce, Italy
177. Department of Biomedical Sciences, Section of Neuroscience and Clinical Pharmacology, University of Cagliari, Italy
178. Department of Psychiatry, Namsos Hospital, Namsos, Norway
179. Department of Internal medicine and Biostatistics, ErasmusMC, Rooterdam, The Netherlands
180. Department of Radiology&Nuclear medicine, ErasmusMC, Totterdam, The Netherlands
181. Laboratory for Cognitive Neurology, Department of Neurosciences, University of Leuven, Belgium
182. Neurology Department, University Hospitals Leuven, Leuven, Belgium
183. Department of Psychiatry and Psychotherapy, University Medical Center Goettingen, Goettingen, Germany
184. Department of Psychiatry, Harvard Medical School, McLean Hospital, Belmont, MA, USA
185. Normandie Univ, UNIROUEN, Inserm U1245, CHU Rouen, Department of Neurology and CNR-MAJ, F 76000, Normandy Center for Genomic and Personalized Medicine, Rouen, France
186. German Center for Neurodegenerative Diseases (DZNE), Goettingen, Germany
187. Medical Science Department, iBiMED, Aveiro, Portugal
188. Department of Nutrition and Diatetics, Harokopio University, Athens, Greece
189. Neurosciences Area. Instituto Biodonostia. San Sebastian, Spain
190. Department of Clinical Biochemistry, Hematology and Immunology, Na Homolce Hospital, Prague, Czech republic
191. Institute of Gerontology and Geriatrics, Department of Medicine, University of Perugia Perugia (Italy)

192. Insitute of Biomedicine, University of Eastern Finland, Finland
193. Center for Life Course Health Research, University of Oulu, Oulu, Finland
194. Medical Research Center Oulu, Oulu University Hospital, Oulu, Finland
195. University of Helsinki and Helsinki University Hospital, Helsinki, Finland
196. Division of Psychological Medicine and Clinial Neurosciences, MRC Centre for Neuropsychiatric Genetics and Genomics, Cardiff University, UK
197. Kings College London, Institute of Psychiatry, Psychology and Neuroscience, UK
198. Division of Psychiatry, University College London, UK
199. Institute of Public Health, University of Cambridge, Cambridge, UK
200. Institute of Genetics, Queens Medical Centre, University of Nottingham, Nottingham, UK
201. XXX
202. Ageing Group, Centre for Public Health, School of Medicine, Dentistry and Biomedical Sciences, Queen's University Belfast, UK
203. The Wellcome Trust Sanger Institute, Wellcome Trust Genome Campus, Hinxton, Cambridge, UK.
204. Dementia Research Centre, Department of Neurodegenerative Disease, UCL Institute of Neurology, London, UK
205. Mercer's Institute for Research on Ageing, St James' Hospital, Dublin, Ireland
206. Department of Molecular Neuroscience, UCL, Institute of Neurology, London, UK
207. School of Biotechnology, Dublin City University, Dublin, Ireland
208. Centre for Public Health, School of Medicine, Dentistry and Biomedical Sciences, Queens University, Belfast, UK
209. Department of Psychiatry, University of Oxford, Oxford, UK
210. Department of Basic and Clinical Neuroscience, Institute of Psychiatry, Psychology and Neuroscience, Kings College London, London UK
211. Genetic Epidemiology, QIMR Berghofer Medical Research Institute, Herston, Queensland, Australia
212. Division of Neuroscience and Experimental Psychology, School of Biological Sciences, Faculty of Biology, Medicine and Health, University of Manchester, Manchester Academic Health Science Centre, Manchester M13 9PT, UK
213. Oxford Project to Investigate Memory and Ageing (OPTIMA), University of Oxford, Level 4, John Radcliffe Hospital, Oxford, UK
214. Department of Mental Health Sciences, University College London, London, UK
215. Ageing Group, Centre for Public Health, School of Medicine, Dentistry and Biomedical Sciences, Queen’s University Belfast, Belfast, UK.
216. Dementia Research Centre, UCL, London, UK
217. Servei de Neurologia. Hospital Clínic Universitari de València, Spain
218. Department of Radiology, University Hospital Bonn, Bonn, Germany
219. German Center for Neurodegenerative Diseases (DZNE), Tübingen, Germany
220. Section for Dementia Research, Hertie Institute for Clinical Brain Research and Department of Psychiatry, Tübingen, Germany
221. Department of Psychiatry and Psychotherapy, University Hospital, LMU Munich, Munich, Germany
222. Service of Neurology, University Hospital Marqués de Valdecilla, IDIVAL, University of Cantabria, Santander, Spain
223. Molecular Medicine Center, Department of Medical chemistry and biochemistry, Medical University of Sofia, Bulgaria

224. Department of Psychiatry, Psychosomatics and Psychotherapy, Center of Mental Health, University Hospital of Würzburg, Germany
225. ENYS (Estudio en Neurociencias y Sistemas Complejos) CONICET- Hospital El Cruce "Nestor Kirchner"- UNAJ, Argentina
226. HIGA Eva Perón, Buenes Aires, Agentina
227. Neurología Clinica, Buenes Aires, Agentina
228. Dirección de Atención de Adultos Mayores del Min. Salud Desarrollo Social y Deportes de la Pcia. de Mendoza, Argentina
229. Laboratorio de Genética Forense del Ministerio Público de la Pcia. de La Pampa, Argentina
230. Fundacion Sinapsis, Santa Rosa, Argentina
231. Hospital Dr. Lucio Molas, Santa Rosa; Fundacion Ayuda Enfermo Renal y Alta Complejidad (FERNAC), Santa Rosa, Argentina
232. Laboratory of Brain Aging and Neurodegeneration- FIL, Buneos Aires, Argentina
233. Centro de Neuropsiquiatría y Neurología de la Conducta (CENECON), Facultad de Medicina, Universidad de Buenos Aires (UBA), C.A.B.A, Buenos Aires, Argentina
234. Center for Neurosciences, Vrije Universiteit Brussel (VUB), Brussels, Belgium
235. Reference Center for Biological Markers of Dementia (BIODEM), Institute Born-Bunge, University of Antwerp, Antwerp, Belgium
236. Institute Born-Bunge, University of Antwerp, Antwerp, Belgium
237. Department of Neurology, UZ Brussel, Brussels, Belgium
238. Fondazione IRCCS, Istituto Neurologico Carlo Besta, Milan Italy
239. Department of Clinical Biochemistry, Herlev and Gentofte Hospital, Herlev Denmark
240. Centre for Memory Disturbances, Lab of Clinical Neurochemistry, Section of Neurology, University of Perugia, Italy
241. University of Milan, Milan, Italy
242. Department of Neurology, UMC Utrecht Brain Center, Utrecht, the Netherlands
243. Translational Health Sciences, Bristol Medical School, University of Bristol, Bristol, BS16 1LE, UK
244. Univ Lille Inserm 1172, CHU Clinical and Research Memory Research Centre (CMRR) of Distalz, Licend, Lille France
245. Neurodegenerative Brain Diseases Group, VIB Center for Molecular Neurology, VIB, Antwerp, Belgium
246. Cambridge Institute for Medical Research and UK Dementia Research Institute, University of Cambridge, Cambridge, UK
247. German Center for Neurodegenerative Diseases (DZNE), Rostock, Germany
248. Centro de Investigación Biomédica en Red de Diabetes y Enfermedades Metabólicas Asociadas, CIBERDEM, Spain, Hospital Clínico San Carlos, Madrid, Spain
249. Glenn Biggs Institute for Alzheimer’s and Neurodegenerative Diseases, San Antonio, TX, USA

EADI

Céline Bellenguez[1], Benjamin Grenier-Boley[1], Jacques Epelbaum[2], David Wallon[3], Didier Hannequin[3], Florence Pasquier[4], Claudine Berr[5], Jean-Francois Dartigues[6], Dominique campion[7], Christophe Tzourio[8], Vincent Dermecourt[4], Nathalie Fievet[1], Olivier Hanon[9], Carole Dufouil[8], Alexis Brice[10], Bruno Dubois[11], Karen Ritchie[5], Phillippe Amouyel[1], Jean-Charles Lambert[1]

1. Univ. Lille, Inserm, CHU Lille, Institut Pasteur Lille, U1167-RID-AGE - Facteurs de risque et déterminants moléculaires des maladies liées au vieillissement, F-59000 Lille, France
2. UMR 894, Center for Psychiatry and Neuroscience, INSERM, Université Paris Descartes, F-75000 Paris , France
3. Normandie Univ, UNIROUEN, Inserm U1245, CHU Rouen, Department of Neurology and CNR-MAJ, F 76000, Normandy Center for Genomic and Personalized Medicine, Rouen, France
4. Univ. Lille, Inserm, CHU Lille, UMR1172, Resources and Research Memory Center (MRRC) of Distalz, Licend, Lille France
5. Univ. Montpellier, Inserm U1061, Neuropsychiatry: epidemiological and clinical research, PSNREC, Montpellier, France
6. University Bordeaux, Inserm, Bordeaux Population Health Research Center, France
7. Normandie Univ, UNIROUEN, Inserm U1245 and CHU Rouen, Department of Genetics and CNR-MAJ, Rouen, France
8. University Bordeaux, Inserm, Bordeaux Population Health Research Center, France
9. Université de Paris, EA 4468, APHP, Hôpital Broca, Paris, France
10. Inserm U1127, CNRS UMR7225, Sorbonne Universités, UPMC Univ Paris 06, UMR_S1127, Institut du Cerveau et de la Moelle épinière, F-75013, Paris, France; 22. APHP, Department of genetics, Pitié-Salpêtrière Hospital, 75013, Paris, France
11. Institut de la Mémoire et de la Maladie d'Alzheimer (IM2A), Département de Neurologie, Hôpital de la Pitié-Salpêtrière, AP-HP, Paris, France; Institut des Neurosciences Translationnelles de Paris (IHU-A-ICM), Institut du Cerveau et de la Moelle Epinière (ICM), Paris, France; 26. INSERM, CNRS, UMR-S975, Institut du Cerveau et de la Moelle Epinière (ICM), Paris, France; Sorbonne Universités, Université Pierre et Marie Curie, Hôpital de la Pitié-Salpêtrière, AP-HP, Paris, France

Supplemental Methods

Phenotype Ascertainment

Cohorts and Phenotype Ascertainment

In the current study, we used data from twenty-nine cohorts and three sequencing projects related to AD[1-23]. Details on phenotype ascertainment are described elsewhere[1–6]. Briefly, all individuals with a diagnosis of AD met National Institute of Neurological and Communicative Disorders and Stroke/Alzheimer's Disease and Related Disorders Association (NINCDS-ADRDA) criteria for probable or possible late onset AD[7], or met Diagnosis and Statistical Manual of Mental Disorders IV-V (DSMIV-V) criteria[8–10], or had a clinical dementia rating (CDR® Dementia Staging Instrument[11]) > 0.5. Some cohorts verified AD diagnoses by means of neuropathology, using Braak staging[12], CERAD scoring[13], or National Institute on Aging Reagan (NIA-Reagan) 1997 criteria[14]. Cognitively normal subjects (controls) did not have AD according to the above clinical criteria for AD, did not have a diagnosis of MCI, and had a CDR of 0 and/or Mini-Mental State Examination (MMSE[15]) > 25. In the MIRAGE cohort, control status was evaluated through a Modified Telephone Interview of Cognitive Status score ≥ 86 (a telephone version of the MMSE)[16].

Further, the National Alzheimer's Coordinating Center (NACC), Rush University Religious Orders Study/Memory and Aging Project (ROSMAP), and Alzheimer's Disease Neuroimaging Initiative (ADNI), are longitudinal cohorts that provide detailed information regarding clinical status (control, MCI, demented) and presumed disease etiology at repeated examinations. Additionally, deceased subjects are assessed for neuropathology. Where possible, in NACC, a final diagnoses of MCI or possible/probable/definite AD was obtained using NIA Alzheimer's Association (NIA-AA) 2011 criteria[17,18]. In all three cohorts, AD diagnoses were verified by neuropathology as middle or high AD likelihood following NIA-Reagan 1997 criteria (moderate to frequent neuritic plaques and Braak stage III-VI)[14]. In concordance with the category "possible AD dementia with evidence of the AD pathophysiological process" from the NIA-AA 2011 criteria[17], we attributed possible AD diagnoses to subjects who met clinical criteria for non-AD dementia but also met AD neuropathological criteria. In concordance with the NIA-AA 2011/2012 framework[18,19], we also evaluated neuropathology in MCI subjects to verify presumed AD etiology (cf. page 5). Controls were not re-evaluated based on neuropathology data. Subjects that reverted from dementia to control status during longitudinal follow-up were excluded. Additional cohort-specific details are listed below.

NACC

Genotyping waves 1 through 7 from the Alzheimer's Disease Centers (ADC1-7) and a subset of the ADSP projects include subjects ascertained and evaluated by the clinical and neuropathological cores of 32 NIA-funded ADCs. NACC coordinates the collection of these phenotypes, implements diagnoses (cognitively normal, cognitively impaired but not MCI, MCI, demented; and presumed disease etiology) and then provides all data to researchers under the form of the Minimum Data Set (MDS), Uniform Data Set (UDS)[20–22], and Neuropathology data set (NP)[23]. The MDS represents an older subset of the NACC data and only contains cross-sectional data, while the more recent UDS provides longitudinal phenotypes and covariates. Since 2015, the UDS was updated to incorporate the NIA-AA 2011 criteria for MCI and AD[18,24]. In the current study, we used the UDS and NP for which data was collected between September 2005 and March 2020. The ADC1-7 data sets covered 7,627 subjects in the UDS and 2,629 subjects in the MDS.

Subjects that had a diagnosis of Down syndrome, central nervous system neoplasm, bipolar disorder, schizophrenia, alcohol-induced dementia, or substance-abuse-induced dementia, were excluded. Subjects carrying mutations of dominantly inherited AD or frontotemporal lobar degeneration (FTLD) were also excluded. Subjects with a final diagnosis of MCI or dementia, for which the etiology was unknown, not due to AD, or only secondary due to AD (and without AD neuropathological information), were excluded. Subjects with a final diagnosis of "cognitively impaired but not MCI", but having no other neurological disorder, were kept as controls, considering that this more consistently matched control criteria in many of the other cohorts considered in this study.

ROSMAP

In ROSMAP, subjects were diagnosed at each visit: as possible/probable AD according to NINCDS-ADRDA criteria[7]; as MCI when judged to have cognitive impairment but not meeting dementia criteria according to the clinician; or as control when there was no cognitive impairment or the subject did not meet dementia criteria[25,26]. At time of death, a final clinical diagnosis was made by an expert neurologist, followed by case conference consensus review (blinded to postmortem data)[27].

ADNI

In ADNI, subjects were diagnosed at regular visits: as possible/probable AD according to NINCDS-ADRDA criteria[7]; as MCI according to Petersen/Winblad criteria; or as control when not demented, not MCI, CDR = 0, and MMSE > 28. Neuropathology assessments followed the NACC NP framework.

Discovery Samples Phenotype Harmonization

The discovery sample contained many subjects that were genotyped multiple times across different studies. This largely reflected efforts from the ADGC, ADSP, and AMP-AD, to perform next generation sequencing (NGS) on existing cohort samples for the purpose of rare variant discovery and AD gene prioritization. In other instances, participants were recruited in different studies at different times. Therefore, to handle potential duplicate discordance and phenotype heterogeneity, we implemented a cross-sample phenotype harmonization procedure aiming to standardize pathology-verified diagnoses where possible, share unique missing information across all duplicate entries of a given subject, resolve longitudinal changes in diagnosis, and flag subjects with unresolvable duplicate discordance for exclusion.

Duplicate samples were identified by determining genetic cryptic relatedness (cf. page 6 below), but for the purpose of sample cross-referencing did not include known identical twins in LOAD and ROSMAP samples. First, duplicate samples were flagged as discordant if their age-at-death information differed by more than 2 years or if pathology measures (Braak or neuritic plaque density) differed. Across all cohorts, where possible, AD diagnoses were verified by neuropathology as middle or high AD likelihood following NIA-Reagan 1997 criteria (moderate to frequent neuritic plaques and Braak stage III-VI)[14]. Additionally, when only either neuritic plaque or Braak information was available and in line with NIA-Reagan 1997 middle or high AD likelihood criteria, and/or the cohort/project demographics provided a diagnosis of definite AD, the subject was considered to have pathology-verified AD status. Cognitively normal (CN) subjects with evidence of AD pathology were kept as CN. Further, if at least one entry across duplicate samples indicated a diagnosis of Down syndrome, central nervous system neoplasm, bipolar disorder, schizophrenia, alcohol-induced dementia, substance-abuse-induced dementia, neurological (not including Parkinson's disease) or systemic disease despite being cognitively normal, or carrying mutations of dominantly inherited AD or frontotemporal lobar degeneration (FTLD), then all duplicate samples were marked as such and flagged for exclusion. Extending on the above, all genetic samples were checked for the presence of known pathogenic mutations on *APP, PSEN1, PSEN2* and *MAPT*, whereby carriers and their duplicate samples were flagged for exclusion.

Then, duplicate samples with differing age entries (i.e. longitudinal changes) were evaluated. Reversions from AD or dementia to MCI status, or from MCI to cognitively normal (CN) status, were permitted, but reversions from AD or non-AD dementia to CN status were flagged for exclusion. "Reversions" from AD to non-AD dementia status were permitted, unless pathology (cf. above) indicated the presence of AD pathology, thereby marking the subject as AD. Vice versa, "conversions" from non-AD

dementia to AD status were permitted, unless pathology (cf. above) indicated no presence of AD pathology, thereby marking the subject as non-AD dementia. All other types of conversions were directly permitted. Then, duplicate samples for which the diagnoses at the oldest shared age entries differed, or for which diagnoses differed but age was consistent (i.e. apparent cross-sectional discordances), were evaluated. Discordances between AD and non-AD dementia status were resolved on the basis of pathology (cf. above) or flagged as discordant if no pathology data was available. Discordances between CN and AD status, or CN and non-AD dementia status, were resolved as respectively AD or non-AD dementia when those dementia diagnoses corresponded to a unique age-at-onset (of symptoms) without other available age information (i.e. indicating that a conversion likely occurred after the subject was lost to follow-up in the cohort that last observed a CN status), or, were flagged as discordant if duplicate entries shared the same age-at-examination and age-at-last-exam. Discordances between CN and MCI status, or MCI and AD status, or MCI and non-AD dementia status, were resolved as respectively MCI, AD, or non-AD dementia (i.e. keeping the most severe diagnosis).

Finally, once all clinical diagnostic and pathological data were unified across duplicate entries, pathological criteria were applied once more to obtain the final diagnoses. Where possible, AD diagnoses were verified by neuropathology as middle or high AD likelihood following NIA-Reagan 1997 criteria (moderate to frequent neuritic plaques and Braak stage III-VI)[14]. In concordance with the category “possible AD dementia with evidence of the AD pathophysiological process” from the NIA-AA 2011 criteria[17], we attributed possible AD diagnoses to subjects who met clinical criteria for non-AD dementia but also met AD neuropathological criteria. In concordance with the NIA-AA 2011/2012 framework[18,19], we also evaluated neuropathology in MCI subjects to verify presumed AD etiology and considered subjects as cases if AD pathology, following NIA-Reagan 1997 criteria (cf. above), was present (i.e. marking high likelihood of AD etiology). Controls were not re-evaluated based on neuropathology data.

Beyond cross-referencing clinical diagnostic and pathological data across subjects, other covariates were considered for cross-referencing or sharing in case of missingness across duplicate entries. These included age-at-onset of cognitive symptoms, age-at-examination providing clinical diagnosis, at-at-last exam, age-at-death, sex, race, ethnicity, *APOE* genotype provided from demographics, *APOE* genotype provided from whole-genome sequencing, and *APOE* genotype provided from whole-exome sequencing. Duplicate entries with discordant sex or race information were flagged for exclusion.

Genetic Data Quality Control and Processing

Discovery Samples Genetic Data Harmonization and Standard Quality Control

Genotypes were available from commercial high-density single-nucleotide polymorphism (SNP) genotyping microarrays (Illumina or Affymetrix), Whole-exome sequencing (WES), or Whole-genome sequencing (WGS) (**Table S1**). Genotype samples had their genetic variants lifted to hg19 using liftOver if not released in hg19[28]. Autosomal variants were extracted from the SNP array data and further processed in several stages. First, SNP array data were processed by the Genotype Harmonizer with CEU and TSI HapMap populations as the reference panel, to perform automatic strand alignment[29]. Then, multi-allelic SNPs, SNPs located on common copy number or segmental duplication regions, and duplicated or monomorphic SNPs, were removed. The list of multi-allelic SNPs or SNPs located on common copy number and segmental duplication regions was created using Tri-Typer[30]. The list of CNV and segmental duplication regions was curated from the Eichler lab (eichlerlab.gs.washington.edu/database.html)[31] and the gnomAD website (gnomad.broadinstitute.org/downloads)[32]. All respective genotype data sets were then iteratively merged with each other, applying strand flipping and variant ID updating as applicable, to ultimately obtain parsimonious data sets that could be merged for cross-sample relationship determination and principal component analyses (cf. below).

Genetic data were then further processed using Plink v1.9. The numbers of remaining samples after each quality control (QC) or processing step are listed in **Table S2**. For each sample platform, subjects with autosome missingness (≥ 5%) and sex problems (discordance between genetic sex and demographic sex, or deviation of expected X-chromosome homozygosity/heterozygosity) were flagged for exclusion.

Discovery Samples Ancestry Determination

Individual ancestries were determined using SNPweights v.2.1 with populations from the 1000 Genomes Consortium as a reference[33,34]. By applying an ancestry percentage cut-off ≥ 75%, the samples were stratified into the five super populations, South-Asians (SAS), East-Asians (EAS), Americans (AMR), Africans (AFR) and Europeans (EUR) (**Figure S1**). Subjects with a genetic ancestry that differed from their race, as provided in cohort demographics, were flagged for exclusion.

Discovery Samples Relationship Determination using Plink

Across all cohorts (and ethnicities) the relatedness of subjects (after QC indicated above) was evaluated through identity-by-descent (IBD) analysis (using directly genotyped non-palindromic SNPs that were

shared across all genetic datasets with a call rate > 99%, minor allele frequency (MAF) > 1%). This IBD outcome (**Figure S3-A**) was used for duplicate (IBD > 0.95) tracking across samples.

Discovery Samples Relationship Determination and Principal Component Analysis using GENESIS

Across SNP array and WGS data (or SNP array, WES, and WGS data for duplicate identification), the relatedness of subjects and principal components capturing population substructure were determined using IBD and principal component analyses (PCA) as implemented through the R package GENESIS (R v3.6.0)[35]. Specifically, this approach first uses an R-implementation of KING-robust to determine kinship coefficients that take into account ancestry divergence. The derived pairwise kinship coefficients are then used to perform a PCA in related samples (PC-AiR) providing accurate ancestry inference not confounded by family structure. The latter output is then used to estimate kinship coefficients using PC-Relate, which accounts for population structure (ancestry) among sample individuals through the use of ancestry representative principal components (PCs) to provide accurate relatedness estimates due only to recent family (pedigree) structure. For each respective data merge, these analyses were performed on directly genotyped and pruned SNPs (R^2 < 0.5, call rate > 99.9%, MAF > 1%, and excluding palindromic SNPs) in non-Hispanic white European ancestry individuals (**Figure S2-3**).

Discovery Samples APOE genotype assessment in old and new ADSP WES/WGS

The “old” ASDP WES (N=10,919) and ADSP WGS (N=4,750), which were available to us prior to 2020, refer to the ADSP discovery and extension phases described in detail in the main text. These data were used for variant association analyses. The new ADSP WES (N=20,503) and ADSP WGS (N=16,906), which were available to us as of 2021, refer to updated samples (including the prior ADSP data and more) and updated joint calling performed by the ADSP (NG00067.v5)[36]. These data were used to verify *APOE* genotypes (and filter discordant subjects) in variant association analyses for approach 2.

In both the old and new ADSP WGS, rs429358 and rs7412 showed low genotype missingness across subjects, reflecting good variant quality metrics in the joint calling performed by ADSP. In the old and new ADSP WES, rs7412 respectively did not pass ADSP quality control and showed a high genotype missingness at (32.5%). This resulted from a low read depth and genotype quality in some of the different WES capture kits that were used in the ADSP WES[2]. We therefore sought to re-call both variants in order to fill out missing *APOE* information where possible. We first inferred the variants' genotype using data called by the ADSP, which required a read depth read depth (DP) >=10 and genotype quality (GQ) >= 20. We then

further inferred the variants' genotype if DP and GQ were respectively greater than or equal to 6 and 20, observing at least 20% alternate allele reads to call a heterozygote (e.g. *APOE**3/4).

After this first round of *APOE* genotype ascertainment, some individuals still had either the rs7412 or rs429358 genotype missing (i.e., only one of the two variants could be called using the above criteria), making it impossible to infer their *APOE* genotype from the ADSP NGS data alone. Many of these remaining individuals however had a reported *APOE* genotype in their demographics that could be used to complete the missing information in a second additional round of *APOE* genotype ascertainment. This approach was preferred over relying solely on the *APOE* genotype in the demographics, since the genotype calls on the ADSP NGS data are expected to provide higher accuracy compared to other commonly used *APOE* direct genotyping methods[37]. To illustrate, consider the example where one of these remaining individuals in the sequencing data was homozygous for the reference allele at rs429358, which would suggest the subject is *APOE**3/3, but had a missing genotype at rs7412. In this case, from the ADSP NGS data, we know that this individual is not carrying an *APOE**4 allele, but we cannot determine the presence or absence of an *APOE**2 allele. We then turned to the information from the *APOE* genotype provided in the demographics to infer the most likely *APOE* genotype. For the current example, if the individual has a provided *APOE* genotype that was 2/2, 2/3, or 3/3, then the information in the ADSP NGS data is deemed concordant with the provided *APOE* genotype (that is, rs429358 is always the reference allele for those provided *APOE* genotypes) and we used the provided *APOE* genotype. However, if the provided *APOE* genotype was 4/4 or 3/4, then we would correct it to *APOE**3/3, because the ADSP NGS information clearly indicated there was no *APOE**4 genotype call (similarly a provided *APOE**2/4 genotype would be corrected to *APOE**2/3). This can be generalized as: for remaining individuals with DP>=6 and GQ>=20 at rs429358, the ADSP NGS data at rs429358 was used to change, when discordant, the provided *APOE**3 genotype to *APOE**4, or vice-versa. One additional extension to this step was implemented for the few scenarios where the ADSP NGS data called two rs429358 alleles (i.e. *APOE**4/4) but the allelic distribution indicated that the reference allele was still observed (e.g. 1 REF allele and 7 ALT alleles). In these situations, if the provided *APOE* genotype indicated the presence of *APOE**3, then the genotype was corrected to *APOE**3/4 (reasoning there is sufficient evidence to support the presence of an *APOE**3 genotype). The extra checks described in this paragraph were also applied to subjects in the first QC round (prior paragraph), who had 6<=DP<10 and GQ>=20 for both rs429358 and rs7412.

As a quality check, using these thresholds, we did not observe any discordance in the inferred *APOE* genotype across 3,499 duplicates between the ADSP WGS and ADSP WES.

Statistical Analyses – Additional Model Criteria

Case-Control Analyses

For age adjustment, when multiple age data were available, we prioritized in cases age-at-onset of symptoms (AAO) > age-at-examination providing clinical diagnosis of AD (AAE) > age-at-death (AAD), and in controls AAD > age-at-last-examination (AAL) (**Table S3**). This priority ranking is consistent with prior AD studies[1,38]. For cases that only had AAD available, the final ages used for regression analysis were subtracted by 10 years in order to approximate AAO. This reflects expected mean delays between AAO and AAD for AD patients[39], and is consistent with the derived age covariate for AD case-control analyses provided by the Alzheimer's Disease Genetics Consortium (ADGC) on NIAGADS[40]. In the ADNI and ROSMAP cohorts, which provide longitudinal diagnoses with matching age information, but do not directly provide AAO, the AAE variable used either the age-at-MCI-diagnosis or the first age-at-dementia-diagnosis.

Models including unrelated individuals

In sensitivity analyses that did not include related individuals, only a single subject was retained per relatedness cluster, prioritizing first younger cases followed by older controls.

Figure S1. Admixture plot for the five major super populations across all discovery samples. Black vertical line marks the cut-off for EUR ancestry [>=75%].

Abbreviations: EUR, European; AFR, African; AMR; American; SAS, Southern Asian; EAS; Eastern Asian.

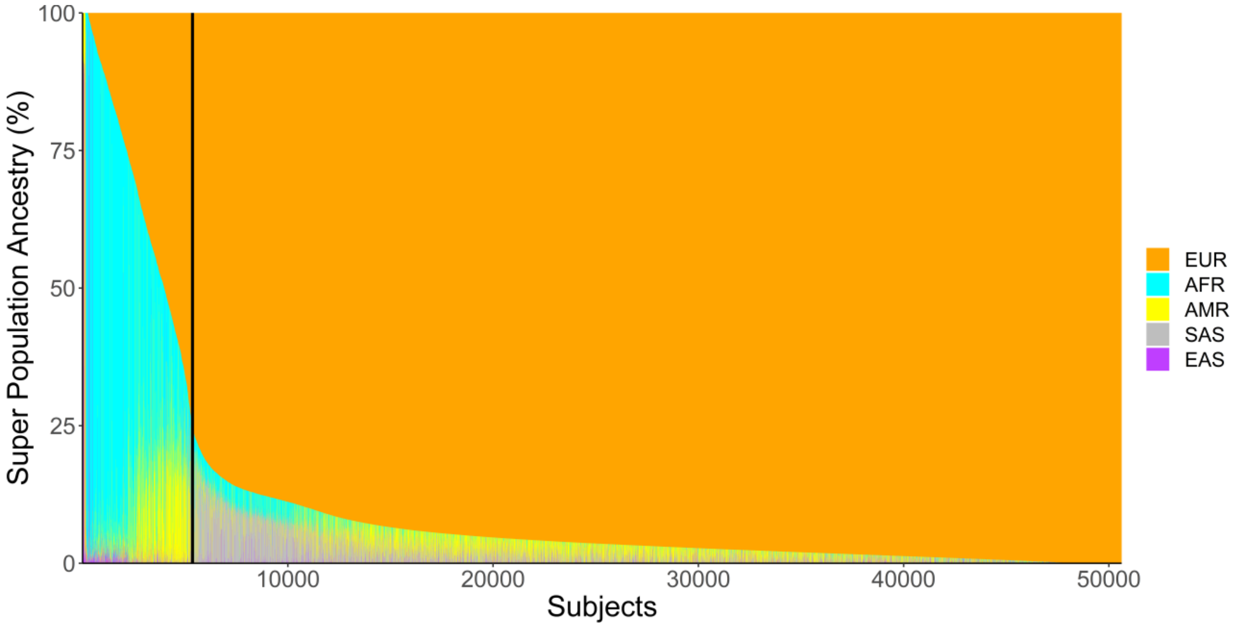

Figure S2. First five principal components of the genetic population structure in European subjects from the discovery samples. (A) PCs are labelled by sub-European ancestries for merged SNP array and WGS data.

Abbreviations: PC, principal component; EU, European; NWE, Northwestern European; SEE, Southeastern European; AJE, Ashkenazi Jewish.

A

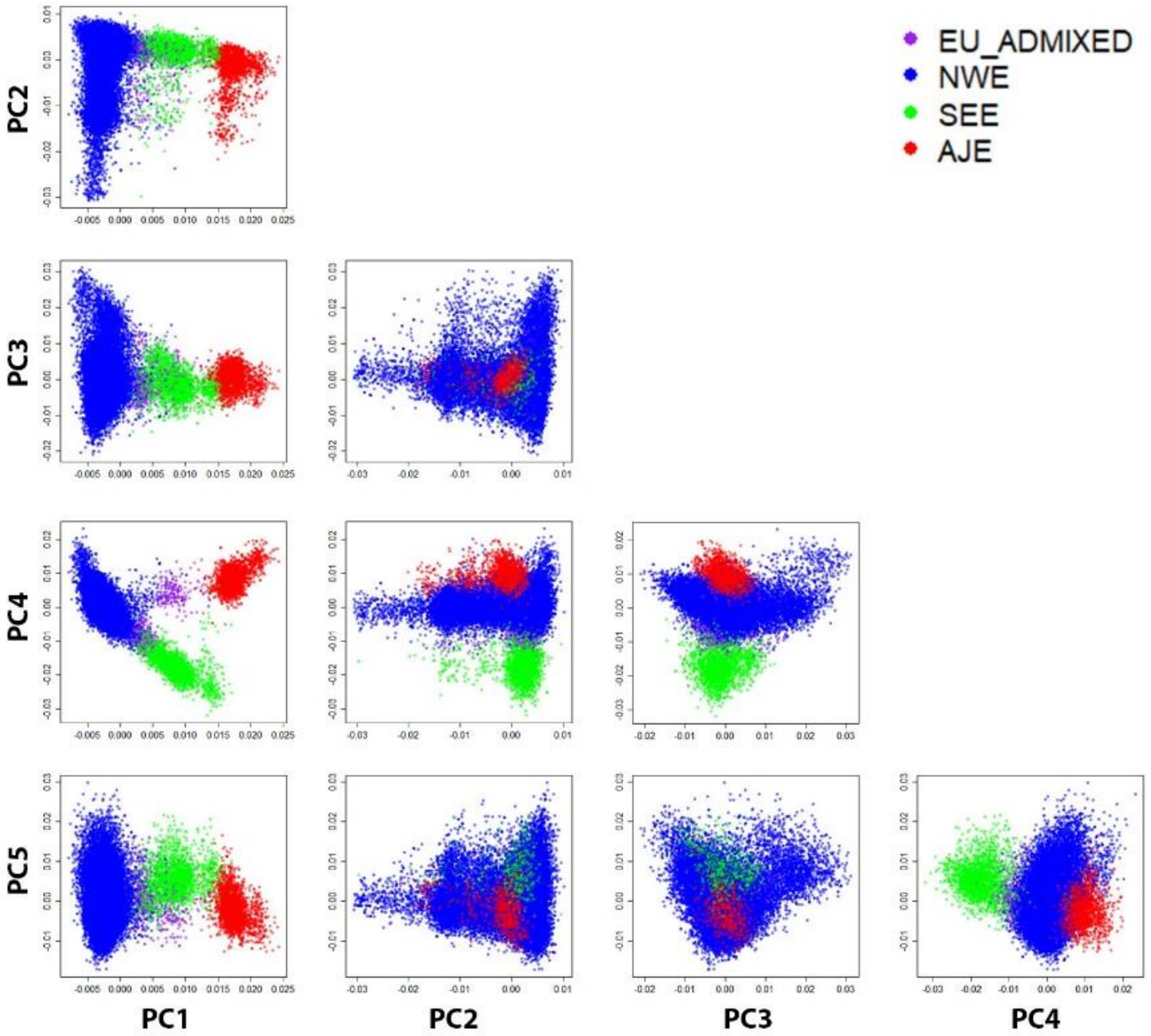

Figure S2. **(B)** PCs are labelled by genetic source for merged SNP array and WGS data.

Abbreviations: PC, principal component; SNP, single nucleotide polymorphism; WGS, whole genome sequencing; ADSP, Alzheimer's Disease Sequencing Project.

B

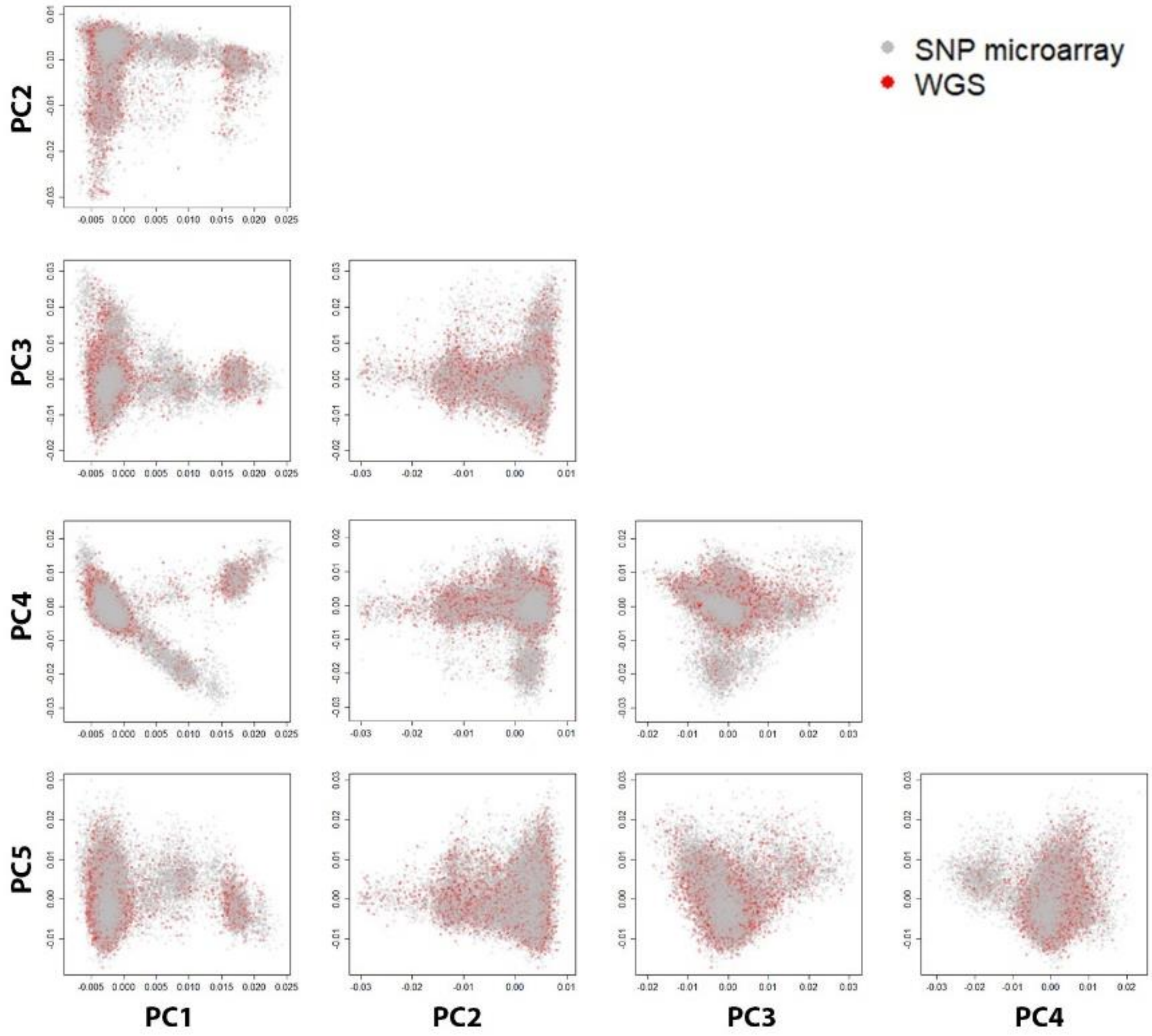

Figure S2. (C) PCs are labelled by Diagnosis. The Diagnosis of “Other” refers to subjects with diagnoses such as “demented not due to AD” or “mild cognitive impairment”.

Abbreviations: PC, principal component; CN, Cognitively normal; AD, Alzheimer’s disease.

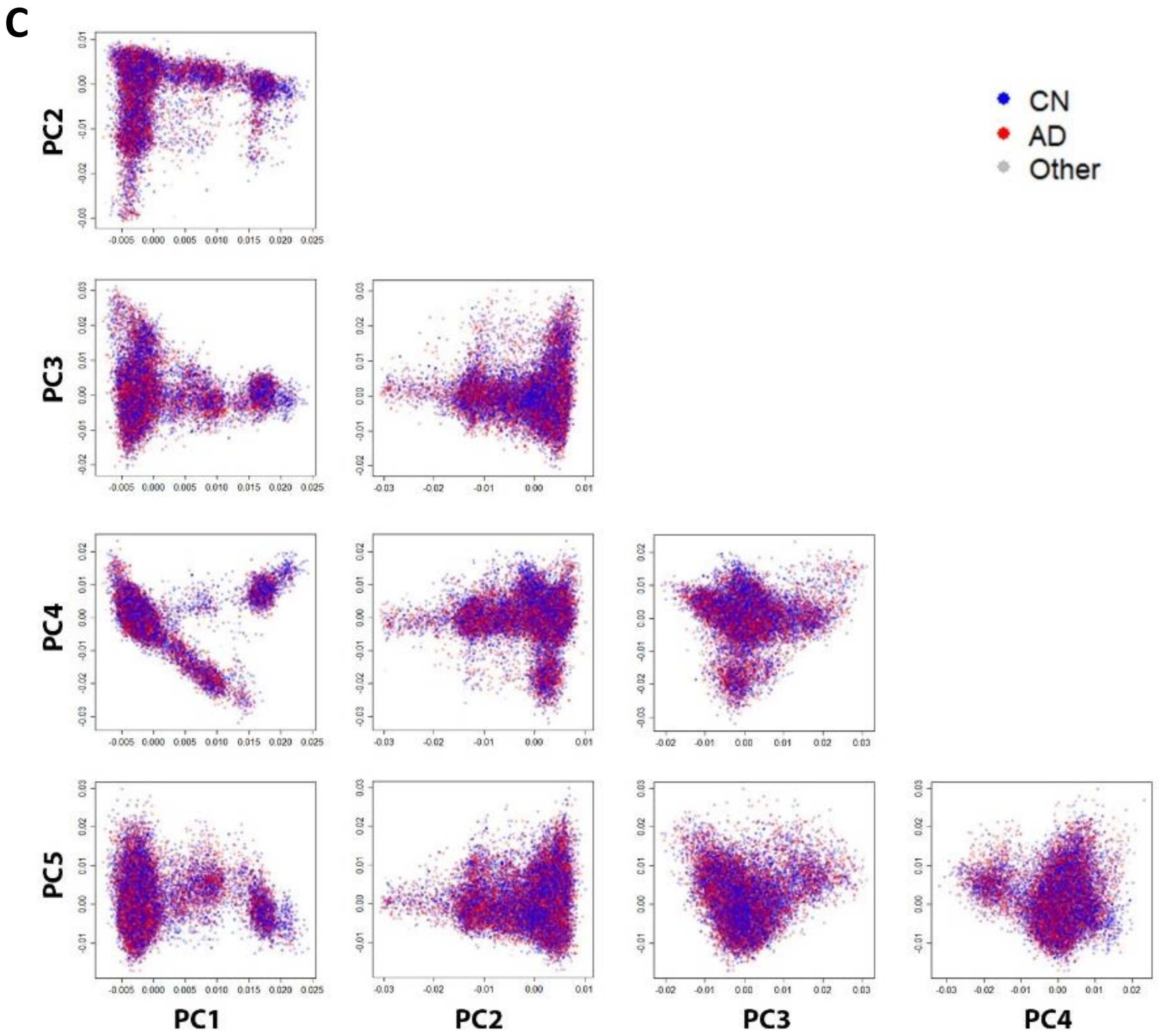

Figure S3. Identity-by-descent (IBD) analyses. Panel shows scatter a density plot, with values ranging from 0 (blue) to 200 or more (red) IBD pairs. Dashed lines indicate boundaries between levels of relatedness. All IBD analyses were thresholded at the lower boundary of 4th degree relatedness (using KING-robust and PC-Air/PC-Relate, the expected reliable lower boundary for dense SNP data is 3rd or 4th degree relatedness[35]). The panel shows an IBD analyses performed on a merge of SNP array and WGS data using KING-robust and PC-Air/PC-Relate. X-axis shows k0, indicating the probability of having 0 alleles identical by descent (ranging 0 to 1). Y-axis shows kin, the pairwise kinship coefficient estimate (ranging 0 to 0.5 conventionally, but possibly extending beyond these boundaries given the use of ancestry divergence measures by KING and PC-Relate). Note the well-conditioned relationship of k0 and kin down to the lower boundary of 3rd degree relatedness. High intensity inflation was visible at the lower boundary of 4th degree relatedness, suggesting inflated number of IBD pairs. Relatedness levels in this approach were thus judged to be reasonably reliable down to 3rd degree relatedness.

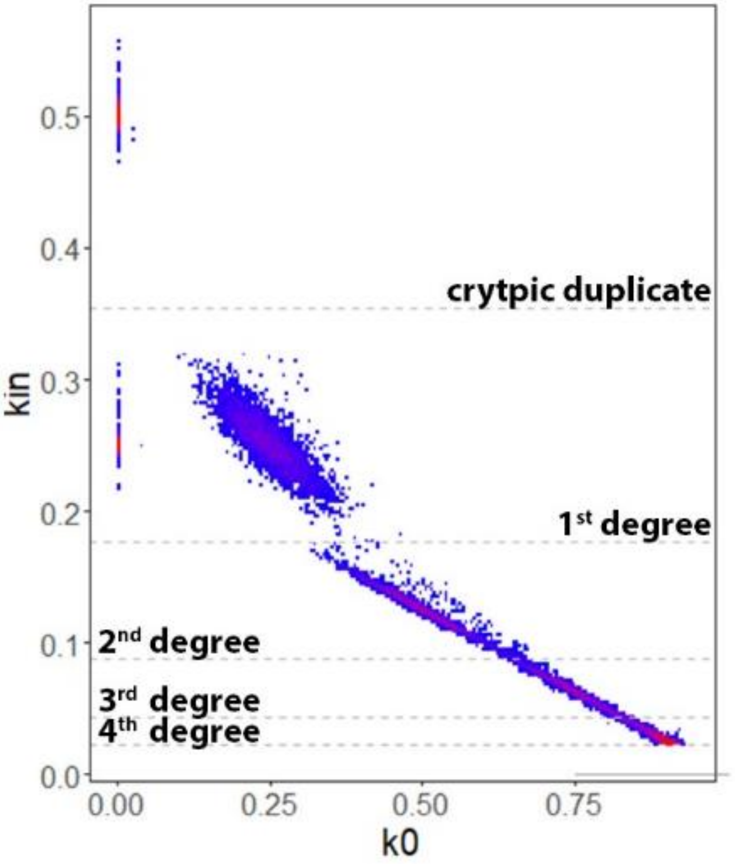

Figure S4. Genotype intensity data in EADB for rs439401. Top panel shows full sample, bottom panel shows *APOE**4/4 subjects.

Abbreviations: GT, genotype.

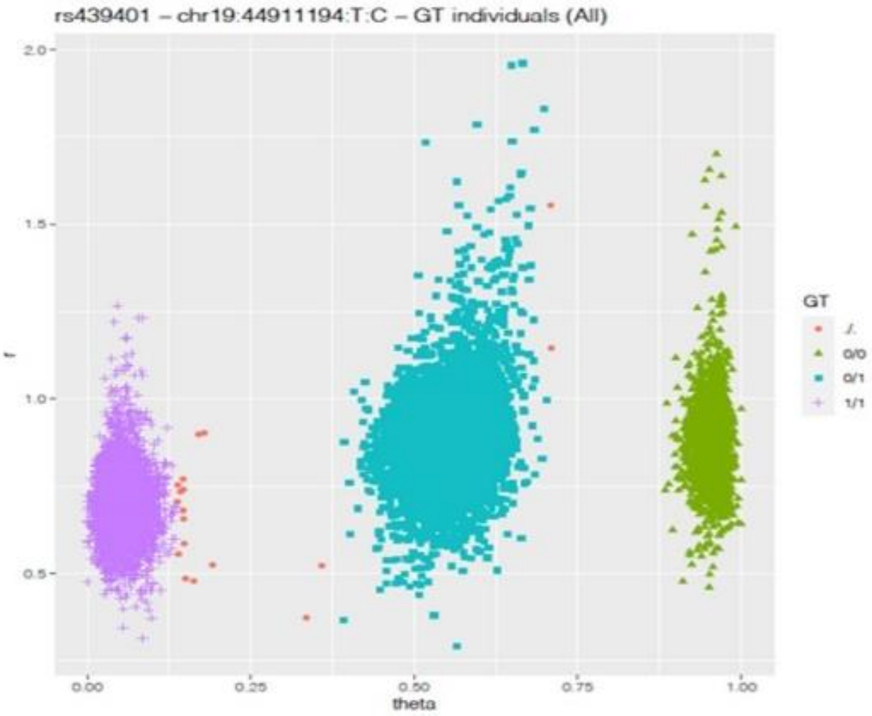

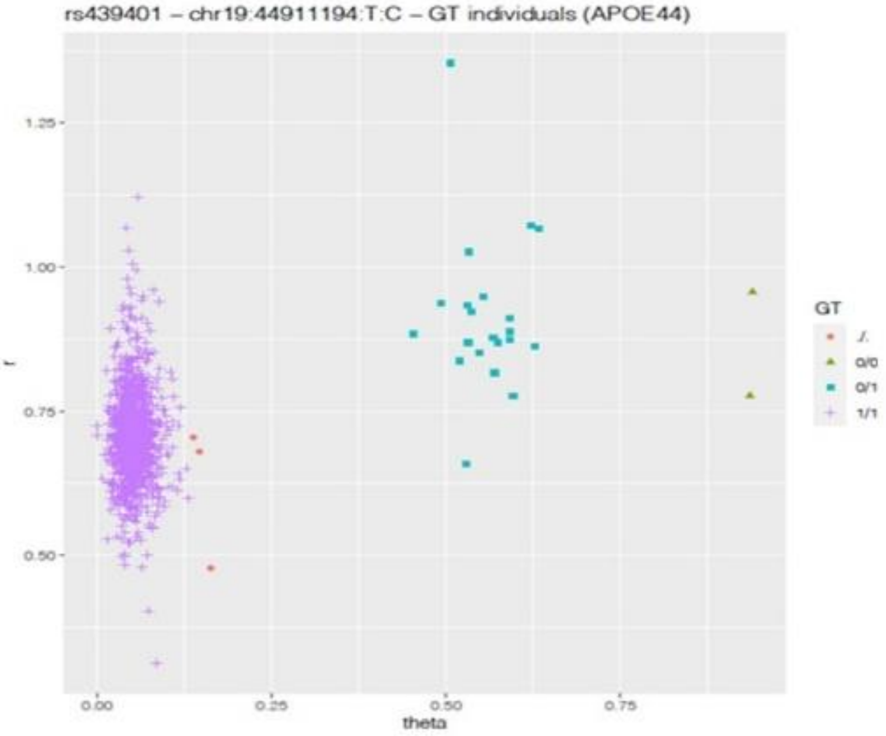

Figure S5. Discovery case-control association results for rs439401 when in-phase with *APOE4, comparing the basic model to cohort and cohort/array/center adjustment, using *APOE* filtering approach 1.**

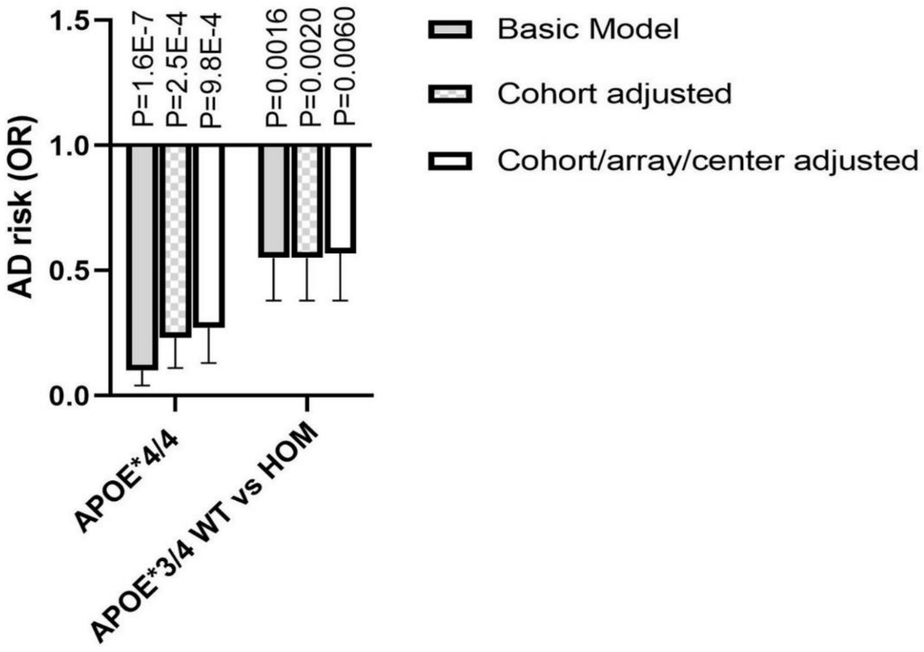

Figure S6. Simulation of the concordance between observed *APOE4/4 and true *APOE**4/4 genotypes considering different type I and II error rates. A)** Controls. **B)** Cases. Four different true frequencies were considered for controls and cases respectively. The frequencies were centered around the frequency observed in the full discovery cohort, which is somewhere around the expected true frequency. Type I error rate is defined as the probability to mis-classify non-*APOE**4/4 as *APOE**4/4; Type II error rate is defined as the probability to mis-classify *APOE**4/4 as non-*APOE**4/4.

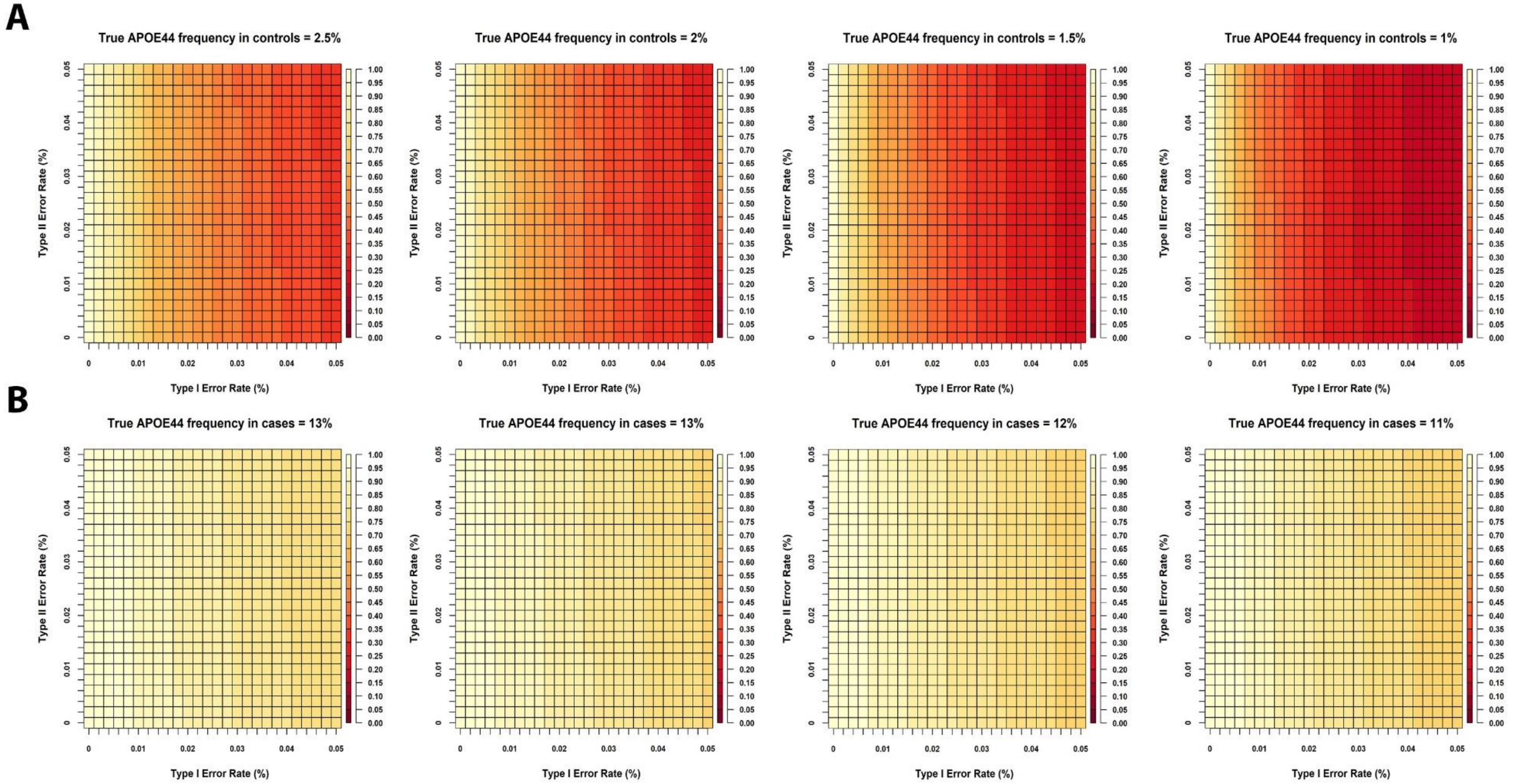

Figure S7. Simulation of the concordance between observed *APOE4/4 and true *APOE**4/4 genotypes considering different type I and II error rates and considering only *APOE**3/4 and *APOE**4/4 carriers. A)** Controls. **B)** Cases. One limitation when considering concordance rates as depicted in Figure S9 is that this is assumes each *APOE* genotype (2/2, 2/3, 3/3, 2/4, 3/4, 4/4) could equally produce any other *APOE* genotype when error occurs. Based on observations in Table S18, it may be more likely that *APOE**4/4 subjects are enriched for miscalled *APOE**3/4 subjects. Observed versus true *APOE**4/4 concordance rates may thus be better assessed by considering the true frequency of APOE*4/4 subjects with regard to the group of *APOE**3/4 and *APOE**4/4 subjects.

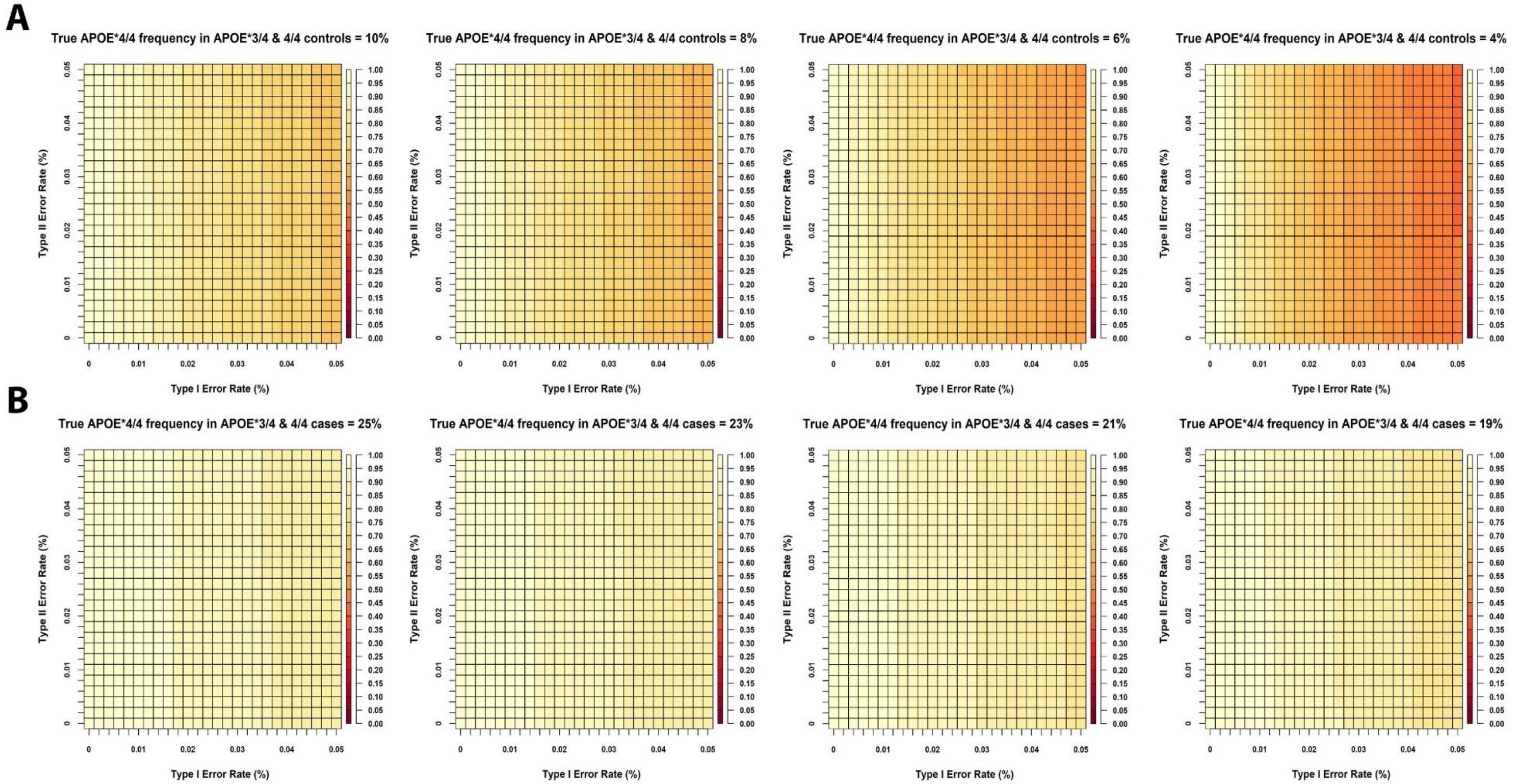

Figure S8. Concordance rates between imputed and provided *APOE* across different *APOE* strata. A) Combining cases and controls per stratum. **B)** Splitting cases and controls per stratum. Considered samples were those used in the discovery analyses, passing sample QC, and here further excluding subjects that did not have imputed genotypes available. Further, the analyses considered the provided *APOE* genotype to stratify samples as depicted (not using verification from the WGS/WES *APOE* genotypes). Number of subjects across groups are shown at the top of bar graphs. *Abbrevations: CN, Cognitively normal; AD, Alzheimer's Disease.*

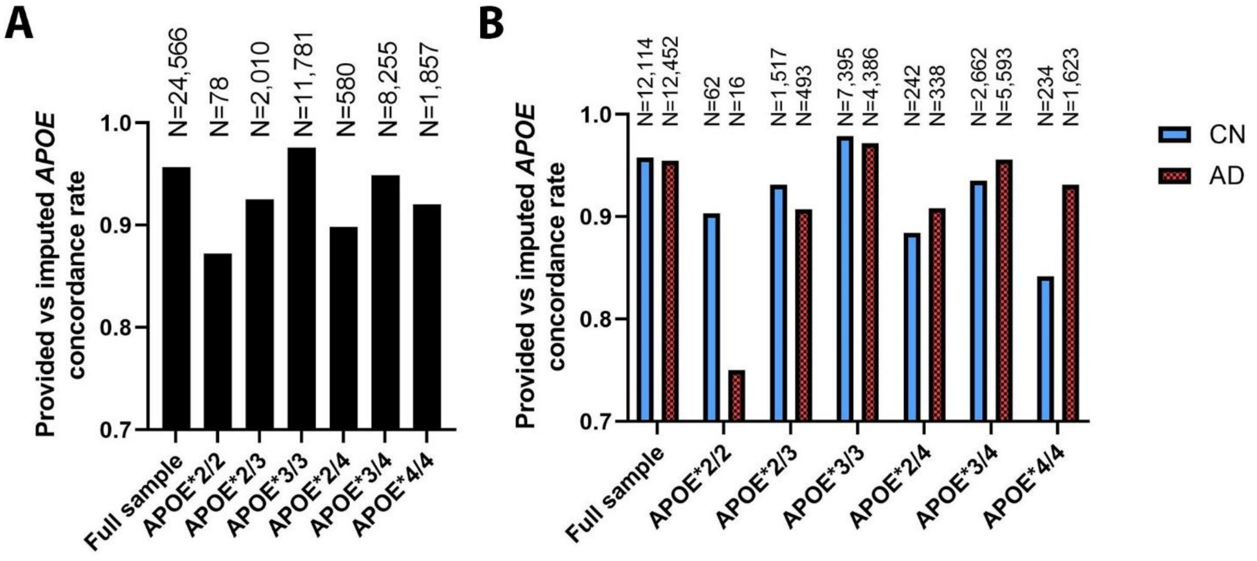

Table S1. Overview genotyping platforms across cohorts/projects considered in analysis approach 1.

Cohort/Project	Genotyping Platform	In-house data ID	Sample count	Data Repository & ID
ACT	Illumina Human 660W-Quad	ACT	2790	NIAGADS (NG00034) / dbGaP (phs000234)
ADC1	Illumina Human 660W-Quad	ADC1	2731	NIAGADS (NG00022) / NACC
ADC2	Illumina Human 660W-Quad	ADC2	928	NIAGADS (NG00023) / NACC
ADC3	Illumina Human OmniExpress	ADC3	1526	NIAGADS (NG00024) / NACC
ADC4	Illumina Human OmniExpress	ADC4	1054	NIAGADS (NG00068) / NACC
ADC5	Illumina Human OmniExpress	ADC5	1224	NIAGADS (NG00069) / NACC
ADC6	Illumina Human OmniExpress	ADC6	1333	NIAGADS (NG00070) / NACC
ADC7	Illumina Infinium Human OmniExpressExome	ADC7	1462	NIAGADS (NG00071) / NACC
ADDNEUROMED	Illumina Human 610-Quad	ADM_Q	315	Synapse AddNeuroMed (syn4907804)
	Illumina Human OmniExpress	ADM_O	329	Synapse AddNeuroMed (syn4907804)
ADNI	Illumina Human 610-Quad	ADNI_1	757	LONI ADNI
	Illumina Human OmniExpress	ADNI_2	361	LONI ADNI
	Illumina Omni 2.5	ADNI_O25	812	LONI ADNI
	Whole Genome Sequencing - Illumina	ADNI_WGS	812	LONI ADNI
ADNI-DOD	Illumina Human OmniExpress	ADNI_DOD	204	LONI ADNIDOD
ADSP discovery	Whole Exome Sequencing (discovery joint called) - Baylor College	ADSP_DISC_Baylor	2609	dbGaP (phs000572) / NACC
	Whole Exome Sequencing (discovery joint called) - Broad Institute	ADSP_DISC_Broad	4574	dbGaP (phs000572) / NACC
	Whole Exome Sequencing (discovery joint called) - Washington University	ADSP_DISC_Washu	3726	dbGaP (phs000572) / NACC
	Whole Exome Sequencing (discovery joint called) - unspecified	ADSP_DISC	10	dbGaP (phs000572) / NACC
ADSP extension	Whole Genome Sequencing (extension joint called) - Baylor College	ADSP_EXT_Baylor	1260	NIAGADS (NG00067) / NACC
	Whole Genome Sequencing (extension joint called) - Broad Institute	ADSP_EXT_Broad	1365	NIAGADS (NG00067) / NACC
	Whole Genome Sequencing (extension joint called) - Illumina	ADSP_EXT_Illumina	809	NIAGADS (NG00067) / NACC
	Whole Genome Sequencing (extension joint called) - Washington University	ADSP_EXT_Washu	1316	NIAGADS (NG00067) / NACC
EADI	Illumina Human 610-Quad	EADI	9863	contact: jean-charles.lambert@pasteur-lille.fr
EADB	Illumina Global Screening Array	EADB	64704	contact: jean-charles.lambert@pasteur-lille.fr

GenADA	Affymetrix 500K	GSK	1571	dbGaP (phs000219)
NIA-LOAD	Illumina Human 610-Quad	LOAD	5220	NIAGADS (NG00020)
MAYO	Illumina Human Hap300	MAYO_1	2099	Synapse AMP-AD (syn5591675) / NIAGADS (NG00029)
	Whole Genome Sequencing (AMP-AD joint called ROSMAP/MAYO/MSBB)	AMP_AD_MAYO_WGS	349	Synapse AMP-AD (syn22264775)
MAYO2	Illumina Omni 2.5	MAYO_2	314	Synapse AMP-AD (syn5550404)
	Whole Genome Sequencing (AMP-AD joint called ROSMAP/MAYO/MSBB)	AMP_AD_MAYO_WGS	349	Synapse AMP-AD (syn22264775)
MIRAGE	Illumina Human CNV370-Duo	MIRAGE_370	397	NIAGADS (NG00031)
	Illumina Human 610-Quad	MIRAGE_610	1105	NIAGADS (NG00031)
MSBB	Whole Genome Sequencing (AMP-AD joint called ROSMAP/MAYO/MSBB)	AMP_AD_MSBB_WGS	349	Synapse AMP-AD (syn3159438, syn22264775)
MTC	Illumina Human OmniExpress	MTC	542	NIAGADS (NG00096)
OHSU	Illumina Human CNV370-Duo	OHSU	647	NIAGADS (NG00017)
ROTTERDAM	Illumina Infinium II HumanHap550chip v3.0	ROTTERDAM	10150	contact: m.a.ikram@erasmusmc.nl
ROSMAP	Affymetrix GeneChip 6.0 - Broad Institute	ROSMAP_1B	1126	RADC Rush (contact:Gregory_Klein@rush.edu) / Synapse AMP-AD (syn3219045)
	Affymetrix GeneChip 6.0 - TGen	ROSMAP_1T	582	RADC Rush (contact:Gregory_Klein@rush.edu) / Synapse AMP-AD (syn3219045)
	Illumina Human OmniExpress 12 - Chop	ROSMAP_2C	382	RADC Rush (contact:Gregory_Klein@rush.edu) / Synapse AMP-AD (syn7824841)
	Illumina Multi-Ethnic - BU	ROSMAP_3BU	494	RADC Rush (contact:Gregory_Klein@rush.edu)
	Whole Genome Sequencing (AMP-AD joint called ROSMAP/MAYO/MSBB)	AMP_AD_ROSMAP_WGS	1196	RADC Rush (contact:Gregory_Klein@rush.edu) / Synapse AMP-AD (syn22264775)
TARCC	Affymetrix 6.0	TARCC	625	NIAGADS (NG00097)
TGEN2	Affymetrix 6.0	TGEN	1599	NIAGADS (NG00028)
UPITT	Illumina Human Omni1-Quad	UPITT	2440	NIAGADS (NG00026)
UM/VU/MSSM	Illumina Human 1M-Duo, Illumina 1M	UVM_A	1153	NIAGADS (NG00042)
	Affymetrix 6.0	UVM_B	864	NIAGADS (NG00042)
	Illumina Human 550K. Illumina Human 610-Quad	UVM_C	445	NIAGADS (NG00042)
WASHU	Illumina Human 610-Quad	WASHU_1	670	NIAGADS (NG00030)
WASHU2	Illumina Human OmniExpress	WASHU_2	235	NIAGADS (NG00087)
WHICAP	Illumina Human OmniExpress	WHICAP	647	NIAGADS (NG00093)

Table S2. Discovery sample sizes per cohort/project after sequential quality control and filtering steps (detailed in column titles). Cohorts/Arrays that had no coverage of rs439401 (genotyping rate 0%) are not listed.

**Cohorts**	**1. All genotyped subjects**	**2. Genotype missingness <95%**	**3. No sex problems/missingness**	**4. No ancestry/race discordance**	**5. No duplicate discordance**	**6. Filter to CN/AD**	**7. *APOE* genotype available**	**8. AGE available**	**9. AGE 60y and up**	**10. European (EU)**	**11. Retain unique non-duplicate**
ACT	2790	2790	2790	2786	2784	2558	2364	2364	2363	2270	2269
ADC1	2731	2731	2730	2697	2682	2522	2508	2508	2221	2058	2053
ADC2	928	928	927	927	923	829	827	827	820	817	816
ADC3	1526	1526	1525	1524	1519	1356	1354	1354	1253	1232	1232
ADC4	1054	1054	1054	1054	1054	875	871	871	839	828	827
ADC5	1224	1224	1223	1222	1221	1005	1005	1005	996	993	989
ADC6	1333	1333	1332	1332	1330	933	927	927	719	719	716
ADC7	1462	1462	1462	1460	1460	1314	1314	1314	1313	1308	1308
ADM_O	315	315	313	313	313	240	235	235	232	231	231
ADM_Q	329	329	281	281	281	202	201	201	196	196	196
ADNI_1	757	756	755	751	750	551	549	549	538	495	293
ADNI_2	361	361	359	353	353	248	248	248	241	206	185
ADNI_O25	812	812	811	804	804	470	470	470	462	433	362
ADNI_DOD	204	204	203	200	200	94	94	94	94	79	79
ADSP_EXT_Baylor	1260	1259	1259	1253	1251	1064	1064	1064	1033	119	119
ADSP_EXT_Broad	1365	1362	1362	1356	1352	1274	1274	1274	1245	972	291
ADSP_EXT_Illumina	809	809	808	801	801	468	468	468	460	432	1
ADSP_EXT_WashU	1316	1316	1315	1301	1298	1248	1248	1247	1204	144	32
LOAD	5220	5220	5201	5164	5155	4482	4470	4470	3940	3344	3050
MAYO_1	2099	2058	2058	2058	2056	1953	1918	1918	1917	1887	1761
MAYO_2	314	314	312	312	311	223	223	223	210	208	185
AMP_AD_MAYO_WGS	349	349	349	349	348	263	263	263	250	250	45
MIRAGE_370	397	397	358	358	358	356	331	329	321	295	276
MIRAGE_610	1105	1105	992	991	990	984	926	915	901	857	822
AMP_AD_MSBB_WGS	349	349	340	337	334	224	224	224	216	186	180
MTC	542	542	540	539	539	460	371	367	365	352	332
OHSU	647	641	634	634	618	576	561	561	559	554	511
ROSMAP_2C	382	382	380	380	380	298	298	298	298	293	261
ROSMAP_3BU	494	488	480	452	449	353	303	303	303	231	180
AMP_AD_ROSMAP_WGS	1196	1196	1185	1184	1167	937	937	937	937	937	647
UPITT	2440	2371	2364	2364	2364	2235	2227	2218	2218	2204	2163
UVM_A	1153	1153	1153	1153	1151	1147	1140	1140	1140	1137	1099
UVM_B	864	864	863	863	861	816	804	800	795	791	553
WASHU	670	670	668	668	667	640	629	599	582	582	434
WHICAP	647	647	647	647	647	639	638	638	638	631	622
Total	39444	39317	39033	38868	38771	33837	33284	33223	31819	28271	25120

Table S3. Expanded sample demographics for Alzheimer’s disease case-control association analyses.

Cohort		Diagnosis		Pathology		Sex	Age	Age type			
**Name**	**Participants after QC (N)**	**Type**	**(N)**	**Available (N (%))**	**AD Path. (N (%))**	**Female (N (%))**	**Age (Mean (SD))**	**AAD (Mean (SD)) [%]**	**AAL (Mean (SD)) [%]**	**AAE (Mean (SD)) [%]**	**AAO (Mean (SD)) [%]**
**DISCOVERY SAMPLE**											
**ACT**	2269	CN	1645	196 (11.9 %)	0 (0.0 %)	891 (54.2 %)	83.5 (5.9)	83.7 (5.7) [59.4 %]	83.2 (6.3) [40.6 %]	-	-
		AD	624	119 (19.1 %)	119 (100 %)	405 (64.9 %)	81.8 (6.3)	-	-	82.6 (5.8) [7.9 %]	81.8 (6.3) [92.1 %]
**NACC - ADC1**	2053	CN	490	152 (31.0 %)	8 (5.3 %)	279 (56.9 %)	81.4 (9.1)	85.8 (8.5) [39.2 %]	78.6 (8.3) [60.8 %]	-	-
		AD	1563	1535 (98.2 %)	1535 (100 %)	857 (54.8 %)	73.0 (7.5)	83.5 (6.0) [1.6 %]	-	79.6 (8.5) [6.3 %]	72.6 (7.2) [92.1 %]
**NACC - ADC2**	816	CN	125	18 (14.4 %)	4 (22.2 %)	88 (70.4 %)	79.8 (9.1)	86.3 (8.3) [21.6 %]	78.0 (8.6) [78.4 %]	-	-
		AD	691	329 (47.6 %)	329 (100 %)	362 (52.4 %)	73.1 (7.0)	-	-	76.9 (6.9) [1.3 %]	73.1 (7.0) [98.7 %]
**NACC - ADC3**	1232	CN	490	42 (8.6 %)	5 (11.9 %)	305 (62.2 %)	79.3 (9.5)	88.3 (7.5) [20.4 %]	77.0 (8.6) [79.6 %]	-	-
		AD	742	483 (65.1 %)	483 (100 %)	410 (55.3 %)	74.7 (8.3)	83.5 (6.4) [0.3 %]	-	80.2 (8.8) [4.0 %]	74.5 (8.2) [95.7 %]
**NACC - ADC4**	827	CN	422	50 (11.8 %)	15 (30.0 %)	261 (61.8 %)	79.4 (8.6)	87.1 (7.8) [18.2 %]	77.6 (7.8) [81.8 %]	-	-
		AD	405	156 (38.5 %)	156 (100 %)	217 (53.6 %)	74.0 (7.3)	-		79.7 (12.4) [0.7 %]	74.0 (7.3) [99.3 %]
**NACC - ADC5**	989	CN	589	83 (14.1 %)	16 (19.3 %)	382 (64.9 %)	81.9 (8.8)	88.8 (6.7) [22.6 %]	79.8 (8.2) [77.4 %]	-	-
		AD	400	150 (37.5 %)	150 (100 %)	215 (53.8 %)	74.5 (7.9)	-	-	76 (-) [0.2 %]	74.5 (7.9) [99.8 %]
**NACC - ADC6**	716	CN	357	39 (10.9 %)	15 (38.5 %)	240 (67.2 %)	80.0 (8.9)	87.0 (8.7) [21.0 %]	78.1 (8.0) [79.0 %]	-	-
		AD	359	119 (33.1 %)	119 (100 %)	192 (53.5 %)	74.4 (7.9)	-	-	90 (-) [0.3 %]	74.4 (7.9) [99.7 %]
**NACC - ADC7**	1308	CN	772	39 (5.1 %)	4 (10.3 %)	497 (64.4 %)	77.7 (7.8)	84.0 (8.3) [9.3 %]	77.1 (7.4) [90.7 %]	-	-
		AD	536	121 (22.6 %)	121 (100 %)	284 (53.0 %)	72.9 (7.7)	-	-	-	72.9 (7.7) [100 %]
**ADDNEURO**	427	CN	182	0 (0.0%)	-	102 (56.0 %)	76.6 (6.3)	-	76.6 (6.3) [100 %]	-	-
		AD	245	0 (0.0 %)	-	158 (64.5 %)	73.4 (6.2)	-	-	78.2 (5.9) [8.6 %]	73.0 (6.0) [91.4 %]
**ADNI**	840	CN	344	1 (0.3 %)	0 (0.0 %)	172 (50.0 %)	78.5 (6.8)	84 (-) [0.3 %]	78.5 (6.8) [99.7 %]	-	-
		AD	496	29 (5.8 %)	29 (100 %)	211 (42.5 %)	75.5 (6.7)	-	-	75.6 (6.7) [97.6 %]	72.7 (7.5) [2.4 %]
**ADNI-DOD**	79	CN	79	0 (0.0 %)	-	0 (0.00 %)	70.2 (5.3)	-	70.2 (5.3) [100 %]	-	-
		AD	-	-	-	-	-	-	-	-	-
**ADSP Extension**	443	CN	160	26 (16.3 %)	1 (3.8 %)	93 (58.1 %)	79.1 (7.5)	82.7 (7.9) [5.6 %]	78.8 (7.5) [94.4 %]	-	-
		AD	283	201 (71.0 %)	201 (100 %)	152 (53.7 %)	74.2 (8.0)	-	-	73 (-) [0.4 %]	74.2 (8.0) [99.6 %]
**MAYO**	1761	CN	1048	132 (12.6 %)	0 (0.0 %)	543 (51.8 %)	74.2 (5.4)	82.2 (5.0) [0.6 %]	74.1 (5.3) [99.4 %]	-	-
		AD	713	227 (31.8 %)	227 (100 %)	414 (58.0 %)	73.5 (5.1)	-	-	73.5 (5.1) [99.4 %]	68.8 (3.9) [0.6 %]
**MAYO2**	230	CN	160	160 (100 %)	0 (0.0 %)	81 (50.6 %)	83.8 (7.1)	83.8 (7.1) [100 %]	-	-	-
		AD	70	70 (100 %)	70 (100 %)	48 (68.6 %)	74.6 (5.7)	84.4 (5.6) [98.6 %]	-	-	85 (-) [1.4 %]
**MIRAGE**	1098	CN	688	0 (0.0 %)	-	403 (58.6 %)	72.0 (7.2)	-	72.0 (7.2) [100 %]	-	-
		AD	410	0 (0.0 %)	-	254 (62.0 %)	70.9 (6.5)	-	-	74.5 (4.2) [1.0 %]	70.9 (6.5) [99.0 %]
**MSBB**	180	CN	31	31 (100 %)	2 (6.5 %)	23 (74.2 %)	82.4 (7.6)	82.4 (7.6) [100 %]	-	-	-
		AD	149	149 (100 %)	149 (100 %)	100 (67.1 %)	78.1 (8.4)	85.6 (7.1) [53.7 %]	-	-	80.9 (9.0) [46.3 %]
**MTC**	332	CN	113	0 (0.0 %)	-	70 (61.9 %)	73.1 (7.8)	-	73.1 (7.8) [100 %]	-	-
		AD	219	1 (0.5 %)	-	122 (55.7 %)	74.4 (7.3)	89 (-) [0.5 %]	-	81.0 (8.3) [10.5 %]	73.6 (6.8) [89.0 %]
**NIA-LOAD**	3050	CN	1303	69 (5.3 %)	6 (8.7 %)	788 (60.5 %)	74.5 (9.2)	85.4 (7.0) [7.1 %]	73.7 (8.8) [92.9 %]	-	-
		AD	1747	463 (26.5 %)	463 (100 %)	1145 (65.5 %)	73.8 (6.8)	-	-	84.2 (8.0) [0.5 %]	73.7 (6.8) [99.5 %]
**OHSU**	511	CN	326	326 (100 %)	77 (23.6 %)	171 (52.5 %)	85.8 (7.3)	85.8 (7.3) [100 %]	-	-	-
		AD	185	185 (100 %)	185 (100 %)	117 (63.2 %)	85.3 (6.1)	-	-	85.1 (6.5) [17.8 %]	85.3 (6.0) [82.2 %]

**ROSMAP**	1088	CN	528	291 (55.1 %)	123 (42.3 %)	383 (72.5 %)	86.0 (7.2)	88.1 (6.5) [58.5 %]	83.1 (7.1) [41.5 %]	-	-
		AD	560	502 (89.6 %)	502 (100 %)	412 (73.6 %)	84.4 (6.6)	-	-	84.2 (6.5) [86.8 %]	85.4 (7.0) [13.2 %]
**UM/VU/MSSM**	1652	CN	931	42 (4.5 %)	0 (0.0 %)	594 (63.8 %)	73.3 (7.7)	80.9 (9.1) [6.3 %]	72.8 (7.3) [93.7 %]	-	-
		AD	721	160 (22.2 %)	160 (100 %)	461 (63.9 %)	74.3 (7.3)	86.0 (7.1) [7.3 %]	-	85.7 (6.1) [2.6 %]	73.9 (7.1) [89.9 %]
**UPITT**	2163	CN	870	0 (0.0 %)	-	549 (63.1 %)	75.4 (6.0)	-	75.4 (6.0) [100 %]	-	-
		AD	1293	0 (0.0 %)	-	822 (63.6 %)	73.3 (6.6)	-	-	78.1 (7.8) [7.3 %]	72.9 (6.4) [92.7 %]
**WASHU**	434	CN	139	2 (1.4 %)	0 (0.0%)	87 (62.6 %)	76.9 (8.5)	90.5 (0.7) [1.4 %]	76.7 (8.4) [98.6 %]	-	-
		AD	295	28 (9.5 %)	28 (100 %)	171 (58.0 %)	76.5 (8.8)	-	-	-	76.5 (8.8) [100 %]
**WHICAP**	622	CN	548	0 (0.0 %)	-	330 (60.2 %)	81.8 (6.7)	-	81.8 (6.7) [100 %]	-	-
		AD	74	0 (0.0 %)	-	53 (71.6 %)	84.3 (7.7)	-	-	-	84.3 (7.7) [100 %]
**TOTAL**	**25120**	CN	12340	1699 (13.8 %)	276 (16.2 %)	7332 (59.4 %)	78.3 (8.6)	85.3 (7.1) [21.5 %]	76.5 (8.0) [78.5 %]	-	-
		AD	12780	5027 (39.3 %)	5027 (100 %)	7582 (59.3 %)	74.9 (7.8)	85.1 (6.6) [1.8 %]	-	77.8 (7.7) [16.2 %]	74.3 (7.7) [82.0 %]
**REPLICATION SAMPLES**											
**ROTTERDAM**	10150	CN	8824	-	-	4956 (56.2 %)	77.5 (9.5)	81.6 (8.8) [41.2 %]	74.7 (8.9) [58.8 %]	-	-
		AD	1326	-	-	945 (71.3 %)	83.7 (6.6)	-	-	83.7 (6.6) [100 %]	-
**EADI**	8571	CN	6502	-	-	3920 (60.3 %)	80.4 (6.6)	82.9 (6.3) [15.6 %]	79.9 (6.7) [84.4 %]	-	-
		AD	2069	-	-	1359 (65.7 %)	75.2 (8.2)	-	-	75.2 (8.2) [100 %]	-
**EADB**	21860	CN	12295	-	-	7155 (58.2 %)	73.3 (8.0)	79.7 (6.8) [0.2 %]	73.3 (8.0) [99.8 %]	-	-
		AD	9565	-	-	6055 (63.3 %)	74.9 (7.8)	-	-	-	74.9 (7.8) [100 %]

Subjects with a diagnosis of cognitively normal at their last examination prior to death were kept as controls, regardless of the presence of AD pathology.

Table S4. Expanded sample demographics for Alzheimer's disease case-control association analyses in evaluated *APOE4 carriers.**

Cohort				*APOE* *3/4 carriers			*APOE* *4/4 carriers		
Name	Participants after QC (N)	Diagnosis (N)		(N (%))	Female (N (%))	Age (Mean (SD))	(N (%))	Female (N (%))	Age (Mean (SD))
**DISCOVERY SAMPLES**									
**ACT**	2269	CN	1645	299 (18.2 %)	176 (58.9 %)	82.7 (6.2)	16 (1.0 %)	11 (68.8 %)	81.8 (6.8)
		AD	624	211 (33.8 %)	128 (60.7 %)	80.4 (5.9)	40 (6.4 %)	30 (75.0 %)	74.7 (8.0)
**NACC - ADC1**	2053	CN	490	99 (20.2 %)	51 (51.5 %)	77.2 (9.2)	5 (1.0 %)	4 (80.0 %)	71.8 (3.3)
		AD	1563	765 (49.0 %)	408 (53.3 %)	73.0 (7.1)	255 (16.3 %)	118 (46.3 %)	69.4 (5.9)
**NACC - ADC2**	816	CN	125	23 (18.4 %)	17 (73.9 %)	76.1 (9.3)	3 (2.4 %)	2 (66.7 %)	78.3 (8.5)
		AD	691	313 (45.3 %)	173 (55.3 %)	72.5 (6.3)	113 (16.4 %)	51 (45.1 %)	68.8 (5.4)
**NACC - ADC3**	1232	CN	490	94 (19.2 %)	61 (64.9 %)	76.0 (8.9)	7 (1.4 %)	4 (57.1 %)	70.9 (3.0)
		AD	742	339 (45.7 %)	182 (53.7 %)	73.5 (7.2)	96 (12.9 %)	54 (56.3 %)	70.8 (6.0)
**NACC - ADC4**	827	CN	422	91 (21.6 %)	54 (59.3 %)	76.8 (8.4)	11 (2.6 %)	7 (63.6 %)	77.0 (8.2)
		AD	405	166 (41.0 %)	95 (57.2 %)	72.7 (6.0)	46 (11.4 %)	24 (52.2 %)	68.4 (4.8)
**NACC - ADC5**	989	CN	589	105 (17.8 %)	66 (62.9 %)	79.9 (8.2)	10 (1.7 %)	6 (60.0 %)	75.2 (6.6)
		AD	400	185 (46.3 %)	108 (58.4 %)	73.4 (6.7)	48 (12.0 %)	25 (52.1 %)	68.6 (4.6)
**NACC - ADC6**	716	CN	357	84 (23.5 %)	55 (65.5 %)	78.2 (8.3)	5 (1.4 %)	3 (60.0 %)	72.4 (7.4)
		AD	359	156 (43.5 %)	85 (54.5 %)	72.5 (7.0)	38 (10.6 %)	17 (44.7 %)	69.0 (5.4)
**NACC - ADC7**	1308	CN	772	203 (26.3 %)	125 (61.6 %)	77.3 (7.7)	14 (1.8 %)	12 (85.7 %)	74.1 (6.4)
		AD	536	239 (44.6 %)	129 (54.0 %)	72.3 (6.6)	77 (14.4 %)	39 (50.6 %)	67.4 (6.7)
**ADDNEURO**	427	CN	182	37 (20.3 %)	22 (59.5 %)	75.5 (6.4)	3 (1.6 %)	1 (33.3 %)	73.3 (7.2)
		AD	245	102 (41.6 %)	71 (69.6 %)	72.8 (5.5)	27 (11.0 %)	19 (70.4 %)	68.9 (4.9)
**ADNI**	840	CN	344	82 (23.8 %)	44 (53.7 %)	76.4 (7.3)	7 (2.0 %)	2 (28.6 %)	77.3 (4.5)
		AD	496	237 (47.8 %)	102 (43.0 %)	75.1 (6.1)	83 (16.7 %)	33 (39.8 %)	72.1 (6.4)
**ADNI-DOD**	79	CN	79	16 (20.3 %)	0 (0.00 %)	70.0 (6.2)	2 (2.5 %)	0 (0.00 %)	72.5 (3.5)
		AD	-	-	-	-	-	-	-
**ADSP Extension**	443	CN	160	41 (25.6 %)	22 (53.7 %)	77.9 (6.9)	1 (0.6 %)	0 (0.00 %)	77.0 (-)
		AD	283	134 (47.3 %)	63 (47.0 %)	72.6 (7.0)	24 (8.5 %)	11 (45.8 %)	67.3 (6.3)
**MAYO**	1761	CN	1048	250 (23.9 %)	139 (55.6 %)	73.9 (4.9)	18 (1.7 %)	9 (50.0 %)	74.6 (2.7)
		AD	713	334 (46.8 %)	192 (57.5 %)	74.2 (4.7)	123 (17.3 %)	74 (60.2 %)	71.4 (5.0)
**MAYO2**	230	CN	160	31 (19.4 %)	16 (51.6 %)	82.3 (6.7)	2 (1.3 %)	1 (50.0 %)	88.0 (2.8)
		AD	70	31 (44.3 %)	23 (74.2 %)	74.3 (5.6)	5 (7.1 %)	4 (80.0 %)	67.8 (0.8)
**MIRAGE**	1098	CN	688	210 (30.5 %)	124 (59.0 %)	71.4 (7.1)	41 (6.0 %)	28 (68.3 %)	70.8 (6.9)
		AD	410	165 (40.2 %)	107 (64.8 %)	71.4 (6.2)	63 (15.4 %)	41 (65.1 %)	68.3 (5.4)
**MSBB**	180	CN	31	5 (16.1 %)	5 (100 %)	85.4 (3.6)	0 (0.0 %)	-	-
		AD	149	56 (37.6 %)	38 (67.9 %)	77.4 (7.8)	9 (6.0 %)	5 (55.6 %)	72.0 (7.4)
**MTC**	332	CN	113	18 (15.9 %)	13 (72.2 %)	73.0 (6.1)	1 (0.9 %)	1 (100 %)	73 (-)
		AD	219	99 (45.2 %)	55 (55.5 %)	73.6 (6.7)	27 (12.3 %)	10 (37.0 %)	68.7 (5.6)
**NIA-LOAD**	3050	CN	1303	412 (31.6 %)	242 (58.7 %)	72.0 (8.3)	45 (3.5 %)	26 (57.8 %)	70.3 (8.00)
		AD	1747	948 (54.3 %)	630 (66.5 %)	73.0 (6.1)	306 (17.5 %)	183 (59.8 %)	70.9 (6.4)
**OHSU**	511	CN	326	60 (18.4 %)	31 (51.7 %)	84.4 (7.6)	4 (1.2 %)	1 (25.0 %)	80.0 (5.6)
		AD	185	61 (33.0 %)	33 (54.1 %)	83.6 (6.3)	6 (3.2 %)	3 (50.0 %)	81.3 (8.3)

**ROSMAP**	1088	CN	528	82 (15.5 %)	62 (75.6 %)	84.3 (7.5)	5 (1.0 %)	5 (100 %)	87.0 (4.6)
		AD	560	174 (31.1 %)	128 (73.6 %)	81.9 (6.7)	14 (2.5 %)	12 (85.7 %)	78.4 (6.0)
**UM/VU/MSSM**	1652	CN	931	190 (20.4 %)	119 (62.6 %)	72.1 (7.7)	17 (1.8 %)	11 (64.7 %)	69.4 (5.1)
		AD	721	303 (42.0 %)	201 (66.3 %)	73.6 (6.6)	101 (14.0 %)	58 (57.4 %)	69.7 (5.9)
**UPITT**	2163	CN	870	144 (16.6 %)	89 (61.8 %)	73.4 (6.2)	9 (1.0 %)	5 (55.6 %)	75.6 (2.3)
		AD	1293	586 (45.3 %)	377 (64.3 %)	72.5 (6.5)	126 (9.7 %)	69 (54.8 %)	69.7 (5.8)
**WASHU**	434	CN	139	29 (20.9 %)	18 (62.1 %)	72.7 (7.4)	6 (4.3 %)	3 (50.0 %)	71.0 (5.8)
		AD	295	122 (41.4 %)	73 (59.8 %)	74.6 (7.7)	24 (8.1 %)	15 (62.5 %)	70.9 (7.2)
**WHICAP**	622	CN	548	102 (18.6 %)	64 (62.7 %)	80.9 (6.8)	6 (1.1 %)	3 (50.0 %)	71.0 (5.8)
		AD	74	14 (18.9 %)	10 (71.4 %)	85.4 (5.9)	1 (1.4 %)	0 (0.00 %)	70.9 (7.2)
**TOTAL**	**25120**	CN	12340	2707 (21.9 %)	1615 (59.7 %)	76.2 (8.4)	238 (1.9 %)	145 (60.9 %)	73.7 (7.4)
		AD	12780	5740 (44.9 %)	3411 (59.4 %)	73.8 (6.9)	1652 (12.9 %)	895 (54.2 %)	70.1 (6.2)
**REPLICATION SAMPLES**									
**ROTTERDAM**	10150	CN	8824	1868 (21.2 %)	1034 (55.4 %)	76.4 (9.3)	150 (1.7 %)	74 (49.3 %)	73.8 (8.0)
		AD	1326	411 (31.0 %)	282 (68.6 %)	82.2 (6.4)	86 (6.5 %)	50 (58.1 %)	78.2 (6.9)
**EADI**	8571	CN	6502	1141 (17.5 %)	672 (58.9 %)	79.6 (6.4)	62 (1.0 %)	45 (72.6 %)	77.8 (6.4)
		AD	2069	801 (38.7 %)	529 (66.0 %)	73.9 (7.4)	205 (9.9 %)	134 (65.4 %)	69.4 (5.9)
**EADB**	21860	CN	12295	2498 (20.3 %)	1431 (57.3 %)	72.4 (7.9)	195 (1.6 %)	107 (54.9 %)	70.3 (7.5)
		AD	9565	3912 (40.9 %)	2522 (64.5 %)	74.0 (7.2)	959 (10.0 %)	551 (57.5 %)	70.4 (6.8)

Table S5. Carrier frequencies of rs439401 across *APOE2/3/4 genotypes, in the haplotype reference consortium[41], versus the current study discovery sample, using *APOE* filtering approach 1.** Note enrichment of *APOE**4-rs439401 haplotypes for *APOE**3/4 and 4/4 strata in the discovery sample compared to the haplotype reference consortium. Related individuals were filtered down to third degree relatedness, retaining a single sample per relatedness cluster.

Haplotye Reference Consortum (HRC) r1.1

		rs439401 - T		
		**0**	**1**	**2**
APOE	**2/2**	156 (100 %)	0 (0.00 %)	0 (0.00 %)
	**2/3**	1712 (54.09 %)	1453 (45.91 %)	0 (0.00 %)
	**3/3**	4864 (29.76 %)	7840 (47.97 %)	3639 (22.27 %)
	**2/4**	643 (99.69 %)	2 (0.31 %)	0 (0.00 %)
	**3/4**	3327 (53.44 %)	2889 (46.40 %)	10 (0.16 %)
	**4/4**	625 (99.21 %)	5 (0.79 %)	0 (0.00 %)

Discovery samples (including related individuals)

		rs439401 - T		
		**0**	**1**	**2**
APOE	**2/2**	78 (97.50 %)	1 (1.25 %)	1 (1.25 %)
	**2/3**	1063 (52.31 %)	962 (47.34 %)	7 (0.34 %)
	**3/3**	3327 (27.67 %)	6012 (50.00 %)	2687 (22.34%)
	**2/4**	588 (97.84 %)	13 (2.16 %)	0 (0.00 %)
	**3/4**	4342 (51.48 %)	4060 (48.13 %)	33 (0.39 %)
	**4/4**	1856 (98.25 %)	28 (1.48 %)	5 (0.26 %)

Discovery samples (excluding related individuals)

		rs439401 - T		
		**0**	**1**	**2**
APOE	**2/2**	75 (98.68 %)	1 (1.32 %)	0 (0.00 %)
	**2/3**	989 (52.36 %)	894 (47.33 %)	6 (0.32 %)
	**3/3**	3018 (27.42 %)	5513 (50.10 %)	2472 (22.47 %)
	**2/4**	508 (98.07 %)	10 (1.93 %)	0 (0.00 %)
	**3/4**	3825 (51.44 %)	3584 (48.20 %)	27 (0.36 %)
	**4/4**	1606 (98.35 %)	22 (1.35 %)	5 (0.31 %)

Table S6. Associations of rs439401 with case-control status in supporting *APOE3/4 and 3/3 stratified analyses, using *APOE* filtering approach 1.**

	Genotype distributions			AD Case-Control regression	
**Group/model**	**CN, carrier No. / Total No. (%)**	**AD, carrier No. / Total No. (%)**	**CN - AD, MAF (%)**	**OR (95% CI)**	**P-value**
**rs439401 - T allele tested**					
***APOE* *3/4 - additive model**					
Discovery	1320 / 2702 (48.9 %)	2774 / 5734 (48.4 %)	24.8 % - 24.3 %	0.97 (0.88, 1.06)	0.45
***APOE* *3/3 - additive model**					
Discovery	5424 / 7519 (72.1 %)	3276 / 4508 (72.7 %)	47.4 % - 47.2 %	1.00 (0.94, 1.05)	0.86

Table S7. Duplicate concordance across samples for rs439401. Only genotype data that passed *APOE* filtering approach 1 and indicated European ancestry were used for these analyses (thus largely matching to samples considered in main analyses). In this subset, the total number of unique subjects with duplicates samples was N = 3804. Note there is subject overlap in the three respective analyses indicated in the table.

Duplicates across SNP microarray samples (unique N = 1431)

Concordant

APOE	rs439401 - T: 0	1	2
2/2	6	0	0
2/3	72	62	0
3/3	182	340	167
2/4	27	0	0
3/4	240	223	1
4/4	106	4	0

Discordant

APOE	rs439401 - T: 0	1	2
2/2	0	0	0
2/3	0	0	0
3/3	0	0	0
2/4	0	0	0
3/4	0	0	0
4/4	0	0	0

Duplicates across NGS samples (unique N = 704)

Concordant

APOE	rs439401 - T: 0	1	2
2/2	0	0	0
2/3	0	1	0
3/3	12	11	3
2/4	1	0	0
3/4	5	7	0
4/4	0	0	0

Discordant

APOE	rs439401 - T: 0	1	2
2/2	0	0	0
2/3	0	0	0
3/3	0	0	0
2/4	0	0	0
3/4	0	0	0
4/4	0	0	0

Duplicates between SNP microarray and WGS samples (unique N = 2463)

Concordant

APOE	rs439401 - T: 0	1	2
2/2	6	0	0
2/3	114	118	1
3/3	365	647	307
2/4	53	1	0
3/4	368	344	4
4/4	131	1	0

Discordant

APOE	rs439401 - T: 0	1	2
2/2	0	0	0
2/3	0	0	0
3/3	1*	1*	0
2/4	0	0	0
3/4	0	0	0
4/4	0	0	0

*Indicates 1 individual

Table S8. Cohort and *APOE* genotype details of *APOE4/4 subjects in the discovery carrying rs439401.** All shown participants were included in analyses using *APOE* filtering approach 1. Notably, one subject previously had *APOE**4/4 status called from the prior ADSP WES but now had a dubious call from the new ADSP WES data (first line in table), suggesting the subject is in fact an *APOE**3/4 carrier, which was also the provided and imputed *APOE* genotype.

Cohort	rs439401_T	final *APOE* determined for approach 1	prv_apoe (from demographics)	wgs_apoe (old ADSP extension + AMP-AD WGS)	wes_apoe (old ADSP discovery)	Subjects excluded in approach 2	imp_apoe (TOPMed imputation)	rs429359_R2 (TOPMed imputation)	wgs_apoe (new ADSP WGS + AMP-AD WGS)	wes_apoe (new ADSP WES)	rs429359_DP (ADSP read depth)	rs7412_DP (ADSP read depth)	rs429358_AD (ADSP allelic distribution T,C)	rs7412_AD (ADSP allelic distribution C,T)
**CONTROLS**														
ACT	1	44	34		44	excluded	34	0.92		?4	11	3	1,10	3,0
ADC1	1	44	44			excluded	34	0.93	34		40	33	15,25	33,0
ADC3	1	44	44			included	44	0.99						
ADC7	1	44	44			included	44	0.99	44		48	38	0,48	38,0
NIA-LOAD	1	44	44			excluded	34	0.94						
MIRAGE	2	44	44			excluded	33	0.90						
MIRAGE	2	44	44			excluded	33	0.92						
MIRAGE	1	44	44			excluded	34	0.92						
MIRAGE	2	44	44			excluded	33	0.92						
MIRAGE	1	44	44			excluded	34	0.92						
MIRAGE	1	44	44			excluded	34	0.92						
MIRAGE	1	44	44			excluded	33	0.92						
UPITT	1	44	44			excluded	33	1						
WASHU	1	44	44			excluded	33	0.93	33		36	20	36,0	20,0
**CASES**														
ADC1	1	44	44			included	44	0.93						
ADC1	1	44	44			excluded	34	0.93						
ADC4	1	44	44			included	44	0.99						
ADC7	1	44	44			included	44	0.99	44		66	35	0,66	35,0
ADC7	1	44	44			included	44	0.99						
ADC7	1	44	44			included	44	9.99						
ADDNEURO	1	44	44			excluded	33	0.98						
ADNI	1	44	44	44		included	44	0.99	44		16	25	0,16	25,0
NIA-LOAD	1	44	44			included	44	0.94						
NIA-LOAD	1	44	44			included	44	0.94		44	24	9	0,24	9,0
NIA-LOAD	1	44	44			included	34	0.94		44	28	6	0,28	6,0
MAYO	1	44	44			included	44	0.92						
MIRAGE	1	44	44			excluded	34	0.92						
MIRAGE	1	44	44			excluded	34	0.92						
MIRAGE	1	44	44			excluded	34	0.92						
UPITT	1	44	44			excluded	34	1						
UPITT	2	44	44			excluded	33	1						
UM-VU-MSSM	1	44	44			included	44	0.94						
UM-VU-MSSM	2	44	44			excluded	33	0.98						

Table S9. Concordance rate between provided and TOPMed imputed *APOE2/3/4 status across cohorts.** Note the rs7412 and rs429358 imputation (R2) scores for MIRAGE_370 and MIRAGE610. Very similar scores were observed for ADM_Q, ROSMAP_1B, and ROSMAP_1T, yet concordance rates between provided and imputed *APOE* were higher for those cohorts/arrays.

Cohort/Array	Concordance rate provided versus imputed *APOE*	Participants with provided *APOE* (N)	Participants with imputed *APOE* (N)	Participants with provided and imputed *APOE* (N)	rs429358 R^2	rs7412 R^2
ACT	95.37%	2581	2709	2551	91.82%	91.25%
ADC1	94.70%	2731	2662	2662	93.28%	89.79%
ADC2	95.23%	926	924	922	93.19%	90.89%
ADC3	99.34%	1526	1517	1517	98.70%	99.94%
ADC4	99.81%	1051	1037	1034	98.72%	99.96%
ADC5	99.59%	1224	1223	1223	98.52%	99.71%
ADC6	99.92%	1333	1329	1329	98.73%	99.98%
ADC7	99.72%	1462	1454	1454	98.59%	99.73%
ADM_O	98.70%	308	314	307	98.14%	93.51%
ADM_Q	88.45%	278	328	277	88.33%	80.21%
ADNI_1	93.13%	757	742	742	93.03%	87.97%
ADNI_DOD	100.00%	203	184	183	98.05%	99.96%
ADNI_O25	99.37%	812	796	796	99.13%	99.55%
ADNI_OE	100.00%	360	349	348	98.54%	99.98%
LOAD	94.90%	5220	5194	5194	93.79%	90.51%
MAYO_1	93.59%	2066	2046	2013	91.50%	89.01%
MAYO_2	99.67%	312	309	307	99.26%	99.80%
MIRAGE_370	81.46%	337	386	329	89.07%	83.05%
MIRAGE_610	82.50%	938	1102	937	91.99%	83.24%
MTC	98.78%	409	530	409	98.56%	99.79%
OHSU	91.41%	636	638	629	88.34%	91.87%
ROSMAP_1B	93.22%	1120	1097	1091	90.45%	80.38%
ROSMAP_1T	94.13%	581	580	579	91.39%	82.10%
ROSMAP_2C	98.69%	381	382	381	98.06%	99.98%
ROSMAP_3BU	98.20%	424	452	389	98.88%	99.93%
UPITT	96.82%	2436	2363	2359	99.53%	99.61%
UVM_A	95.89%	1147	1150	1144	94.10%	91.39%
UVM_B	95.22%	857	864	857	98.03%	99.98%
WASHU	92.22%	668	670	668	92.93%	92.63%
WHICAP	97.20%	642	647	642	98.35%	97.22%

Table S10. Discordance rate between *APOE* genotypes from different sources. Discordance rates in *APOE**4/4 carriers was determined only for provided *APOE**4/4 subjects.

Full sample

discordance rate = 4.3%

	imputed *APOE*					
**provided *APOE***	**2/2**	**2/3**	**2/4**	**3/3**	**3/4**	**4/4**
**2/2**	68	5	1	2	2	0
**2/3**	10	1859	14	108	18	1
**2/4**	0	21	521	7	31	0
**3/3**	0	80	5	11501	182	13
**3/4**	0	23	11	352	7837	32
**4/4**	0	0	1	13	135	1708

discordance rate = 2.8%

	imputed *APOE*					
**wgs *APOE***	**2/2**	**2/3**	**2/4**	**3/3**	**3/4**	**4/4**
**2/2**	8	1	0	0	0	0
**2/3**	0	214	0	19	1	0
**2/4**	0	0	43	1	4	0
**3/3**	0	4	0	1322	9	0
**3/4**	0	1	2	24	696	1
**4/4**	0	0	0	0	2	115

discordance rate = 0.9%

	provided *APOE*					
**wgs *APOE***	**2/2**	**2/3**	**2/4**	**3/3**	**3/4**	**4/4**
**2/2**	11	0	0	0	0	0
**2/3**	0	256	0	0	0	0
**2/4**	0	0	61	0	2	0
**3/3**	0	2	1	1538	10	0
**3/4**	0	2	1	6	883	0
**4/4**	0	0	0	0	1	138

***APOE* *4/4 sample (determined through *APOE* filtering approach 1)**

discordance rate = 7.9%

	imputed *APOE*					
**provided *APOE***	**2/2**	**2/3**	**2/4**	**3/3**	**3/4**	**4/4**
**2/2**	-	-	-	-	-	-
**2/3**	-	-	-	-	-	-
**2/4**	-	-	-	-	-	-
**3/3**	-	-	0	0	0	1
**3/4**	-	-	0	0	1	8
**4/4**	-	-	1	12	134	1708

discordance rate = 1.7%

	imputed *APOE*					
**wgs *APOE***	**2/2**	**2/3**	**2/4**	**3/3**	**3/4**	**4/4**
**2/2**	-	-	-	-	-	-
**2/3**	-	-	-	-	-	-
**2/4**	-	-	-	-	-	-
**3/3**	-	-	-	-	-	-
**3/4**	-	-	-	-	-	-
**4/4**	-	-	0	0	2	115

discordance rate = 0.7%

	provided *APOE*					
**wgs *APOE***	**2/2**	**2/3**	**2/4**	**3/3**	**3/4**	**4/4**
**2/2**	-	-	-	-	-	-
**2/3**	-	-	-	-	-	-
**2/4**	-	-	-	-	-	-
**3/3**	-	-	-	-	-	-
**3/4**	-	-	-	-	-	-
**4/4**	-	-	-	0	1	138

***APOE* *4/4 cases sample (determined through *APOE* filtering approach 1)**

discordance rate = 6.8%

	imputed *APOE*					
**provided *APOE***	**2/2**	**2/3**	**2/4**	**3/3**	**3/4**	**4/4**
**2/2**	-	-	-	-	-	-
**2/3**	-	-	-	-	-	-
**2/4**	-	-	-	-	-	-
**3/3**	-	-	0	0	0	1
**3/4**	-	-	0	0	0	7
**4/4**	-	-	1	5	104	1511

discordance rate = 1.9%

	imputed *APOE*					
**wgs *APOE***	**2/2**	**2/3**	**2/4**	**3/3**	**3/4**	**4/4**
**2/2**	-	-	-	-	-	-
**2/3**	-	-	-	-	-	-
**2/4**	-	-	-	-	-	-
**3/3**	-	-	-	-	-	-
**3/4**	-	-	-	-	-	-
**4/4**	-	-	0	0	14	146

discordance rate = 0.8%

	provided *APOE*					
**wgs *APOE***	**2/2**	**2/3**	**2/4**	**3/3**	**3/4**	**4/4**
**2/2**	-	-	-	-	-	-
**2/3**	-	-	-	-	-	-
**2/4**	-	-	-	-	-	-
**3/3**	-	-	-	-	-	-
**3/4**	-	-	-	-	-	-
**4/4**	-	-	-	0	1	128

***APOE* *4/4 controls sample (determined through *APOE* filtering approach 1)**

discordance rate = 15.8%

	imputed *APOE*					
**provided *APOE***	**2/2**	**2/3**	**2/4**	**3/3**	**3/4**	**4/4**
**2/2**	-	-	-	-	-	-
**2/3**	-	-	-	-	-	-
**2/4**	-	-	-	-	-	-
**3/3**	-	-	-	-	-	-
**3/4**	-	-	-	0	1	1
**4/4**	-	-	-	7	30	197

discordance rate = 0%

	imputed *APOE*					
**wgs *APOE***	**2/2**	**2/3**	**2/4**	**3/3**	**3/4**	**4/4**
**2/2**	-	-	-	-	-	-
**2/3**	-	-	-	-	-	-
**2/4**	-	-	-	-	-	-
**3/3**	-	-	-	-	-	-
**3/4**	-	-	-	-	-	-
**4/4**	-	-	-	0	0	9

discordance rate = 0%

	provided *APOE*					
**wgs *APOE***	**2/2**	**2/3**	**2/4**	**3/3**	**3/4**	**4/4**
**2/2**	-	-	-	-	-	-
**2/3**	-	-	-	-	-	-
**2/4**	-	-	-	-	-	-
**3/3**	-	-	-	-	-	-
**3/4**	-	-	-	-	-	-
**4/4**	-	-	-	-	0	10

***APOE* *4/4-rs439401 haplotype cases sample (determined through *APOE* filtering approach 1)**

discordance rate = 47.4%

	imputed *APOE*					
**provided *APOE***	**2/2**	**2/3**	**2/4**	**3/3**	**3/4**	**4/4**
**2/2**	-	-	-	-	-	-
**2/3**	-	-	-	-	-	-
**2/4**	-	-	-	-	-	-
**3/3**	-	-	-	-	-	-
**3/4**	-	-	-	-	-	-
**4/4**	-	-	-	3	6	10

discordance rate = 25%

	imputed *APOE*					
**wgs *APOE***	**2/2**	**2/3**	**2/4**	**3/3**	**3/4**	**4/4**
**2/2**	-	-	-	-	-	-
**2/3**	-	-	-	-	-	-
**2/4**	-	-	-	-	-	-
**3/3**	-	-	-	-	-	-
**3/4**	-	-	-	-	-	-
**4/4**	-	-	0	0	1	3

discordance rate = 0%

	provided *APOE*					
**wgs *APOE***	**2/2**	**2/3**	**2/4**	**3/3**	**3/4**	**4/4**
**2/2**	-	-	-	-	-	-
**2/3**	-	-	-	-	-	-
**2/4**	-	-	-	-	-	-
**3/3**	-	-	-	-	-	-
**3/4**	-	-	-	-	-	-
**4/4**	-	-	-	-	-	4

***APOE* *4/4-rs439401 haplotype controls sample (determined through *APOE* filtering approach 1)**

discordance rate = 84.6%

	imputed *APOE*					
**provided *APOE***	**2/2**	**2/3**	**2/4**	**3/3**	**3/4**	**4/4**
**2/2**	-	-	-	-	-	-
**2/3**	-	-	-	-	-	-
**2/4**	-	-	-	-	-	-
**3/3**	-	-	-	-	-	-
**3/4**	-	-	-	0	1	0
**4/4**	-	-	-	6	5	2

discordance rate = -

	imputed *APOE*					
**wgs *APOE***	**2/2**	**2/3**	**2/4**	**3/3**	**3/4**	**4/4**
**2/2**	-	-	-	-	-	-
**2/3**	-	-	-	-	-	-
**2/4**	-	-	-	-	-	-
**3/3**	-	-	-	-	-	-
**3/4**	-	-	-	-	-	-
**4/4**	-	-	-	-	-	-

discordance rate = -

	provided *APOE*					
**wgs *APOE***	**2/2**	**2/3**	**2/4**	**3/3**	**3/4**	**4/4**
**2/2**	-	-	-	-	-	-
**2/3**	-	-	-	-	-	-
**2/4**	-	-	-	-	-	-
**3/3**	-	-	-	-	-	-
**3/4**	-	-	-	-	-	-
**4/4**	-	-	-	-	-	-

References

1. Kunkle BW, Grenier-Boley B, Sims R, et al. Genetic meta-analysis of diagnosed Alzheimer's disease identifies new risk loci and implicates Aβ, tau, immunity and lipid processing. *Nat Genet*. 2019;51(3):414-430. doi:10.1038/s41588-019-0358-2

2. Bis JC, Jian X, Chen BWK, et al. Whole exome sequencing study identifies novel rare and common Alzheimer's-Associated variants involved in immune response and transcriptional regulation. *Mol Psychiatry*. 2020;25:1859-1875. doi:10.1038/s41380-018-0112-7

3. Beecham GW, Bis JC, Martin ER, et al. The Alzheimer's disease sequencing project: Study design and sample selection. *Neurol Genet*. 2017;3:e194. doi:10.1212/NXG.0000000000000194

4. Wang M, Beckmann ND, Roussos P, et al. The Mount Sinai cohort of large-scale genomic, transcriptomic and proteomic data in Alzheimer's disease. *Sci Data*. 2018;5:180185. doi:10.1038/sdata.2018.185

5. De Jager PL, Ma Y, McCabe C, et al. A multi-omic atlas of the human frontal cortex for aging and Alzheimer's disease research. *Sci Data*. 2018;5:180142. doi:10.1038/sdata.2018.142

6. Crane PK, Foroud T, Montine TJ, Larson EB. Alzheimer's Disease Sequencing Project Discovery and Replication criteria for cases and controls: data from a community-based prospective cohort study with autopsy follow-up. *Alzheimers Dement*. 2017;13(12):1410-1413. doi:10.1016/j.jalz.2017.09.010.

7. Mckhann G, Drachman D, Folstein M, Katzman R, Price D, Mckhann G. Clinical Diagnosis of Alzheimer's Disease: Report of the NINCDS-ADRDA Work Group Under the Auspices of Derpartment of Health and Human Services Task Force on Alzheimer's Disease. *Neurology*. 1984;34:27-28. doi:10.1212/01.wnl.0000400650.92875.cf

8. Bell CC. DSM-IV: Diagnostic and Statistical Manual of Mental Disorders. *JAMA*. 1994;272(10):828-829.

9. American Psychiatric Association. Diagnostic and Statistical Manual of Mental Disorders 5th edn. *Am Psychiatr Assoc*. Published online 2013.

10. American Psychiatric Association. Diagnostic and Statistical Manual of Mental Disorders 4th edn. *Am Psychiatr Assoc*. Published online 1994.

11. Hughes CP, Berg L, Danziger WL, Coben LA, Martin RL. A new clinical scale for the staging of dementia. *Br J Psychiatry*. 1982;140(6):566-572. doi:10.1192/bjp.140.6.566

12. Braak H, Braak E. Neuropathological stageing of Alzheimer-related changes. *Acta Neuropathol*. 1991;82:239-259. doi:10.1109/ICINIS.2015.10

13. Mirra SS, Heyman A, McKeel D, et al. The Consortium to Establish a Registry for Alzheimer's Disease (CERAD). Part II. Standardization of the neuropathologic assessement of Alzheimer's disease. *Neurology*. 1991;41:479-486. doi:10.1017/CBO9781107415324.004

14. Hyman BT, Trojanowski JQ. Editorial on Consensus Recommendations for the Postmortem Diagnosis of Alzheimer Disease from the National Institute on Aging and the Reagan Institute

Working Group on Diagnositic Criteria for the Neuropathological Assessment of Alzheimr Disease. *J Neuropathol Exp Neurol*. 1997;56(10):1095-1097.

15. Folstein MF, Folstein SE, McHugh PR. “Mini-Mental State” A Practical Method for Grading the Cognitive State of Patients for the Clinician. *J Psychiatr Res*. 1975;12:189-198. doi:10.3744/snak.2003.40.2.021

16. Roccaforte WH, Burke WJ, Bayer BL, Wengel SP. Validation of a Telephone Version of the Mini-Mental State Examination. *J Am Geriatr Soc*. 1992;40:697-702. https://seirei-univ.repo.nii.ac.jp/?action=repository_action_common_download&item_id=821&item_no=1&attribute_id=20&file_no=1

17. McKhann GM, Knopman DS, Chertkow H, et al. The diagnosis of dementia due to Alzheimer’s disease: Recommendations from the National Institute on Aging- Alzheimer’s Association workgroups on diagnostic guidelines for Alzheimer’s disease. *Alzheimers Dement*. 2011;7(3):263-269. doi:10.1016/j.jalz.2011.03.005.

18. Albert MS, DeKosky ST, Dickson D, et al. The diagnosis of mild cognitive impairment due to Alzheimer’s disease: Recommendations from the National Institute on Aging- Alzheimer’s Association workgroups on diagnostic guidelines for Alzheimer’s disease. *Alzheimers Dement*. 2011;7(3):270-279. doi:10.1016/j.jalz.2011.03.008.

19. Hyman BT, Phelps CH, Beach TG, et al. National Institute on Aging – Alzheimer’s Association guidelines for the neuropathologic assessment of Alzheimer’s disease. *Alzheimer’s Dement*. 2012;8(1):1-13. doi:10.1016/j.jalz.2011.10.007

20. Morris JC, Weintraub S, Chui HC, et al. The Uniform Data Set (UDS): Clinical and cognitive variables and descriptive data from Alzheimer disease centers. *Alzheimer Dis Assoc Disord*. 2006;20(4):210-216. doi:10.1097/01.wad.0000213865.09806.92

21. Weintraub S, Salmon D, Mercaldo N, et al. The Alzheimer’s Disease Centers’ Uniform Data Set (UDS) The Neuropsychologic Test Battery. *Alzheimer Dis Assoc Disord*. 2009;23(2):91-101. https://www.alz.washington.edu

22. Beekly DL, Ramos EM, Lee WW, et al. The National Alzheimer’s Coordinating Center (NACC) Database: The Uniform Data Set. *Alzheimer Dis Assoc Disord*. 2007;21(3):249-258. doi:10.1097/WAD.0b013e318142774e

23. Besser LM, Kukull WA, Teylan MA, et al. The revised national Alzheimer’s coordinating center’s neuropathology form-available data and new analyses. *J Neuropathol Exp Neurol*. 2018;77(8):717-726. doi:10.1093/jnen/nly049

24. Besser L, Kukull W, Knopman DS, et al. Version 3 of the national Alzheimer’s coordinating center’s uniform data set. *Alzheimer Dis Assoc Disord*. 2018;32(4):351-358. doi:10.1097/WAD.0000000000000279

25. Bennett DA, Schneider JA, Buchman AS, De Leon CM, Bienias JL, Wilson RS. The rush memory and aging project: Study design and baseline characteristics of the study cohort. *Neuroepidemiology*. 2005;25(4):163-175. doi:10.1159/000087446

26. Bennett DA, Wilson RS, Schneider JA, et al. Natural history of mild cognitive impairment in older

persons. *Neurology*. 2002;59(2):198-205. doi:10.1212/WNL.59.2.198

27. Schneider JA, Arvanitakis Z, Bang W, Bennett DA. Mixed brain pathologies account for most dementia cases in community-dwelling older persons. *Neurology*. 2007;69(24):2197-2204. doi:10.1212/01.wnl.0000271090.28148.24

28. Hinrichs AS, Karolchik D, Baertsch R, et al. The UCSC Genome Browser Database: update 2006. *Nucleic Acids Res*. 2006;34(Database issue):590-598. doi:10.1093/nar/gkj144

29. Deelen P, Bonder MJ, Van Der Velde KJ, et al. Genotype harmonizer: Automatic strand alignment and format conversion for genotype data integration. *BMC Res Notes*. 2014;7:901. doi:10.1186/1756-0500-7-901

30. Franke L, de Kovel CGF, Aulchenko YS, et al. Detection, Imputation, and Association Analysis of Small Deletions and Null Alleles on Oligonucleotide Arrays. *Am J Hum Genet*. 2008;82(6):1316-1333. doi:10.1016/j.ajhg.2008.05.008

31. Eichler EE, Nickerson DA, Altshuler D, et al. Completing the map of human genetic variation. *Nature*. 2007;447(7141):161-165. doi:10.1038/447161a

32. Karczewski KJ, Francioli LC, Tiao G, et al. The mutational constraint spectrum quantified from variation in 141,456 humans. *Nature*. 2020;581(May). doi:10.1038/s41586-020-2308-7

33. Chen CY, Pollack S, Hunter DJ, Hirschhorn JN, Kraft P, Price AL. Improved ancestry inference using weights from external reference panels. *Bioinformatics*. 2013;29(11):1399-1406. doi:10.1093/bioinformatics/btt144

34. Auton A, Abecasis GR, Altshuler DM, et al. A global reference for human genetic variation. *Nature*. 2015;526(7571):68-74. doi:10.1038/nature15393

35. Conomos MP, Miller MB, Thornton TA. Robust inference of population structure for ancestry prediction and correction of stratification in the presence of relatedness. *Genet Epidemiol*. 2015;39(4):276-293. doi:10.1002/gepi.21896

36. NIAGADS. NG00067 – ADSP Umbrella. Published 2021. https://dss.niagads.org/datasets/ng00067/

37. Zhu C, Farrell J, Kuzma A, Schellenberg G, Farrer L. Precision genotyping of APOE from whole genome sequencing. In: *American Society of Human Genetics Congress*. ; 2019.

38. Naj AC, Jun G, Beecham GW, et al. Common variants at MS4A4/MS4A6E, CD2AP, CD33 and EPHA1 are associated with late-onset Alzheimer’s disease. *Nat Genet*. 2011;43(5):436-443. doi:10.1038/ng.801

39. Wattmo C, Londos E, Minthon L. Risk factors that affect life expectancy in alzheimer’s disease: A 15-year follow-up. *Dement Geriatr Cogn Disord*. 2014;38(5-6):286-299. doi:10.1159/000362926

40. Kuzma A, Valladares O, Cweibel R, et al. NIAGADS: The NIA Genetics of Alzheimer’s Disease Data Storage Site. *Alzheimer’s Dement*. 2016;12(11):1200-1203. doi:10.1016/j.jalz.2016.08.018

41. McCarthy S, Das S, Kretzschmar W, et al. A reference panel of 64,976 haplotypes for genotype imputation. *Nat Genet*. 2016;48(10):1279-1283. doi:10.1038/ng.3643

185
